# Programming heterometallic 4f–4f′ helicates under thermodynamic control: the circle is complete†

**DOI:** 10.1039/d4dt00610k

**Published:** 2024-03-01

**Authors:** Charlotte Egger, Laure Guénée, Neel Deorukhkar, Claude Piguet

**Affiliations:** a Department of Inorganic and Analytical Chemistry, University of Geneva 30 quai E. Ansermet CH-1211 Geneva 4 Switzerland Claude.Piguet@unige.ch; b Laboratory of Crystallography, University of Geneva 24 quai E. Ansermet CH-1211 Geneva 4 Switzerland

## Abstract

Three non-symmetrical segmental ligand strands L4 can be wrapped around a linear sequence of one Zn^2+^ and two trivalent lanthanide cations Ln^3+^ to give quantitatively directional [ZnLn_2_(L4)_3_]^8+^ triple-stranded helicates in the solid state and in solution. NMR speciations in CD_3_CN show negligible decomplexation at a millimolar concentration and the latter helicate can be thus safely considered as a preorganized *C*_3_-symmetrical *HHH*-[(L4_3_Zn)(Ln^A^)_(2−*n*)_(Ln^B^)_*n*_]^8+^ platform in which the thermodynamic properties of (i) lanthanide permutation between the central N_9_ and the terminal N_6_O_3_ binding sites and (ii) exchange processes between homo- and heterolanthanide helicates are easy to access (Ln = La, Eu, Lu). Deviations from statistical distributions could be programmed by exploiting specific site recognition and intermetallic pair interactions. Considering the challenging La^3+ ^: Eu^3+^ ionic pair, for which the sizes of the two cations differ by only 8%, a remarkable excess (70%) of the heterolanthanide is produced, together with a preference for the formation of the isomer where the largest lanthanum cation lies in the central N_9_ site ([(La)(Eu)] : [(Eu)(La)] = 9 : 1). This rare design and its rational programming pave the way for the preparation of directional light-converters and/or molecular Q-bits at the (supra)molecular level.

## Abstract 2

The use of pair interactions and specific site affinities provides 63% of the heterotrimetallic *HHH*-[(L4_3_Zn)LaEu]^8+^ helicate in solution where statistics predicts only 25% of this isomer under thermodynamic control for a stoichiometric ratio of 1 : 1 : 1.

## Graphical abstract

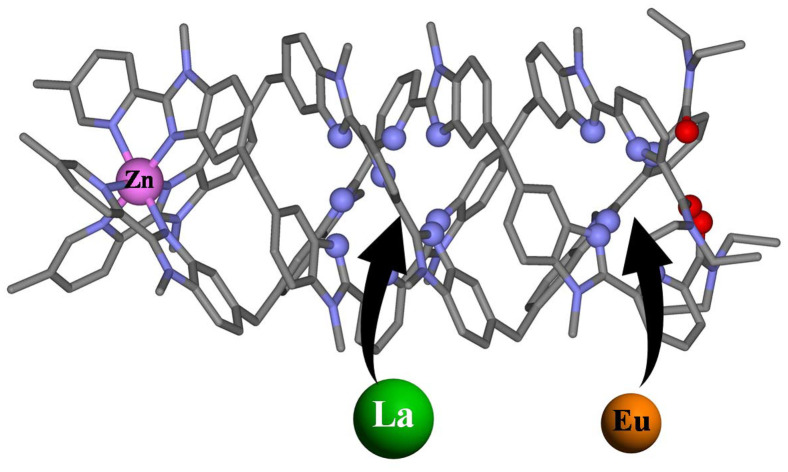

## Introduction

**Scheme 1:**
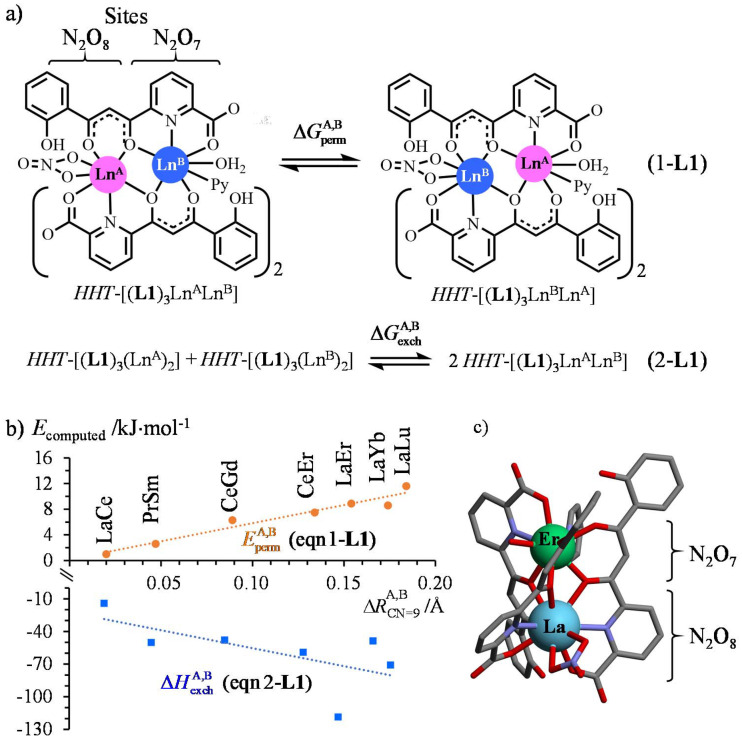
a) Permutation (eqn 1-L1) and exchange (eqn 2-L1) equilibria proposed for the heterometallic *HHT*-[(L1)_3_Ln^A^Ln^B^(NO_3_)(H_2_O)(pyridine)] complexes and (b) associated gas-phase DFT-computed energy changes.^36^
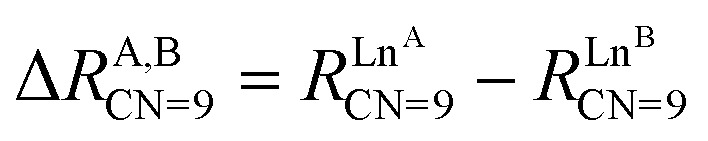
 corresponds to the difference of nine-coordinate lanthanide ionic radii in the considered metallic pair.^51^ The linear trendlines are only a guide to the eye. (c) Crystal structure of *HHT*-[(L1)_3_LaEr(NO_3_)(H_2_O)(pyridine)].^35^

The lack of radial node characterizing the atomic orbitals having *n* − *l* = 1 (*n* and *l* are the principal and azimuthal quantum numbers, respectively), often referred to as the primogenic effect,^1^ significantly contributes to the inner-shell pseudo-atomic character of the valence 4f orbitals in the trivalent lanthanides Ln^3+^ ([Xe]4f^*n*^, *n* = 0–14).^2–4^ The main consequence for chemistry results in a (very) similar reactivity of Ln^3+^ along the complete lanthanide series, which is only smoothly modulated by the stepwise 1% contraction of the ionic radii between adjacent elements.^5–8^ The molecular recognition of specific Ln^3+^, beyond the standard electrostatic trend,^5–8^ is therefore mainly lacking for coordination complexes in solution.^9^ This prevents the planned design of heterometallic polynuclear f–f′ assemblies in solution under thermodynamic control, except for some rare reports of deviations from statistical distributions in solution.^10–14^ Consequently, the planned implementation of pure heterometallic f–f′ molecular complexes in solution mainly relies on multistep strategies which exploit the kinetic inertness provided by the complexation of Ln^3+^ within rigid, highly preorganized and often anionic receptors (Fig. A1-1 in Appendix 1, see ESI†).^15–26^ By broadening the perspective, one realizes that energy barriers, responsible for kinetic inertness, thermodynamic stability and selectivity, may greatly benefit from long-range stacking interactions accompanying crystallization processes,^27–29^ and serendipitous pure f–f′ assemblies are therefore reported in crystalline materials (Fig. A1-2 in Appendix 1, see ESI†).^30,31–40^ The use of statistical doping has made it possible to temporarily circumvent these limits and myriads of doped ionic solids,^41^ nanoparticles,^42,43^ metal–organic frameworks^30,44,45^ or solid-state molecular aggregates and clusters^46–49^ have been prepared and explored for improving lighting and optical signaling in materials. However, the recent recognition^35^ that the (very) minor magnetic coupling operating between two different lanthanide Kramer's ions in non-statistical molecular heterometallic f–f′ entities represents a keystone for the design of basic information units in quantum computers, that is Q-bits,^50^ reactivates the efforts aiming at the preparation of pure (*i.e.* non-statistical) heterometallic lanthanide molecular complexes under thermodynamic control. With this in mind, Aromi and coworkers have developed some remarkable and versatile scaffolds consisting of fused didentate β-diketonate and tridentate 2,6-dipicolinate units for the formation of different binding pockets, which display size discriminating effects along the lanthanide series in the solid state, when three ligands are wrapped around two (Scheme 1)^35–37^ or more trivalent cations (Fig. A1–2d in Appendix 1, see ESI†).^38–40^

Focusing on *HHT*-[(L1)_3_Ln^A^Ln^B^(NO_3_)(H_2_O)(pyridine)],^36^ impressive deviations (1 ≤ *E*^A,B^_perm_ ≤ 11 kJ mol^−1^ and −120 ≤ Δ*H*^A,B^_exch_ ≤ −14 kJ mol^−1^, Scheme 1b) from the expected statistical distributions (Δ*G*^A,B, stat^_perm_ = 0 kJ mol^−1^ for eqn 1-L1 and Δ*G*^A,B, stat^_exch_ = −*RT* ln(4) = −3.4 kJ mol^−1^ for eqn 2-L1) could be predicted by gas-phase DFT calculations assuming that the molecular structures observed in the crystalline state (Scheme 1c) are pertinent to initiate gas-phase modelling.^36^

**Scheme 2:**
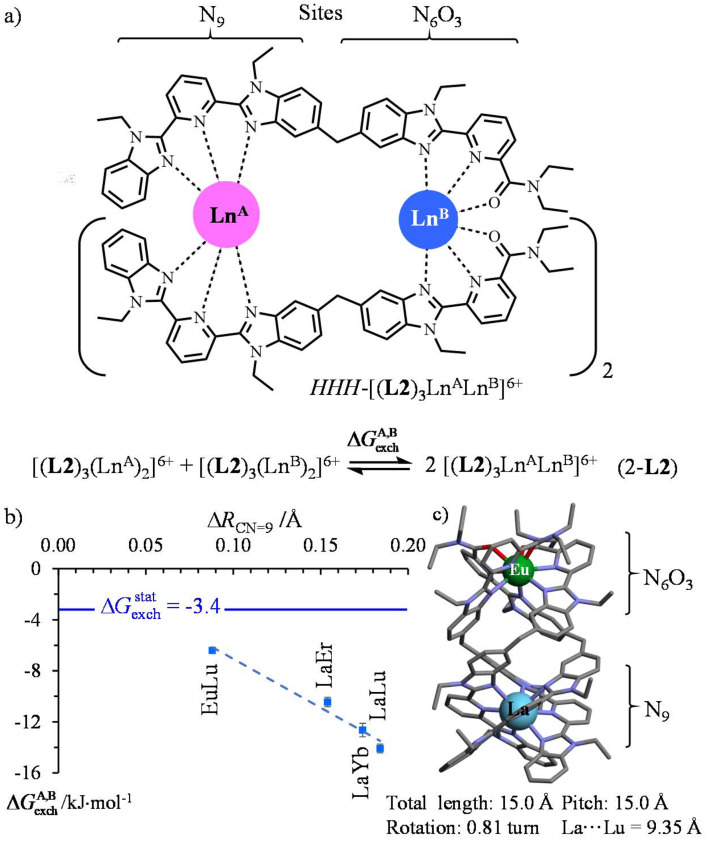
a) Thermodynamic exchange equilibria (eqn 2-L2) and (b) associated free energy changes of the triple-stranded [(L2)_3_(Ln^A^)_(2−*n*)_(Ln^B^)_*n*_]^6+^ helicates (*x* = 0, 1, 2) in CD_3_CN at room temperature.^10^ Only the *HHH* isomer is shown, but mixtures of *HHH* and *HHT* isomers exist in solution. 
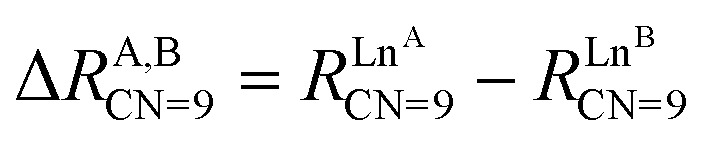
 corresponds to the difference of nine-coordinate lanthanide ionic radii in the metallic pair.^51^ The dashed linear trendline is only a guide to the eye. (c) Crystal structure of *HHH*-[(L2)_3_LaEu](ClO_4_)_6_.^10^

To the best of our knowledge, related experimental thermodynamic data are available only for homometallic/heterometallic exchange eqn (2-L2) operating in the triple-stranded [(L2)_3_(Ln^A^)_(2−*n*)_(Ln^B^)_*n*_]^6+^ helicates (*n* = 0, 1, 2) in acetonitrile (Scheme 2a).^10^ One can note that the measured free energy changes −6 ≤ Δ*G*^A,B^_exch_ ≤ −14 kJ mol^−1^ in solution (Scheme 2b) drastically differ from the optimistic gas-phase DFT-predictions reported for *HHT*-[(L1)_3_Ln^A^Ln^B^(NO_3_)(H_2_O)(pyridine)] (Scheme 1b).^36^

The unavoidable ligand permutation, which interconverts *C*_3_-symmetrical *HHH*-[(L2)_3_(Ln^A^)_(2−*n*)_(Ln^B^)_*n*_]^6+^ with its *C*_1_-symmetrical *HHT*-[(L2)_3_(Ln^A^)_(2−*n*)_(Ln^B^)_*n*_]^6+^ counterpart, severely limits further thermodynamic analysis and the target (trivial) lanthanide exchange process involving two well-defined and different binding sites proposed by Aromi in eqn (1-L1) for *HHT*-[(L1)_3_Ln^A^Ln^B^(NO_3_)(H_2_O)(pyridine)] (Scheme 1) escaped quantification in solution with L2.

**Scheme 3:**
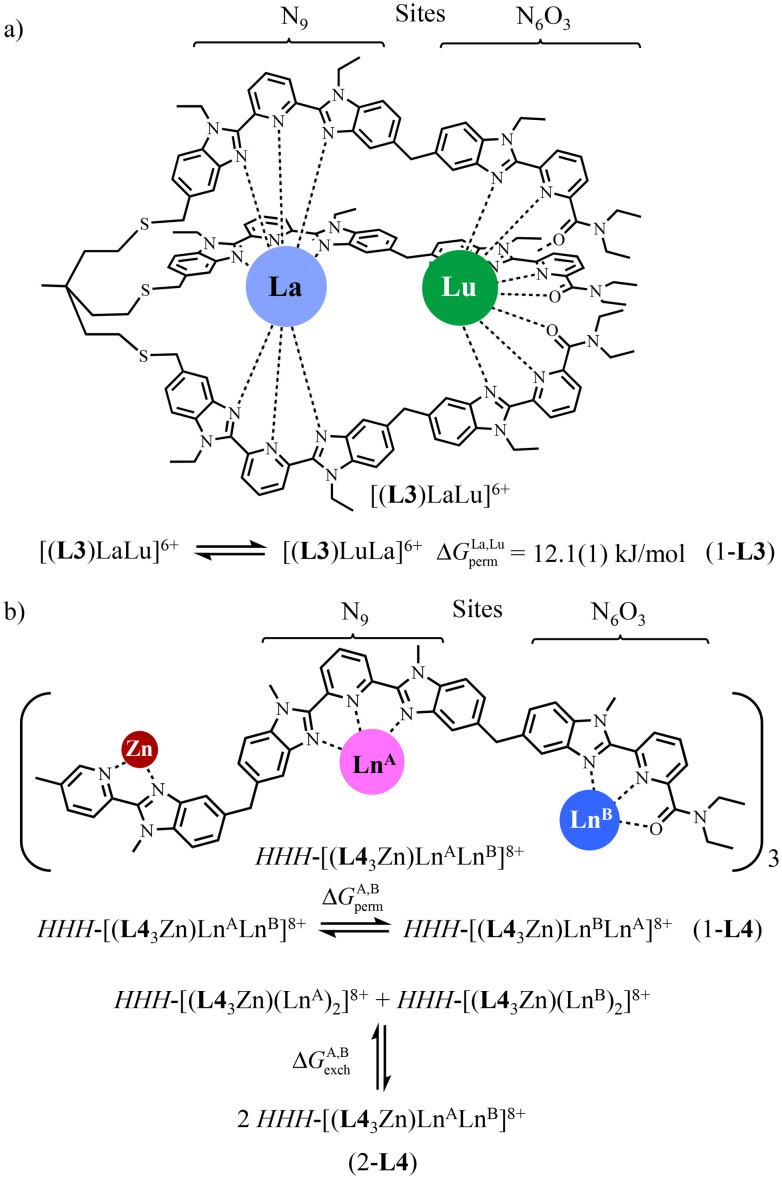
a) Thermodynamic permutation equilibrium (eqn 1-L3) estimated for [(L3)LaLu]^6+^ in CD_3_CN at room temperature.^52^ (b) Removal of *HHH*/*HHT* isomerism in the dinuclear-lanthanide triple-stranded helicates *HHH*-[(L4_3_Zn)(Ln^A^)(Ln^B^)]^8+^ by using [Zn(pyridine-benzimidazole)_3_] as a preorganized non-covalent tripod.^57^

Connecting the three strands to a non-labile tripod seems to be the obvious choice for avoiding ligand scrambling and permutation, but structural constraints imposed by the helical wrapping of the strands required considerable synthetic efforts and delicate chemical design, which have been only approached once for the preorganized heterometallic dinuclear *C*_3_-symmetrical [L3LaLu]^6+^ podate (Scheme 3a).^52–55^ The free energy change estimated for the searched La : Lu permutation summarized in eqn (1-L3) amounts to Δ*G*^La, Lu^_perm_ = 12.1(1) kJ mol^−1^ (CD_3_CN at room temperature), but it entirely relies on the assumption that the mixing rule Δ*E*^mix^_1–2_ = Δ*E*^La, Lu^_1–2_ − ½(Δ*E*^La, La^_1–2_ + Δ*E*^Lu, Lu^_1–2_) = 0 is obeyed.^56^

Rejuvenated by the challenge of preparing pure f–f′ complexes under thermodynamic control, which are required for the preparation of molecular magnetic Q-bits,^36^ we have re-engaged the fight with the use of a terminal non-covalent *HHH*-[Zn(pyridine-benzimidazole)_3_] tripod which preorganizes the three strands for their concomitant efficient binding around two successive Ln^3+^ guests in two well-defined and different coordination sites. We therefore propose in this work to close the loop with a novel and efficient preparation of the segmental ligand L4 so that one can access the thermodynamically self-assembled *HHH*-[(L4_3_Zn)Ln^A^Ln^B^]^8+^ helicates for which both lanthanide permutation eqn (1-L4) and lanthanide exchange eqn (2-L4) can be deciphered in solution (Scheme 3b).^57^

## Results and discussion

### Preparation and structures of L4 and its homolanthanide triple-helical complexes *HHH*-[(L4_3_Zn)Ln_2_](CF_3_SO_3_)_8_ (Ln = La, Eu, Lu)

Taking advantage of previous synthetic efforts,^57^ the strategy for preparing the segmental didentate–tridentate–tridentate ligand L4 has been optimized (Scheme A2-1 in Appendix 2). L4 could be thus efficiently prepared in seven steps with a global yield of 5.6% from commercially available 2,5-lutidine (1a), together with previously synthesized 4,4′-methylenebis(*N*-methyl-2-nitroaniline) (2c),^58^ 6-(diethylcarbamoyl)picolinic acid (3d)^59^ and *N*^2^,*N*^2^-diethyl-*N*^6^-methyl-*N*^6^-(4-(4-(methylamino)-3-nitrobenzyl)-2-nitrophenyl)pyridine-2,6-dicarboxamide (8).^59^ The ^1^H-NMR of the free ligand L4 shows a total of 32 signals accounting for the 55 protons, which confirms an average *C*_s_-symmetry on the NMR timescale (Fig. S1†). The absence of NOE correlations between the pyridine *meta*-protons and the benzimidazole methyl groups implies *anti* conformations of the donor N-atoms of the α,α′-diimine units, which are typical of unbound polyaromatic benzimidazole-pyridine segments in solution,^59–61^ a trend further confirmed in the crystal structure of L4·C_3_H_8_O (Fig. S2, S3 and Tables S1–S3†). The subsequent reaction of the segmental ligand L4 (3.0 eq., 15 mM) with stoichiometric amounts of Zn(CF_3_SO_3_)_2_ (1.0 eq., 5 mM) and Ln(CF_3_SO_3_)_3_ (2.0 eq., 10 mM, Ln = La(iii), Eu(iii), Lu(iii)) in CDCl_3_/CD_3_CN (1 : 2) quantitatively affords the target homolanthanide *HHH*-[(L4_3_Zn)Ln_2_]^8+^ triple-stranded helicates within a few hours according to global equilibrium 3 (Fig. S4–S6†).3
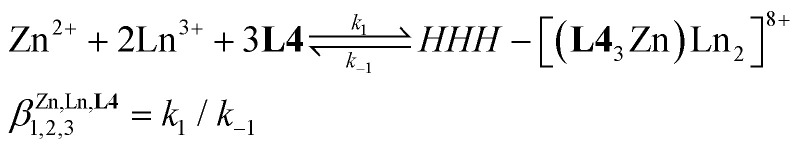


**Fig. 1:**
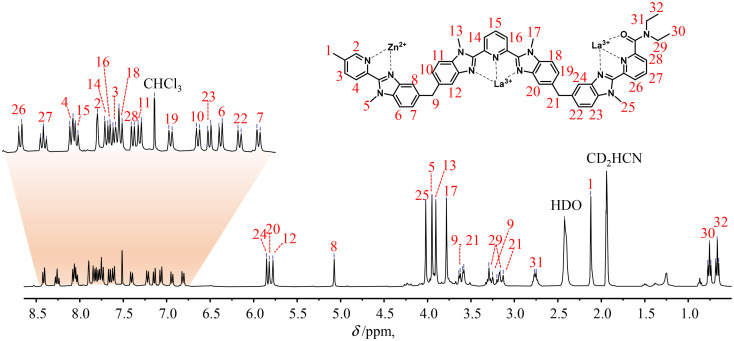
^1^H-NMR spectrum of the *HHH*-[(L4_3_Zn)La_2_]^8+^ complex (2 : 1 CD_3_CN/CDCl_3_, 400 MHz, 298 K). The aromatic region was expanded for clarity.

The final ^1^H-NMR spectra of *HHH*-[(L4_3_Zn)Ln_2_]^8+^ show the exclusive (>98%) formation of a single *C*_3_-symmetrical helicate in solution for each assembly process (Fig. 1 and S7–S15†). A consequence of the close vicinity of the three wrapped strands is highlighted in the diamagnetic complexes *HHH*-[(L4_3_Zn)Ln_2_]^8+^ (Ln = La, Lu) by the unusually low chemical shifts (4.95 ≤ *δ* ≤ 5.85 ppm) recorded for the aromatic protons 8, 12, 20 and 24 which are located in the shielding region of a benzimidazole ring of an adjacent strand (Fig. 1 and S7†).

One further notes that the small ionic radius of Lu(iii) leads to a tighter wrapping of the ligand strands, which shifts the ^1^H-NMR signals of the benzimidazole protons 8, 12, 20 and 24 from 5.07 ≤ *δ* ≤ 5.85 ppm in *HHH*-[(L4_3_Zn)La_2_]^8+^ toward 4.95 ≤ *δ* ≤ 5.34 ppm in *HHH*-[(L4_3_Zn)Lu_2_]^8+^ (Fig. S16 and Table S4†). The replacement of the diamagnetic La^3+^ or Lu^3+^ cations with fast-relaxing paramagnetic Eu^3+^ in *HHH*-[(L4_3_Zn)Eu_2_]^8+^ results in the expected^11,59^ downfield shifts of the singlet signals of the benzimidazole protons 12, 20 and 24 (12.00 ≤ *δ* ≤ 14.62 ppm) which are located close to the Eu^3+^ paramagnetic centers in the final complex (Table S5 and Fig. S8†). Additional proof for the formation of the desired complex is provided by the high-resolution ESI-TOF spectra recorded in acetonitrile, which display peaks corresponding to the series of triflate adducts *HHH*-{[(L4_3_Zn)Ln_2_](CF_3_SO_3_)_*n*_}^(8−*n*)+^ (*n* = 2, 4, 5; Ln = La, Eu), although only at low relative intensities (Fig. S17 and S19†). The theoretical isotopic patterns nicely match the experimental peaks (Fig. S18 and S20†). The rest of the peaks, which have been assigned by their isotopic patterns, correspond to partial dissociation of one or more ligand strands and/or of one or more metal ions (Tables S6 and S7†). Due to the complete lack of ^1^H-NMR evidence supporting significant decomplexation of *HHH*-[(L4_3_Zn)Ln_2_]^8+^ complexes (Ln = La, Eu, Lu) at millimolar concentrations in solution, the dissociation observed in the HR-MS spectra are assigned to the gas-phase reaction accompanying the ESI process.

Considering the labile character of both Zn(ii) and Ln(iii) (Ln = La, Eu, Lu) in solution, the formation of the desired trinuclear homolanthanide helicates within a few hours contrasts sharply with the fast (within seconds) self-assembly of the analogous dinuclear dimetallic [ZnLa(**6**)_3_]^5+^ complex, where the shorter segmental ligand **6** (Fig. A2–1 in Appendix 2, see ESI†) corresponds to L4 after the removal of the central tridentate 2,6-bis(benzimidazole)pyridine unit.^59^ The elongation of the ligand strand in L4 increases the complexity of the supramolecular system, which in turn increases the number of possible intermediates and reversible steps required before converging toward the thermodynamic products.^62,63^ An ultra-simplistic consideration of the whole self-assembly process as being modeled with equilibrium (3) predicts a negligible dissociation rate constant 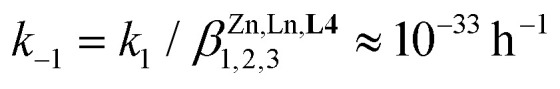 since (i) *k*_1_ can be estimated around 1000 M^−6^ h^−1^ when one takes into account a characteristic time constant of 2 hours for the formation of 50% of the final *HHH*-[(L4_3_Zn)Ln_2_]^8+^ helicate in solution at a millimolar concentration and (ii) 
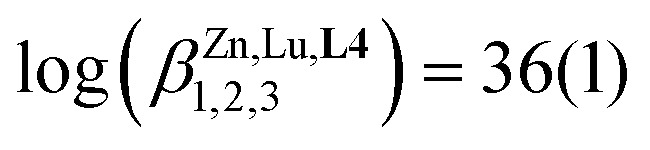
 for *HHH*-[(L4_3_Zn)Lu_2_]^8+^ in acetonitrile.^57^

**Fig. 2:**
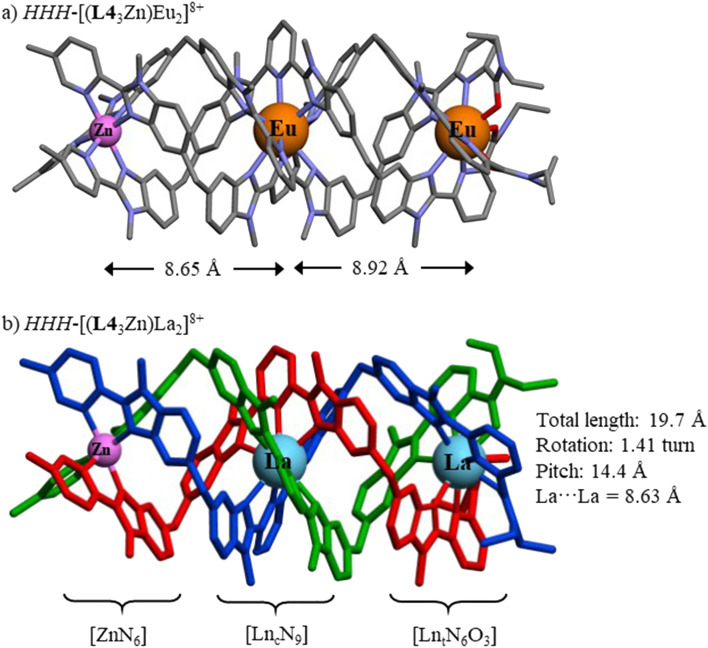
Molecular structures of (a) *HHH*-[(L4_3_Zn)Eu_2_]^8+^ as observed in the crystal structure of [(L4_3_Zn)Eu_2_](CF_3_SO_3_)_8_·12(C_3_H_8_O) with highlighted intermetallic distances (color code: C = grey, N = blue, O = red) and (b) *HHH*-[(L4_3_Zn)La_2_]^8+^ as found in the crystal structure of [(L4_3_Zn)La_2_](CF_3_SO_3_)_8_·7.25(CH_3_CN) with the three wrapped strands shown in different colors.

Slow diffusion of isopropanol and diethyl ether, respectively, into solutions of *HHH*-[(L4_3_Zn)Eu_2_]^8+^ and *HHH*-[(L4_3_Zn)La_2_]^8+^ in acetonitrile yielded single crystals of [(**L4**_3_Zn)Eu_2_](CF_3_SO_3_)_8_·12(C_3_H_8_O) and [(**L4**_3_Zn)La_2_](CF_3_SO_3_)_8_·7.25(CH_3_CN) of suitable quality for X-ray diffraction analysis (Fig. 2 and S21–S28, Tables S8–S25†). The X-ray structure of analogous [(L4_3_Zn)Lu_2_](CF_3_SO_3_)_8_ was reported previously,^57^ but the limited quality of the datasets collected at this time (despite using a synchrotron radiation source) did not allow a detailed analysis of the structure.

**Fig. 3:**
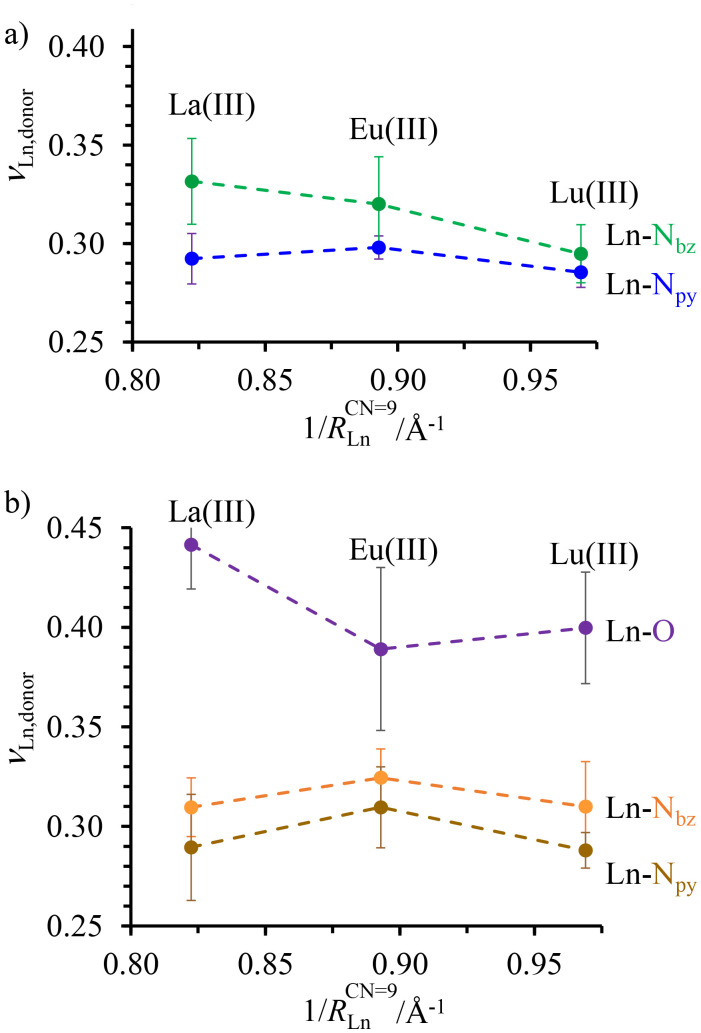
Variation of the average bond valences *ν*_Ln-donor_ calculated with eqn (4) for (a) Ln(iii)_c_ in the central N_9_ binding site and (b) Ln(iii)_t_ in the terminal N_6_O_3_ site as a function of the inverse of the nine-coordinate lanthanide ionic radii^51^ in *HHH*-[(L4_3_Zn)Ln_2_]^8+^ (Ln = La(iii), Eu(iii), and Lu(iii)). Standard deviations of the averages are shown with vertical error bars. The dashed traces are only a guide to the eye.

The crystal structures unambiguously confirm the formation of the desired homolanthanide d–f–f triple-stranded helicates, showing the three metal ions almost linearly aligned along the pseudo-*C*_3_ axis (average Zn–Ln_c_–Ln_t_ angle 176(2)°, Fig. 2a and Table S26†) and the three ligand strands helically wrapped around them in a head-to-head-to-head fashion (Fig. 2b). The intermetallic distances between adjacent cations average to 8.7(2) Å (Table S26†) lie within the range of distances previously reported in a number of polynuclear lanthanide helicates based on similar *oligo*-pyridyl-benzimidazole scaffolds (Scheme 2c and Fig. 3).^10,59,64–68^ The tighter wrapping of the ligand strands around the smallest lanthanide ions, previously mentioned when discussing the large upfield shift of the ^1^H-NMR signals of the benzimidazole protons in *HHH*-[(L4_3_Zn)Lu_2_]^8+^ (Fig. S16†), leads to increasingly longer intermetallic distances as the size of the coordinated lanthanide ions reduces (entries 1 and 2 in Table S26†). While the average helical pitches (14.1–14.4 Å) do not vary significantly between the three complexes (entry 8 in Table S26†), and closely mirror those measured for previous homolanthanide helicates (Fig. S29†), a detailed analysis of each helical portion defined by eight almost parallel facial planes F1–F8 (Fig. S22, S23 and S25–S28†) showed significant local variations of the wrapping mode (Table S25†). In all three helicates, the tight rotations observed around each binding site alternate with severely relaxed helical twists associated with the diphenylmethane linkers. The helicity within the terminal [Ln_t_N_6_O_3_] binding site is the most irregular out of the three coordinating units due to the difference in angular rotation caused by the carboxamide-pyridine moiety on one side, and by the pyridine-benzimidazole motif on the other side.^59^

The geometries of the coordination spheres around the three cations were analyzed with the software SHAPE,^69–71^ the final scores of which point toward a pseudo-octahedral arrangement for the [ZnN_6_] units (Table S26, entries 11 and 12†). Due to the poor stereochemical preferences of the lanthanide ions,^72,73^ comparable SHAPE scores are obtained for various geometries of the nine-coordinate Ln^3+^ sites. At the more flexible terminal [Ln_t_N_6_O_3_] sites, the lowest scores for all three lanthanides point to the tricapped trigonal prism geometry (Table S26, entries 20 and 21†). In the central [Ln_c_N_9_] sites, a tricapped trigonal prism geometry is adopted by the largest La(iii) cation, while a spherical capped square antiprism geometry is observed around the smaller Eu(iii) and Lu(iii) cations (Table S26, entries 15 and 16†). One finally notices that the Zn–N bond distances do not vary significantly in the different *HHH*-[(L4_3_Zn)Ln_2_]^8+^ helicates (Ln = La, Eu, Lu; Fig. S30†). This implies sufficient flexibility within the wrapped strands for overcoming significant structural constraints accompanying the lanthanide contraction along the 4f-series.

As expected for the flexible scaffold found in *HHH*-[(L4_3_Zn)Ln_2_]^8+^, the Ln–N and Ln–O bond distances mirror the lanthanide contraction along the series (Fig. S30†),^51,74,75^ but a more detailed analysis of the Ln–N and Ln–O bond strengths, corrected for the lanthanide contraction, can be assessed by calculating the bond valences *ν*_ij_ with eqn (4), where *R*_ij_ is the bond valence parameter associated with a given set of metal ion i and donor atom j,^76–78^*d*_ij_ is the distance between the i–j pair, and *b* = 0.37 is a universal scaling constant (Fig. 3 and Table S27†).^79^

4 *ν*_*ij*_ = *e*^[(*R*_ij_−*d*_ij_)/*b*]^

The largest bond valences, diagnostic of the strongest metal–ligand affinities, were found between the lanthanides and the O-donors in the terminal N_6_O_3_ site (Fig. 3b). Interestingly, a sharp decrease of the Ln_t_–O bond valence observed when going from La(iii) to Eu(iii) is compensated by an increase of the Ln_t_–N_bz_ and Ln_t_–N_py_ interactions. The resulting concave trend detected for both Ln_t_–N_bz_ and Ln_t_–N_py_ bond valences in the terminal N_6_O_3_ site is not reproduced in the central N_9_ site (Fig. 3a), where the average Ln_c_–N_bz_ interaction decreases regularly throughout the series while the Ln_c_–N_py_ interaction remains roughly constant. Altogether, the terminal N_6_O_3_ site exhibits a weak preference for binding mid-range Ln^3+^ while the central N_9_ site penalizes the binding of the smaller lanthanides in *HHH*-[(L4_3_Zn)Ln_2_]^8+^, a tendency previously established for the related [LnN_9_] and [LnN_6_O_3_] sites found in the homolanthanide *D*_3_-symmetrical [(L5)_3_Ln_2_]^6+^ and [(L6)_3_Ln_2_]^6+^ helicates (Fig. S29†).^80^

### Formation and speciation of heterolanthanide triple-helical complexes *HHH*-[(L4_3_Zn)Ln^A^Ln^B^]^8+^ in solution (Ln^A^, Ln^B^ = La, Eu, Lu)

**Fig. 4:**
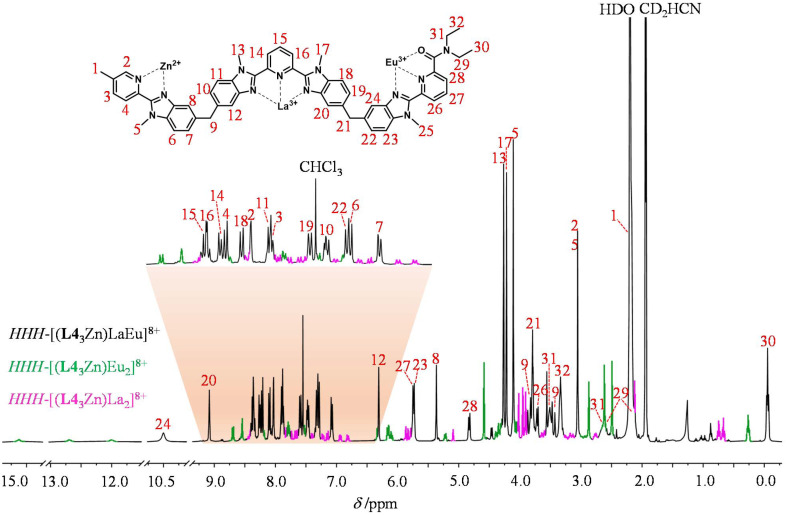
^1^H-NMR spectrum of a 1 : 1 : 1 : 3 mixture of Zn(CF_3_SO_3_)_2_, La(CF_3_SO_3_)_3_, Eu(CF_3_SO_3_)_3_ and **L4** at equilibrium (2 : 1 CD_3_CN/CDCl_3_, 400 MHz, 298 K). The aromatic region was expanded for clarity. The signals highlighted in green represent *HHH*-[(L4_3_Zn)Eu_2_]^8+^ and those in pink represent *HHH*-[(L4_3_Zn)La_2_]^8+^.

The reaction of the segmental ligand L4 (3.0 eq.) with a 1 : 1 : 1 mixture of Zn(CF_3_SO_3_)_2_ (1.0 eq.), La(CF_3_SO_3_)_3_ (1.0 eq.) and Eu(CF_3_SO_3_)_3_ (1.0 eq.) in a 1 : 2 mixture of CDCl_3_/CD_3_CN was followed by ^1^H-NMR until the equilibrium was reached (Fig. S31†). The comparison of the ^1^H-NMR spectrum of the final mixture with those of the free ligand L4 and the previously characterized homolanthanide complexes *HHH*-[(L4_3_Zn)La_2_]^8+^ and *HHH*-[(L4_3_Zn)Eu_2_]^8+^ demonstrates the formation of a single major new species displaying the characteristic features of a *C*_3_-symmetric triple-stranded helicate (Fig. S32†). The rest of the (minor) signals correspond to the homolanthanide complexes, with no trace of the free ligand (Fig. 4). One of the two possible heterolanthanide isomers strongly dominates the speciation (Table 1, second column), thus confirming that the two different lanthanide binding sites exhibit size-discriminating effects. With the help of correlation and NOE spectroscopy (Fig. S33†), the ^1^H-NMR spectrum of the main product could be fully assigned to *HHH*-[(L4_3_Zn)LaEu]^8+^, where specific paramagnetic-induced chemical shifts (Fig. S34†) ascertain that the Eu(iii) cation occupies the terminal N_6_O_3_ binding site in the major heterolanthanide isomer (Fig. 4). As expected, the high-resolution ESI-TOF spectrum of the mixture confirmed the co-existence of both the homo- and heterolanthanide complexes in the gas-phase (Fig. S35†).

**Table 1:** Speciation (mole fraction) at equilibrium following the reaction of L4 (3.0 eq., 15 mM) with a mixture of Zn(CF_3_SO_3_)_2_ (1.0 eq.), Ln^A^(CF_3_SO_3_)_3_ (1.0 eq.) and Ln^B^(CF_3_SO_3_)_3_ (1.0 eq.). Thermodynamic descriptors and related free energies associated with the permutation (eqn (5)) and exchange (eqn (6)) equilibria (1 : 2 mixture of CDCl_3_/CD_3_CN, 298 K)

Ln^A^–Ln^B^	La–Eu	Eu–Lu	La–Lu
Δ*R*_CN=9_^LnA^^, LnB^ /Å	0.096	0.088	0.184
*x*(*HHH*-[(L4_3_Zn)Ln^A^_2_]^8+^)	0.14(1)	0.28(2)	0.25(2)
*x*(*HHH*-[(L4_3_Zn)Ln^B^_2_]^8+^)	0.16(2)	0.37(4)	0.24(2)
*x*(*HHH*-[(L4_3_Zn)Ln^A^Ln^B^]^8+^)	0.63(5)	0.27(3)	0.46(3)
*x*(*HHH*-[(L4_3_Zn)Ln^B^Ln^A^]^8+^)	0.07(1)	0.08(1)	0.05(1)
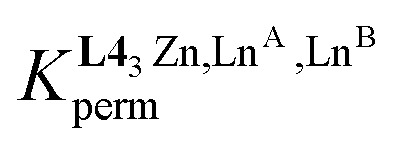	0.11(2)	0.30(5)	0.11(2)
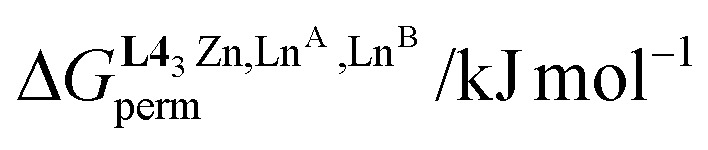	5.4(4)	3.0(4)	5.4(5)
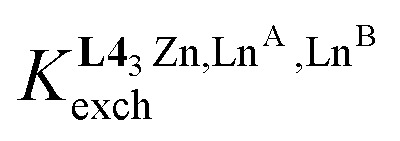	22(4)	1.2(2)	4.3(6)
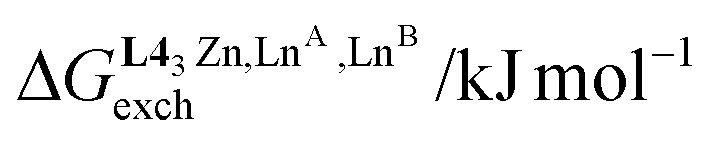	−7.5(4)	−0.4(4)	−3.6(4)
*u* ^mix^_1–2_	1.4(2)	0.46(8)	0.6(1)
Δ*E*^mix^_1–2_ /kJ mol^−1^	−0.8(4)	1.9(4)	1.2(5)

The formation of the *HHH*-[(L4_3_Zn)LaEu]^8+^ complex as the main product of the self-assembly of a 1 : 1 : 1 mixture of Zn(CF_3_SO_3_)_2_, La(CF_3_SO_3_)_3_, and Eu(CF_3_SO_3_)_3_ with 3.0 eq. of L4 is consistent with the stereochemical preference of the central N_9_ site for larger lanthanide ions and that of the terminal N_6_O_3_ site for smaller ones.^10,11,80^ In this context, replacing La(iii) with Lu(iii) in the mixture should make the coordination of the Eu(iii) cation now more favorable in the central N_9_ site made of three wrapped bis(benzimidazole)pyridine segments while the terminal N_6_O_3_ site should preferentially accommodate the smaller Lu(iii), hence yielding *HHH*-[(L4_3_Zn)EuLu]^8+^ as the major heterolanthanide isomer in solution. The latter prediction was confirmed by following the reaction of L4 (3.0 eq., 15 mM) with a 1eq : 1eq : 1eq mixture of Zn(CF_3_SO_3_)_2_, Eu(CF_3_SO_3_)_3_ and Lu(CF_3_SO_3_)_3_ in a 1 : 2 mixture of CDCl_3_/CD_3_CN with the help of ^1^H-NMR techniques. The ^1^H-NMR spectrum recorded at equilibrium (after 24 hours, Fig. S36†) showed non-negligible amounts of the homolanthanide helicates *HHH*-[(L4_3_Zn)Eu_2_]^8+^ and *HHH*-[(L4_3_Zn)Lu_2_]^8+^ together with one major set of unidentified peaks that corresponded to the heterolanthanide *HHH*-[(L4_3_Zn)EuLu]^8+^ isomer (Fig. S37–S39,† column 3 in Table 1).

Finally, the reaction of L4 (3.0 eq., 15 mM) with a 1 eq : 1 eq : 1 eq mixture of Zn(CF_3_SO_3_)_2_, La(CF_3_SO_3_)_3_ and Lu(CF_3_SO_3_)_3_ was the fastest to reach the equilibrium, showing little to no evolution in the ^1^H-NMR spectrum after only a few hours at room temperature (Fig. S40†). Similarly to the previous mixtures, two homolanthanide helicates *HHH*-[(L4_3_Zn)La_2_]^8+^ and *HHH*-[(L4_3_Zn)Lu_2_]^8+^ are formed, along with only one of the two possible heterolanthanide isomers (Fig. S41–S42,† column 4 in Table 1). The absence of the open-shell Eu(iii) probe in the mixture makes the assignment of the ^1^H-NMR spectrum of the heterolanthanide *HHH*-[(L4_3_Zn)LaLu]^8+^ complex harder since the chemical shifts of the protons surrounding the central and the terminal sites are not as different as with the paramagnetic helicates. However, the size difference between La(iii) and Lu(iii) has been shown to affect the tightness of the wrapping of the ligand strands (Fig. S16†), which results in ^1^H-NMR signals for the central isolated benzimidazole singlets which are diagnostic for the binding of the largest La(iii) cation in the central N_9_ site, while Lu(iii) lies in the terminal site in *HHH*-[(L4_3_Zn)LaLu]^8+^ (Fig. S42†). The permutated *HHH*-[(L4_3_Zn)LuLa]^8+^ isomer could not be detected in the final mixture and its mole fraction was thus set at the limit of accuracy (*x* ≤ 0.05) estimated for our ^1^H-NMR experimental setup (Table 1).

### Thermodynamic rationalization of the formation of heterolanthanide triple-helical complexes *HHH*-[(L4_3_Zn)Ln^A^Ln^B^]^8+^ in solution (Ln^A^, Ln^B^ = La, Eu, Lu)

**Scheme 4:**
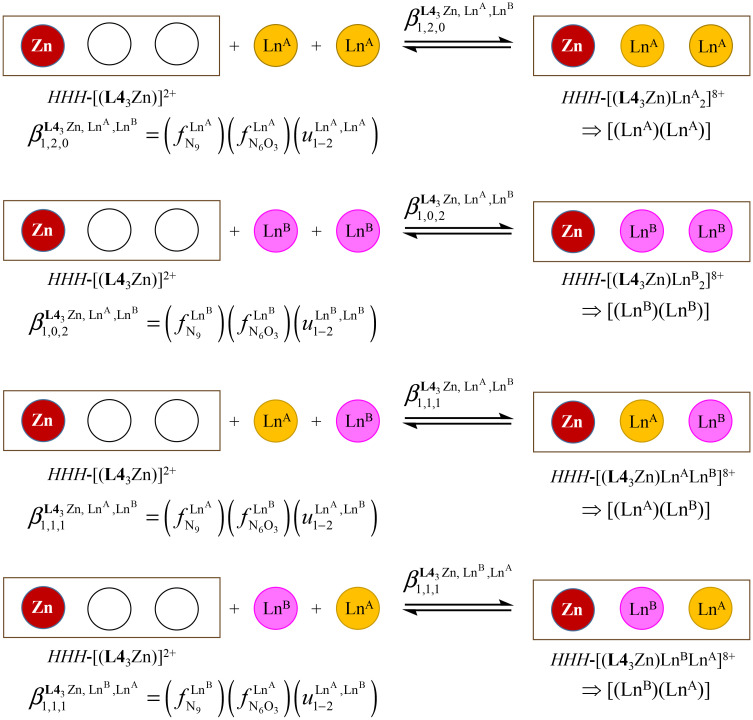
Microscopic thermodynamic formation constants 
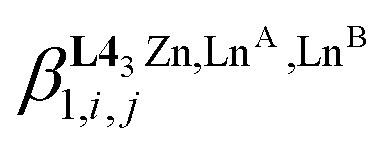
 for *HHH*-[(L4_3_Zn)(Ln^A^)_(2−*n*)_(Ln^B^)_*n*_]^8+^ (*n* = 0, 1, and 2) and their modeling with the site binding model.^81,82^ See main text for the definitions of *f*^Ln*j*^_*i*_ and *u*^Ln*i*^_1–2_, Ln^*j*^.

In the absence of significant complex dissociation at millimolar concentrations, as demonstrated for the stoichiometric mixing of L4 (3.0 eq.) with Zn(CF_3_SO_3_)_2_ (1.0 eq.), Ln^A^(CF_3_SO_3_)_3_ and Ln^B^(CF_3_SO_3_)_3_ (1.0 eq.) in solution, the four interconverting helicates *HHH*-[(L4_3_Zn)(Ln^A^)_(2−*n*)_(Ln^B^)_*n*_]^8+^ (*n* = 0, 1, 2) are related by the generic thermodynamic permutation equilibrium (1) (eqn 1-L*k* in Schemes 1–3, further generalized below as eqn (5)) and exchange equilibrium (2) (eqn 2-L*k* in Schemes 1–3, further generalized below as eqn (6)), where *HHH*-[(L4_3_Zn)]^2+^ is considered as a rigid platform for the complexation of Ln^A^ and Ln^B^ in the two appended and preorganized N_9_ and N_6_O_3_ binding sites (Scheme 4). The equilibrium concentrations are written between double vertical bars | | in eqn (5) and (6).5
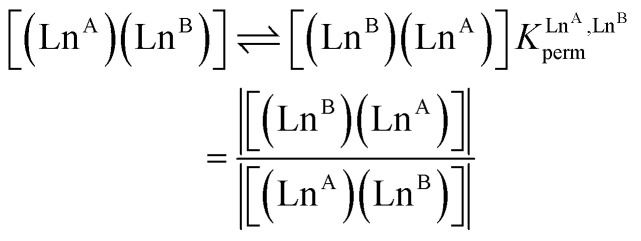
6
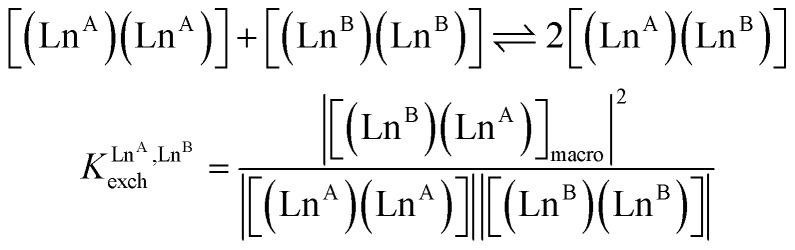


Focusing on *HHH*-[(L4_3_Zn)(Ln^A^)_(2−*n*)_(Ln^B^)_*n*_]^8+^, 
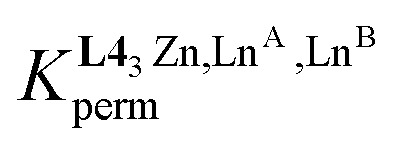
 (eqn (5)) and 
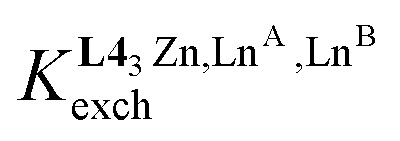
 (eqn (6)) can be modeled with the help of microscopic formation constants 
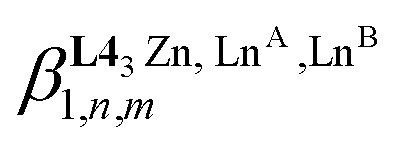
 to give eqn (7) and (8) within the frame of the site binding model, where *f*_i_^Ln^*j*^^ is the intermolecular microscopic affinity of the nine-coordinate site *i* for the entering lanthanide Ln^*j*^ in the preorganized *HHH*-[(L4_3_Zn)]^2+^ receptor and 
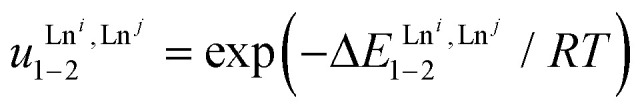
 is the Boltzmann factor measuring the intermetallic pair interactions 
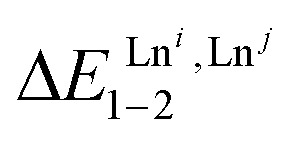
 operating between adjacent Ln^*i*^ and Ln^*j*^ cations in [(Ln^*i*^)(Ln^*j*^)] (Scheme 4).^81,82^7
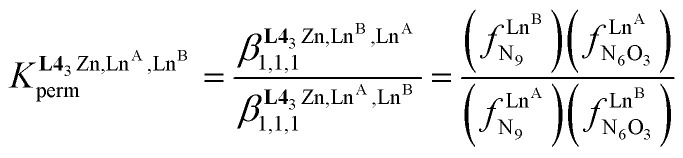
8
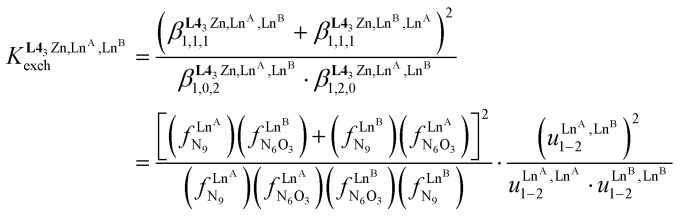


The last term of eqn (8) corresponds to the square of 
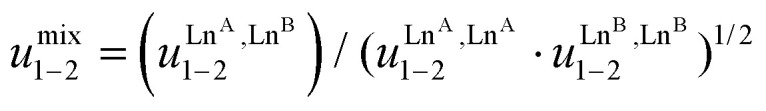
, which is related to the mixing energy Δ*E*^mix^_1–2_ in eqn (9).^56^9
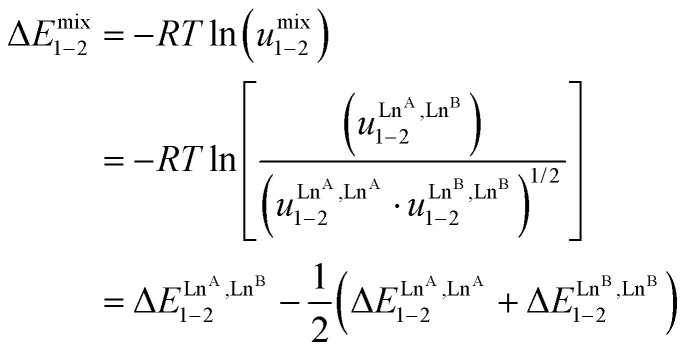
When Δ*E*^mix^_1–2_ = 0, the pair interaction energies obey the mixing rule 
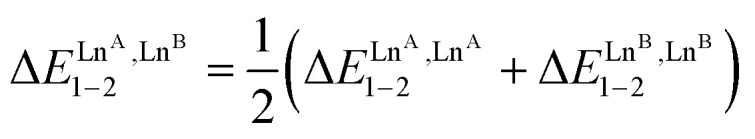
, which corresponds to a non-cooperative behavior and results in a random distribution of the two different metal ions among the coordination sites.^56^ Deviations from the mixing rule can be assigned to either cooperative processes (Δ*E*^mix^_1–2_ > 0), which are characterized by the clustering of identical metals along the strands, or anti-cooperative processes (Δ*E*^mix^_1–2_ < 0), which correspond to an alternation of the different metals.^56,81,82^

**Fig. 5:**
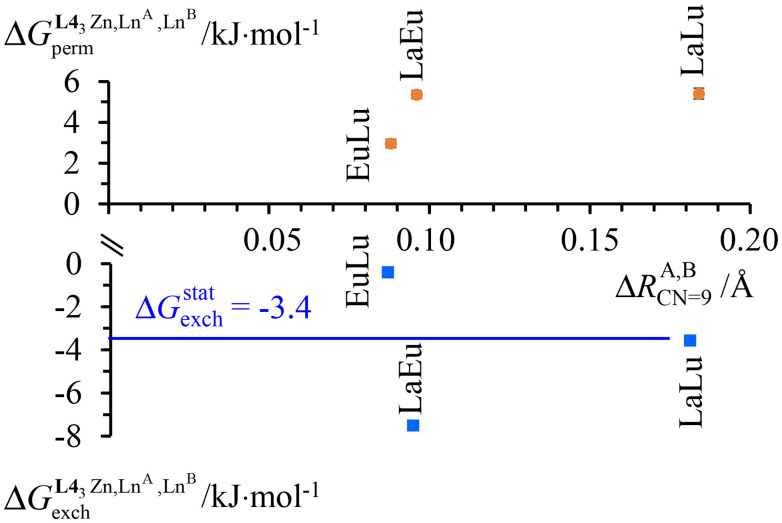
Free energies for permutation (
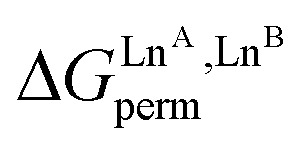
 in eqn (5), orange markers) and for exchange (
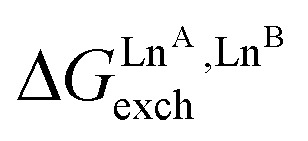
 in eqn (6), blue markers) observed in solution at room temperature for *HHH*-[(L4_3_Zn)(Ln^A^)(Ln^B^)]^8+^ (Table 2) as a function of the difference of the nine-coordinate lanthanide ionic radii (Δ*R*^A, B^_CN=9_).^51^ The full traces correspond to statistical behaviours.

The experimental permutation energies (orange markers) and exchange energies (blue markers) obtained for *HHH*-[(L4_3_Zn)(Ln^A^)(Ln^B^)]^8+^ (entries 7 and 9 in Table 1) are summarized in Fig. 5. One immediately notices the systematic positive permutation energies 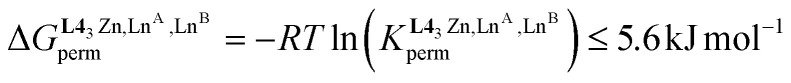 (top of Fig. 5), which reflect the preferred formation of the heterolanthanide isomer where the larger cation lies in the central N_9_ binding site and the smaller cation occupies the terminal N_6_O_3_ binding site (
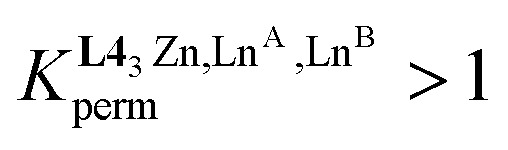
, eqn (7)). The combination of eqn (7) and (8), pertinent to 
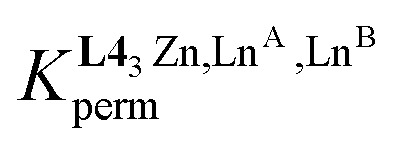
 and 
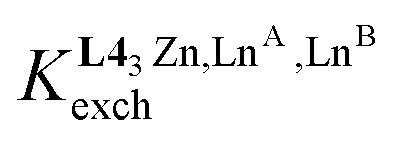
, provides an elegant experimental access to the balance of the intermetallic pair interactions as measured by *u*^mix^_1–2_ in eqn (10), and consequently to the associated mixing energies −0.8 ≤ Δ*E*^mix^_1–2_ ≤ 1.9 kJ mol^−1^ operating in *HHH*-[(L4_3_Zn)(Ln^A^)_(2−*n*)_(Ln^B^)_*n*_]^8+^ (*n* = 0, 1, 2; Table 1, entry 11).10
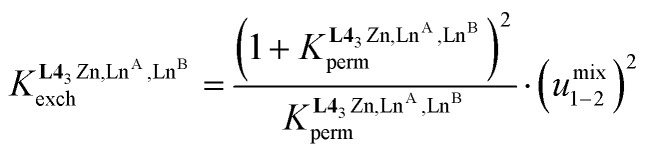


When the two different lanthanide cations are larger than Gd^3+^ (= belong to the first half of the lanthanide series), as illustrated in *HHH*-[(L4_3_Zn)(La)_(2−*n*)_(Eu)_*n*_]^8+^ (*n* = 0, 1, 2), then Δ*E*^La, Eu^_1–2_ < ½(Δ*E*^La, La^_1–2_ + Δ*E*^Eu, Eu^_1–2_) and the associated value of *u*^mix^_1–2_ = 1.4(2) boosts the formation of the heterolanthanide *HHH*-[(L4_3_Zn)(La)(Eu)]^8+^ complexes to reach 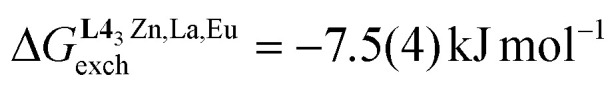, which lies much beyond the statistical value of Δ*G*^stat^_exch_ = −*RT* ln(4) = −3.4 kJ mol^−1^ (bottom of Fig. 5, blue trace). As soon as one lanthanide cation of the pair belongs to the second part of the lanthanide series, as exemplified in heterolanthanide [(Eu)(Lu)] and in [(La)(Lu)] helicates, the reverse situation occurs with 
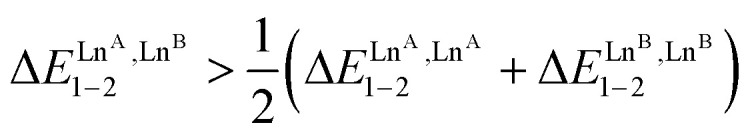
 and the balance of pair interactions tend to discard the formation of heterolanthanide *HHH*-[(L4_3_Zn)(Ln^A^)(Ln^B^)]^8+^ complexes.

**Scheme 5:**
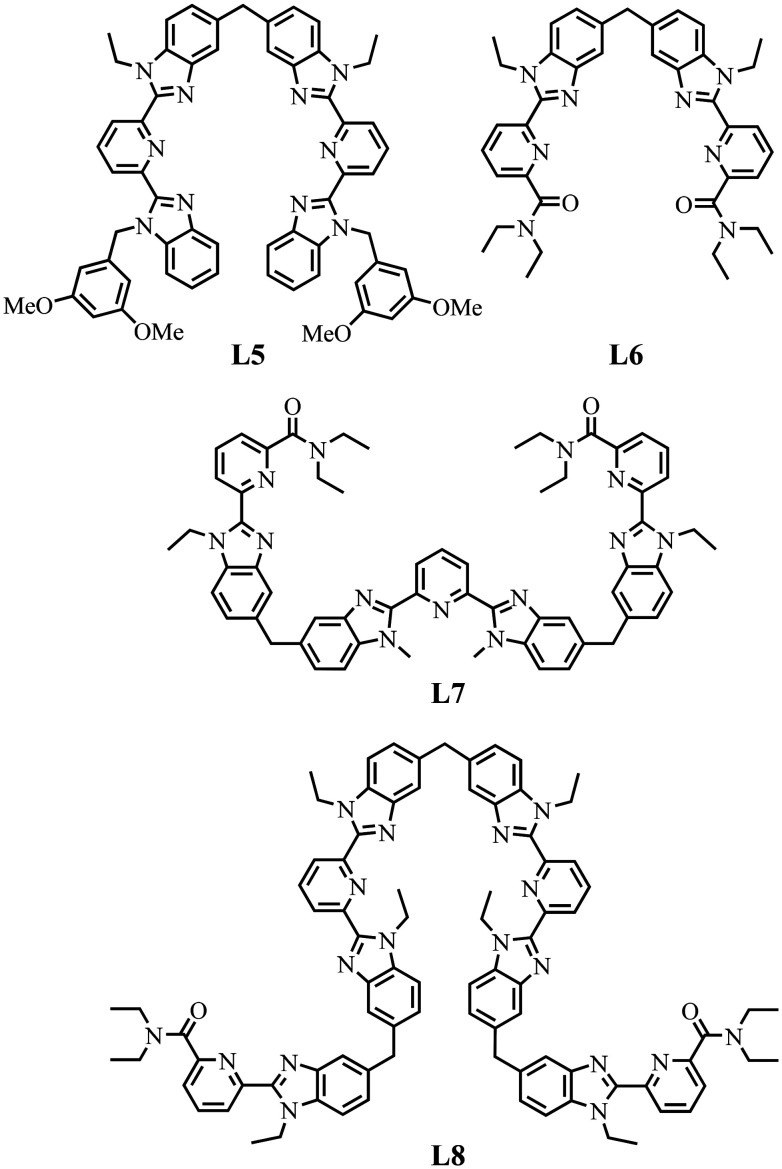
Chemical structures of segmental ligands used for the self-assemblies of heterometallic dinuclear [(L5_3_)(Ln^A^)_(2−*n*)_(Ln^B^)_*n*_]^6+^ (*n* = 0, 1, 2)^65^ and [(L6_3_)(Ln^A^)_(2−*n*)_(Ln^B^)_*n*_]^6+^ (*n* = 0, 1, 2),^66,83^ trinuclear [(L7_3_)(Ln^A^)_(3−*n*)_(Ln^B^)_*n*_]^9+^ (*n* = 0, 1, 2, 3)^11^ and tetranuclear [(L8_3_) (Ln^A^)_(4−*n*)_(Ln^B^)_*n*_]^12+^ (*n* = 0, 1, 2, 3, and 4)^12^ helicates.

The origin of the latter driving force is far from being obvious, but it can be traced back to related trends observed for the thermodynamic self-assemblies of symmetrical dinuclear [(L5_3_)(Ln^A^)_(2−*n*)_(Ln^B^)_*n*_]^6+^ (*n* = 0, 1, 2)^65^ and [(L6_3_)(Ln^A^)_(2−*n*)_(Ln^B^)_*n*_]^6+^ (*n* = 0, 1, and 2),^66,83^ trinuclear [(L7_3_)(Ln^A^)_(3−*n*)_(Ln^B^)_*n*_]^9+^ (*n* = 0, 1, 2, 3)^11^ and tetranuclear [(L8_3_) (Ln^A^)_(3−*n*)_(Ln^B^)_*n*_]^12+^ (*n* = 0, 1, 2, 3, and 4)^12^ helicates (Fig. S29†) based on the segmental ligands L5–L8 (Scheme 5, see Appendices 3–4 for a comprehensive thermodynamic analysis, see ESI).

The two adjacent N_6_O_3_ binding units found in [(L6_3_)(Ln^A^)_(2−*n*)_(Ln^B^)_*n*_]^6+^ are not able to induce deviations from statistical mixtures in solution (Fig. A3-1a in Appendix 3, see ESI†) and one systematically obtains *u*^mix^_1–2_ = 1 for any lanthanide pairs (Δ*E*^mix^_1–2_ = 0 in Table S29†). In contrast, the two connected N_9_ sites implemented in [(L5_3_)(Ln^A^)_(2−*n*)_(Ln^B^)_*n*_]^6+^ induce positive mixing energies 
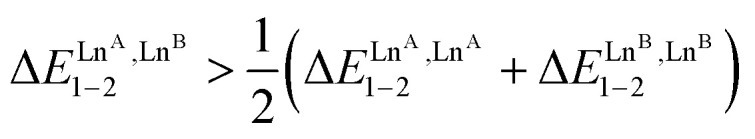
 (Fig. A3-1b and Δ*E*^mix^_1–2_ > 0 in Table S28†), which favor homometallic matching beyond statistical distributions, as long as at least one lanthanide of the metallic pairs belongs to the second half of the lanthanide series. Moving from two adjacent identical nine-coordinated binding sites, as found in [(L5_3_)(Ln^A^)_(2−*n*)_(Ln^B^)_*n*_]^6+^ (N_9_–N_9_) or in [(L6_3_)(Ln^A^)_(2−*n*)_(Ln^B^)_*n*_]^6+^ (N_6_O_3_–N_6_O_3_), toward two different connected N_9_ and N_6_O_3_ sites in *HHH*-[(L4_3_Zn)(Ln^A^)_(2−*n*)_(Ln^B^)_*n*_]^8+^ brings a novel dimension to the size discriminating process. Firstly, due to the presence of the central constrained N_9_ site,^84^ the mixing rule Δ*E*_1–2_^mix^ > 0 discards the formation of the heterolanthanide *HHH*-[(L4_3_Zn)(Ln^A^)(Ln^B^)]^8+^ isomers as soon as the small Lu^3+^ cation is considered as a member of the lanthanide pair in [(La)(Lu)] and [(Eu)(Lu)]. Secondly, the non-zero permutation energies provided by the existence of the two different binding sites (N_9_–N_6_O_3_) may partially compensate for the latter detrimental effect, and the formation of the heterolanthanide *HHH*-[(L4_3_Zn)(Ln^A^)Lu]^8+^ isomers still represents 40–50% of the speciation in solution (Table 1, entries 4 and 5). Finally, when two large lanthanides are bound in *HHH*-[(L4_3_Zn)LaEu]^8+^, both the balance of the intermetallic interactions (Δ*E*^mix^_1–2_ < 0) and site selectivity 
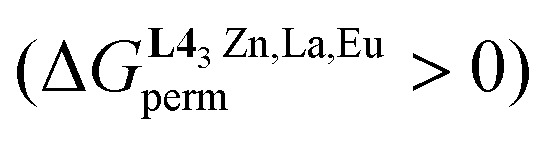
 contribute favorably and synergistically to a large deviation of statistics with the formation of up to 70% of the heterolanthanide *HHH*-[(L4_3_Zn)LaEu]^8+^ and *HHH*-[(L4_3_Zn)EuLa]^8+^ in solution, which exist moreover in a |[(La)(Eu)]|/|[(Eu)(La)]| = 9 : 1 ratio (Table 1 and Fig. 5).

## Conclusions

Puzzled by preliminary, partial and explorative studies reported for [(L1)_3_Ln^A^Ln^B^(NO_3_)(H_2_O)(pyridine)] (Scheme 1),^36^ [(L2)_3_(Ln^A^)_(2−*n*)_(Ln^B^)_*n*_]^6+^ (Scheme 2)^10^ and [(L3)LaLu]^6+^ (Scheme 3),^52^ which claimed for some selective lanthanide recognition to form f–f′ complexes under thermodynamic control in solution, one realizes that any pertinent discriminating effects, if they exist, should be quantitatively addressed with the help of two simple free energy descriptors measuring (*i*) the intermolecular affinity of a given preorganized binding site *i* for the entering lanthanide Ln^*j*^ (Δ*G*^Ln*j*^_aff, *i*_ = −*RT* ln(*f*^Ln*j*^_*i*_)) and (ii) the balance of the intermetallic interactions operating within adjacent pairs of lanthanides 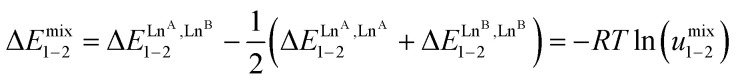.^56^ Although a direct access to these two crucial thermodynamic descriptors proved to be (very) difficult,^80^ an indirect approach appears to be possible since the experimentally accessible permutation equilibrium (eqn (7)), which accompanies the distribution of the various heterolanthanide isomers, and the exchange equilibrium (eqn (8)), which measures the amounts of homo- *versus* heterolanthanide complexes formed, reflect these thermodynamic parameters in solution. With this in mind, the lack of reliable and complete speciations addressed for the non-symmetrical [(L1)_3_Ln^A^Ln^B^(NO_3_)(H_2_O)(pyridine)],^36^ [(L2)_3_(Ln^A^)_(2−*n*)_(Ln^B^)_*n*_]^6+^ (Table 2, entry 1)^10^ and [(L3)LaLu]^6+^ (Table 2, entry 2)^52^ complexes limits further rational thermodynamic analysis. In contrast, the detailed solution studies reported for the symmetrical dinuclear triple-stranded [(L5_3_)(Ln^A^)_(2−*n*)_(Ln^B^)_*n*_]^6+^ and [(L6_3_)(Ln^A^)_(2−*n*)_(Ln^B^)_*n*_]^6+^ helicates in solution can be used to initiate the thermodynamic exploration.^80^ The strict statistical behavior observed for the loading of pairs of lanthanide cations in [(L6_3_)(Ln^A^)_(2−*n*)_(Ln^B^)_*n*_]^6+^ indicates that the sequence of two adjacent semi-flexible N_6_O_3_ binding sites is not able to induce any significant recognition along the lanthanide series (Table 2, entry 3). The same behaviour characterizes the binding of the two large La^3+^ and Eu^3+^ cations in [(L5_3_)(La)_(2−*n*)_(Eu)_*n*_]^6+^ (Table 2, entry 4). However, when at least one lanthanide of the pairs is smaller than Gd^3+^, the two adjacent N_9_ binding sites in the latter complexes [(L5_3_)(Ln^A^)_(2−*n*)_(Ln^A^)_*n*_]^6+^ show a global preference for the formation of homometallic complexes due to cooperative intermetallic mixing energies Δ*E*^mix^_1–2_ ≈ 2 kJ mol^−1^ (Table 2, entry 5). The symmetrical trinuclear [(L7_3_)(Ln^A^)_(3−*n*)_(Ln^B^)_*n*_]^9+^ (N_6_O_3_–N_9_–N_6_O_3_, Table 2 entry 6) and tetranuclear [(L8_3_)(Ln^A^)_(4−*n*)_(Ln^B^)_*n*_]^12+^ (N_6_O_3_–N_9_–N_9_–N_6_O_3_, Table 2 entry 7) helicates confirm these trends with the appearance of sizeable unbalanced intermetallic interactions (*i.e.* Δ*E*^mix^_1–2_ ≠ 0) only when a sequence of two adjacent N_9_ site binding sites exists.

**Table 2:** Summary of the thermodynamic free energy changes relevant to address the difference in intermolecular affinity and in intermetallic interactions which control the speciation of f–f′ helicates under thermodynamic control in solution beyond statistical distributions mentioned in columns 4 and 7

Helicate	Binding sites	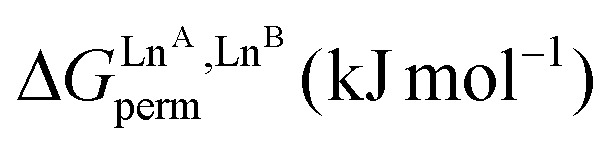	Δ*G*^stat^_perm_ (kJ mol^−1^)	Δ*E*^mix^_1–2_ (kJ mol^−1^)	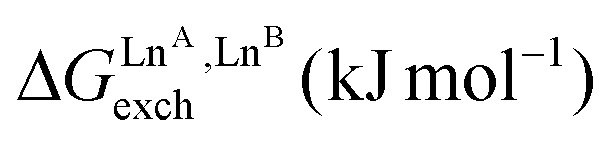	Δ*G*^stat^_exch_ (kJ mol^−1^)	Condition	Favoured species^a^	Ref.
[(L2)_3_(Ln^A^)_(2−*n*)_(Ln^B^)_*n*_]^6+^	N_9_–N_6_O_3_/N_8_–N_7_O_2 _b	—	0	—	−14.1 to −6.4	−3.4	—	Hetero	10
[(L3)_3_(La)_(2−*n*)_(Lu)_*n*_]^6+^	R–N_9_–N_6_O_3 _c	12.1	0	0^d^	—	−3.4	—	—	52
[(L6)_3_(Ln^A^)_(2−*n*)_(Ln^B^)_*n*_]^6+^	N_6_O_3_–N_6_O_3_	0	0	−0.6 to 0.4	−3.5 to −3.3	−3.4	—	Statistical	83
[(L5)_3_(Ln^A^)_(2−*n*)_(Ln^B^)_*n*_]^6+^	N_9_–N_9_	0	0	0	−3.4	−3.4	*R* ^LnA^ ≥ *R*^Gd^ and *R*^LnB^ ≥ *R*^Gd^	Statistical	65
[(L5)_3_(Ln^A^)_(2−*n*)_(Ln^B^)_*n*_]^6+^	N_9_–N_9_	0	0	1.6 to 2.3	−0.2 to 1.2	−3.4	*R* ^LnA^ < *R*^Gd^ or *R*^LnB^ < *R*^Gd^	Homo	65
[(L7)_3_(Ln^A^)_(3−*n*)_(Ln^B^)_*n*_]^9+^	N_6_O_3_–N_9_–N_6_O_3_	2.2 to 4.2	3.34	−0.6 to 0.2	−10.8 to −5.4	−5.4	—	Hetero	67
[(L8)_3_(La)_(4−*n*)_(Lu)_*n*_]^12+^	N_6_O_3_–N_9_–N_9_–N_6_O_3_	—	—	−2	−47.5	−22.2	—	Hetero	12
[(L4_3_Zn)(Ln^A^)_(2−*n*)_(Ln^B^)_*n*_]^8+^	[ZnN_6_]–N_9_–N_6_O_3_	3.0 to 5.4	0	1.2 to 1.9	−3.6 to −0.4	−3.4	*R* ^LnA^ < *R*^Gd^ or *R*^LnB^ < *R*^Gd^	Homo	This work
[(L4_3_Zn)(Ln^A^)_(2−*n*)_(Ln^B^)_*n*_]^8+^	[ZnN_6_]–N_9_–N_6_O_3_	5.4	0	−0.8	−7.5	−3.4	*R* ^LnA^ ≥ *R*^Gd^ and *R*^LnB^ ≥ *R*^Gd^	Hetero	This work

a Homo resp. hetero = preference for homometallic, resp. heterometallic lanthanide complexes; statistical = no preference.

b The triple-stranded helicates exist as variable mixtures of *HHH* (N_9_–N_6_O_3_) and *HHT* (N_8_–N_7_O_2_) isomers.

c R represents an organic tripod.

d The mixing energy is arbitrarily fixed to Δ*E*^mix^_1–2_ = 0.

When a constrained sequence of two adjacent N_6_O_3_ and N_9_ binding sites is ensured *via* the connection of the ligand strands to a covalent sulfur tripod in [(L3)LaLu]^6+^ (Table 2, entry 2) or to a non-covalent [ZnN_6_] podand in the *HHH*-[(L4_3_Zn)(Ln^A^)_(2−*n*)_(Ln^B^)_*n*_]^8+^ helicates (Table 2, entries 8 and 9), both the specific binding site affinities (*via*
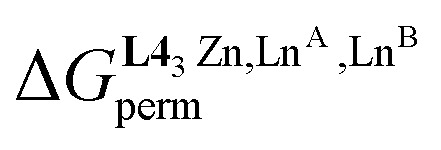
, eqn (7)) and the intermetallic mixing energies (*via*
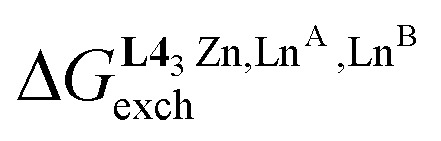
, eqn (8)) can be exploited for boosting the formation of one targeted heterolanthanide isomer in solution. The systematic preference of the central N_9_ site for binding the largest lanthanide of the Ln^A ^: Ln^B^ pair favors the formation of the heterolanthanide *HHH*-[(L4_3_Zn)Ln^A^Ln^B^]^8+^ isomer when *R*^LnA^ > *R*^LnB^
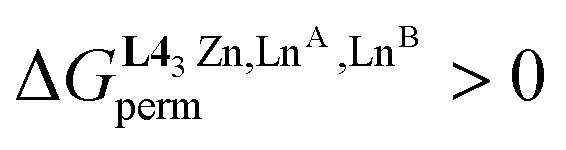
. However, the unfavorable mixing energy 
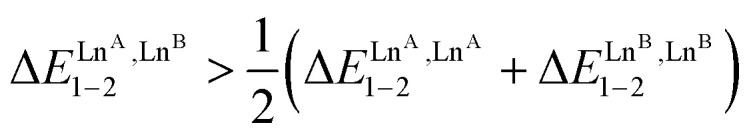
 accompanying the distribution of the two lanthanides within the two sites as soon as one is smaller than Gd^3+^ limits this drift with the formation of only 51% of the heterolanthanide complexes for the La : Lu pair and 35% for the Eu : Lu pair (Table 2, entry 8). The latter restriction is removed when the two lanthanides belong to the first half of the series as demonstrated for the challenging La^3+ ^: Eu^3+^ ionic pair in *HHH*-[(L4_3_Zn)LaEu]^8+^ (Table 2, entry 9; the sizes of the two cations differ by only 8%), where the latter isomer accounts for 63% of the speciation in solution under stoichiometric conditions (|L4_3_Zn|_tot_ = |La|_tot_ = |Eu|_tot_). This largely exceeds the 25% predicted by the statistical distribution. The road to the selective formation of f–f′ heterometallic complexes under thermodynamic control is still a long one, but the use of non-covalent tripods for programming specific sequences of binding sites, as demonstrated here for *HHH*-[(L4_3_Zn)Ln^A^Ln^B^]^8+^ helicates, corresponds to a major step forward in the rational design of heterolanthanide complexes obtained under thermodynamic control.

## Conflicts of interest

There are no conflicts to declare.

## Supplementary Material

DT-053-D4DT00610K-s001

DT-053-D4DT00610K-s002
