# Supplementary material for: Programming heterometallic 4f–4f′ helicates under thermodynamic control: the circle is complete
Source: Dalton Trans. 2024 Mar 1;53(13):6050–62. doi: 10.1039/d4dt00610k (PMC10964198; doi:10.1039/d4dt00610k)
Supplement: DT-053-D4DT00610K-s001 [file DT-053-D4DT00610K-s001.pdf]

Electronic Supplementary Material (ESI) for Dalton Transactions.
This journal is © The Royal Society of Chemistry 2024

Programming heterometallic 4f-4f' helicates under thermodynamic control: the circle is complete†

Charlotte Egger, Laure Guénée, Neel Deorukhkar, and Claude Piguet*

Supporting Information

(89 pages)

Appendix 1: Broadening of the introductory discussion for scientific completeness.

The successful implementation of pure heterometallic f-f' molecular complexes in solution thus relies on multistep strategies which exploit the remarkable kinetic inertness provided by the complexation of Ln^{3+} within rigid and highly preorganized receptors such as macrobicyclic cryptands (Fig. A1-1a)[15] or with less preorganized but polyanionic multidentate receptors as found in porphyrins (Fig. A1-1b),[16] phthalocyanines[17] or tetraxetan (DOTA) derivatives (Fig. A1-1c).[18-20]

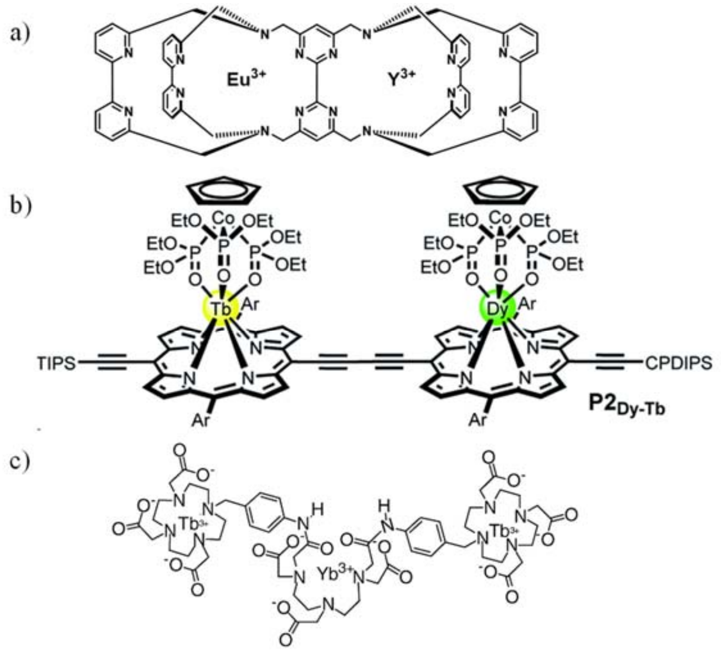

Fig. A1-1 Chemical structures of heterometallic lanthanide complexes synthesized under kinetic control via a) metallic insertion into an inert di-cryptand,[15] b) covalent connection of preformed lanthanide-containing porphyrin monomers[26] and c) formation of a secondary binding site from two inert monometallic macrocyclic lanthanide units.[18]

In the intervening years, this kinetic-based methodology has been further exploited in a number of closely related strategies including (i) selective metalation/demetalation sequences,[21] (ii) stepwise deprotection/activation of coordination sites[22] and/or (iii) creation of covalent bonds between preformed monometallic complexes (Fig. A1-1c).[23-26] These approaches provide a complete control of the metal-coordinating site and they guarantee concentration-independent speciations in solution that remain unchanged for extended periods of times (dissociation constants below 10^{-6} s^{-1}), which

makes these complexes well-suited for acting as sensors and probes in biological and biomedical applications.[20]

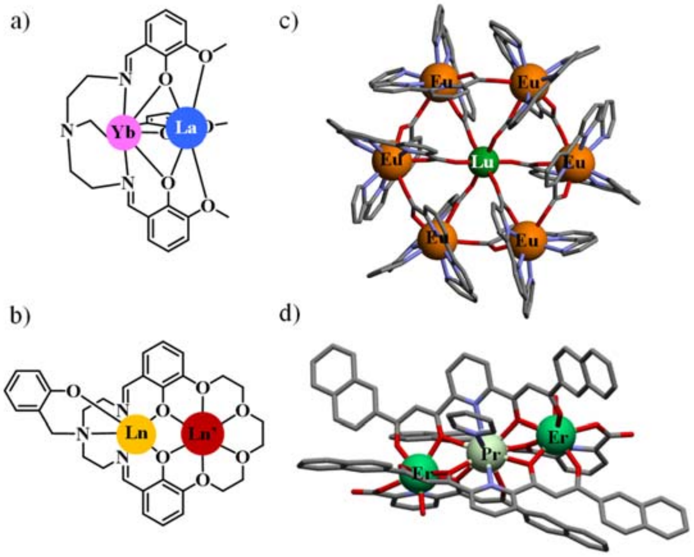

Fig. A1-2 Chemical structures of a) a tripod multisite ligand[32] and b) a compartmental macrocycle[33] engineered for the formation of pure heterodimetallic f-f' complexes in the solid state. Crystal structures of c) a heteropolymetallic f-f' lanthanide wheel[34] and d) a heterometallic f-f' trinuclear lanthanide Q-bit.[38-40]

By broadening the perspective, one easily realizes that both global kinetic inertness and thermodynamic stability may benefit from crystallization processes because long-range stacking interactions may significantly tune the energies of both transition states (kinetics) and/or ground states (kinetics and thermodynamics).[27-29] The preparation of pure, usually serendipitous f-f' assemblies thus often resorts to the preparation of crystalline solids,[30,31-40] among which the pioneer work of Costes and co-workers, who used a tripodal multisite ligand with a small N_4O_3 inner coordination site and a larger O_6 outer site to generate a pure LaYb complex via selective crystallization, merits to be highlighted (Fig. A1-2a).[32] The claimed selective lanthanide loading of non-symmetrical [1+1] compartmental macrocycles by Tamburini and coworkers (Fig. A1-2b)[33] follows the same strategy, while the assembly of a hexameric heteropolymetallic lanthanide wheel by Mazzanti and coworkers highlights the virtues of diverging multidentate ligands in the preparation of f-f' architectures (Fig. A1-2c).[34]

Appendix 2: Experimental Section and full supporting data.

Solvents and starting materials

Chemicals were purchased from Sigma-Aldrich, TCI and Acros and used without further purification unless otherwise stated. Starting synthons **2c**,[58] **3d**[59] and **8**[59] were prepared according to literature procedures. Dichloromethane was dried by distillation over calcium hydride under nitrogen atmosphere. Silica-gel plates (Merck, 60 F_{254}) and aluminium oxide plates (Merck, 60 F_{254}) were used for thin-layer chromatography, SilicaFlash® silica gel P60 (0.04-0.063 mm) and Acros neutral activated alumina (Brockmann I, 0.050-0.200 mm) were used for preparative column chromatography.

Spectroscopic and analytical measurements

^{1}H and ^{13}C NMR spectra were recorded at 298 K on a Bruker Avance 400 MHz spectrometer. Chemical shifts were given in ppm with respect to TMS. All signals were internally referenced to the solvent residue. Due to the lack of solubility of **L4** in CD_3CN, 1:2 mixtures of $CDCl_3$/CD_3CN were used, and all signals were internally referenced to CD_3CN residue. Pneumatically-assisted electrospray (ESI-MS) mass spectra were recorded from on an Applied Biosystems API 150EX LC/MS System equipped with a Turbo Ionspray source. Elemental analyses were performed by K. L. Paglia from the Microchemical Laboratory of the University of Geneva.

X-ray crystallography

Summary of crystal data, intensity measurements and structure refinements for ligand **L4**·C_3H_8O and for its complexes [(**L4**$_3$Zn)Eu_2]$(CF_3SO_3)_8$·12(C_3H_8O), [(**L4**$_3$Zn)La_2]$(CF_3SO_3)_8$·7.25(CH_3CN) are collected in Tables S1 and S8-S24 with pertinent bond lengths, bond angles and interplanar angles. ORTEP views with pertinent numbering schemes are gathered in Fig.s S3, S21 and S24. The crystals were mounted on Hampton cryoloops with protection oil. X-ray data collections were performed with a XtaLAB Synergy-S diffractometer equipped with a hybrid pixel array "hypix arc 150" detector. The structures were solved by using dual space methods (SHELXT).[A2-1] Full-matrix least-square refinements on F^2 were performed with SHELXL.[A2-1] CCDC 2320733-2320735 contain the supplementary crystallographic data for this paper. These data can be obtained free of charge from The Cambridge Crystallographic Data Centre via https://www.ccdc.cam.ac.uk/structures/.

The ligand **L4** crystallized in the monoclinic space group *C2/c* with two disordered isopropanol molecules included in the asymmetric cell unit. Both solvent molecules are located around a 2-fold symmetry axis and their total occupancies were fixed to 0.5 each, giving one C_3H_8O in the chemical formula moiety.

Due to disorder in the crystal structure of [(**L4**$_3$Zn)Eu_2]$(CF_3SO_3)_8$·12(C_3H_8O) (counter ions and isopropanol solvent molecules), the diffraction at high angle was poor, and data were truncated, thus

limiting their resolution and their completeness. A solvent mask was calculated, and 1007 electrons were found in a volume of 3947 A^3 in 3 voids per unit cell. This is consistent with the presence of 1 triflate anion and 12 isopropanol per formula unit, which accounts for 962 electrons per unit cell. These "squeezed" triflate and solvent molecules were added in the chemical formula moiety but not in the total chemical formula sum.

The crystal structure of $[(\mathbf{L4}_3Zn)La_2](CF_3SO_3)_8\cdot 7.25(CH_3CN)$ showed high disorder of counter ions (triflate) and acetonitrile solvent molecules that are located in voids, and some of them on symmetry elements. Constrains on occupancies, ADPs, and distances were used to refine these disordered molecules.

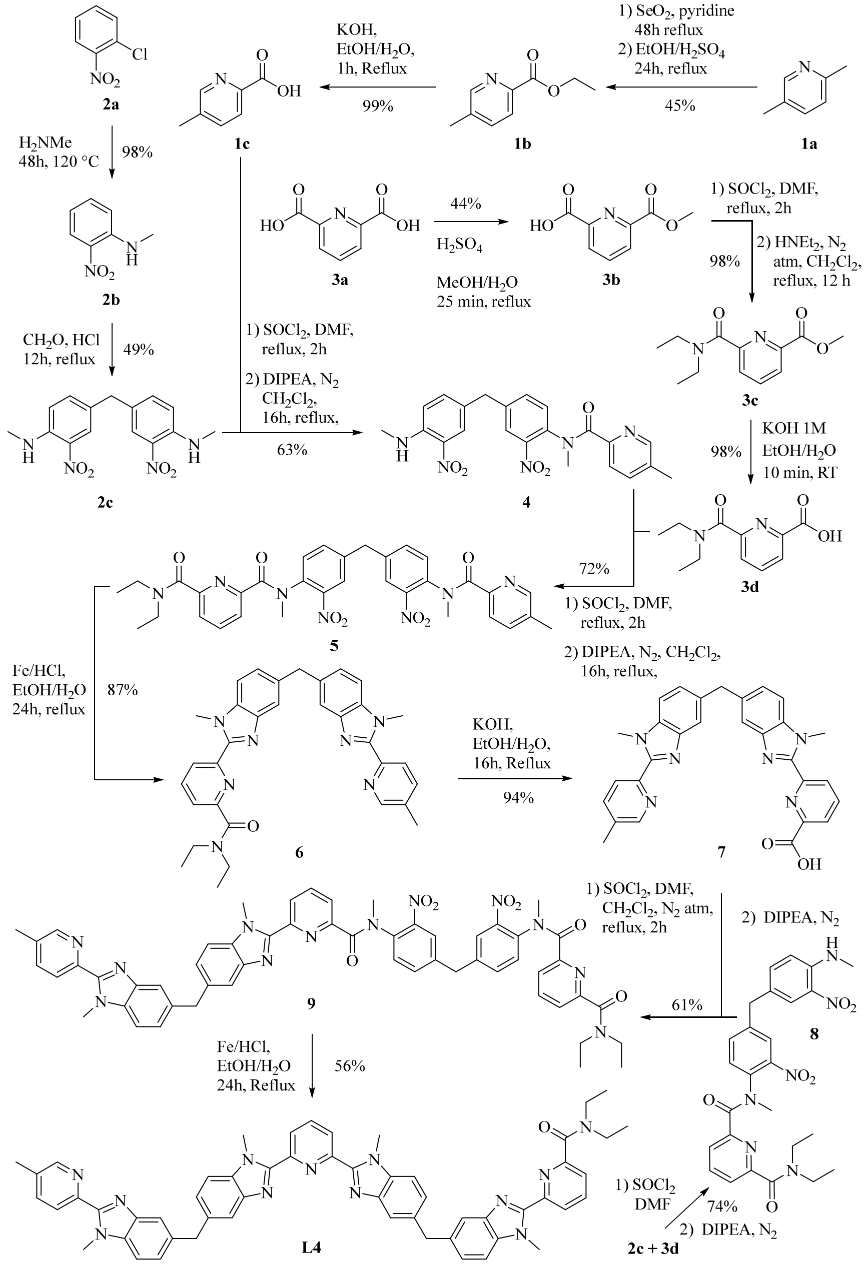

Fig. A2-1 Synthesis of the segmental ligand **L4**.

Synthesis of ethyl-5-methylpicolinate (1b).

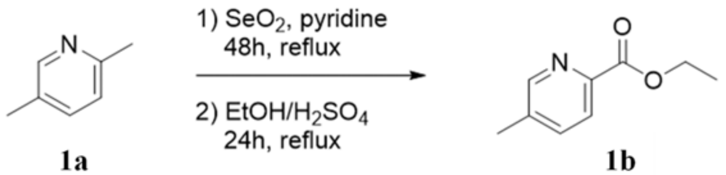

In a 250 mL flask were successively added selenium dioxide (15.5 g, 140 mmol, 1.5 eq), 2,5-lutidine (**1a**,10.8 mL, 93.3 mmol, 1.0 eq), and 60 mL of pyridine. The mixture was refluxed for 48 hours. The pyridine was evaporated under high vacuum at room temperature, and the brown residue was redissolved in about 450 mL of ethanol. Concentrated sulfuric acid (8.0 mL, 149 mmol, 1.6 eq) was added and the mixture was refluxed for 24 hours. 50 mL of distilled water were added, and the pH was raised to 7 by addition of a 5 M aqueous solution of sodium hydroxide. The ethanol was removed under reduced pressure and the remaining aqueous phase was extracted 4 times with 100 mL of dichloromethane. The combined black/dark green organic phases were dried over anhydrous sodium sulfate, filtered, and concentrated under reduced pressure. The resulting black oil was purified by distillation under high vacuum (T_{vap} = 65 °C at $7.8.10^{-2}$ mbar) to yield a colorless oil (6.89 g, 41.7 mmol, yield 45%).

ESI-MS (soft positive, $CHCl_3/CH_3OH/HCO_2H$ 49.95:49.95:0.1) *m/z*: $[M+H]^+$ 166.0

^{1}H-NMR (400 MHz, $CDCl_3$, 298 K) δ (ppm): 8.57 (d, ^4J = 1.6 Hz, 1H), 8.03 (d, ^3J = 7.9 Hz, 1H), 7.62 (dd, ^3J = 8.0 Hz, ^4J = 1.5 Hz, 1H), 4.46 (q, ^3J = 7.1 Hz, 2H), 2.41 (s, 3H), 1.43 (t, ^3J = 7.1 Hz, 3H).

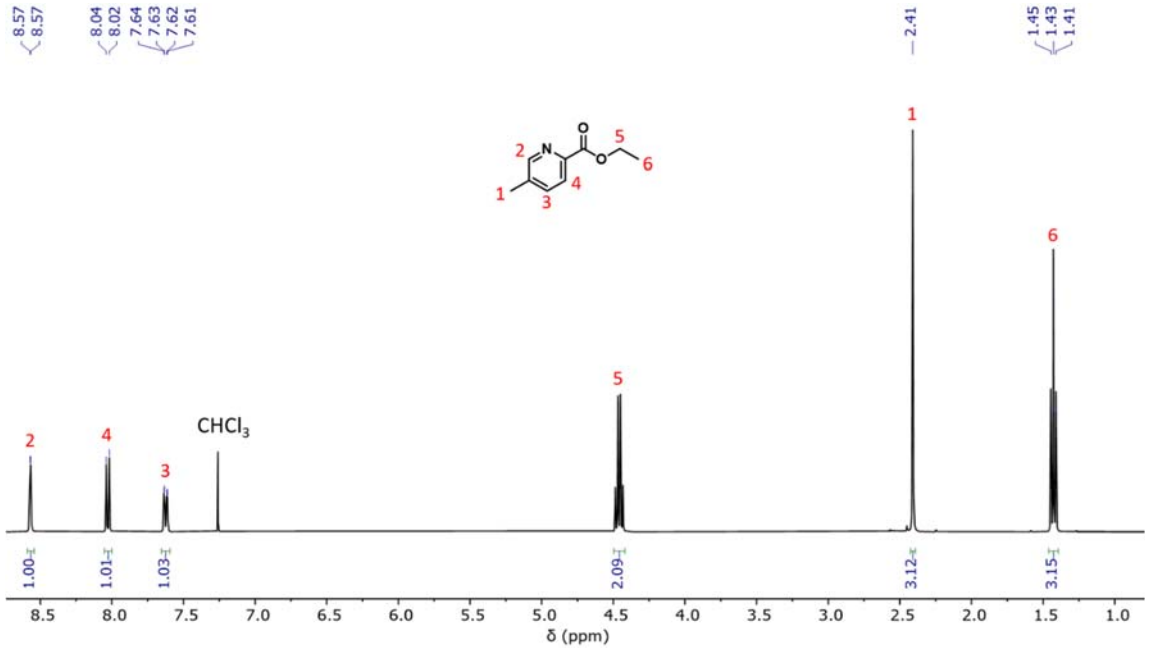

Fig. A2-2 ^{1}H-NMR spectrum of ethyl-5-methylpicolinate (**1b**).

Synthesis of 5-methylpicolinic acid (1c).

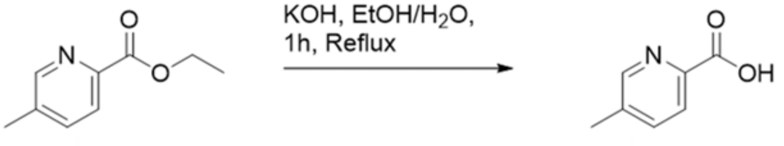

1b **1c**

1b (5.00 g, 30.3 mmol, 1.0 eq) was diluted in a 1:1 mixture of ethanol/water (92:92 mL). Potassium hydroxide (5.60 g, 100 mmol, 3.3 eq) was added and the mixture was refluxed for 1 hour. Ethanol was evaporated under reduced pressure. The remaining light yellow/green aqueous phase was extracted three times with 100 mL of dichloromethane, then acidified to pH 3 by addition of concentrated hydrochloric acid. The water was removed under reduced pressure, and the solid residue was solubilized in about 350 mL of acetonitrile. The solution was heated to 70 °C for 3 hours, then filtered while still hot on a Büchner funnel, and finally concentrated under reduced pressure until the product started precipitating. The solution was left in the fridge overnight, and the light brownish crystals were collected by filtration the next morning (4.12 g, 30.1 mmol, yield 99%). The filtrate was heated to 70 °C again and the procedure was repeated at least three times.

ESI-MS ($CH_3CN/H_2O/Et_3N$, soft negative), *m/z*: $[M-H]^-$ 136.0

^{1}H-NMR (400 MHz, $CDCl_3$, 298 K) δ (ppm): 10.62 (s, 1H), 8.56 (s, 1H), 8.15 (d, ^3J = 7.9 Hz, 1H), 7.77 (d, ^3J = 7.9, 1H), 2.48 (s, 3H).

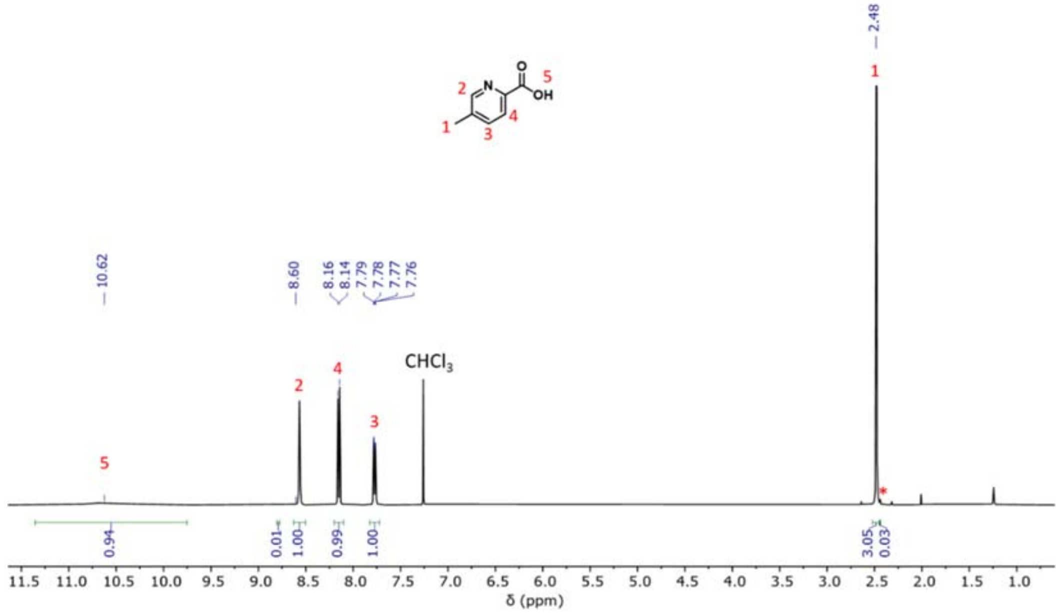

Fig. A2-3 ^{1}H-NMR spectrum of 5-methylpicolinic acid (**1c**). The red star shows the integration of the ^{1}H-NMR signal of the methyl protons of 6-methylnicotinic acid, the isomer of **1c**, which is formed as a minor side product during the oxidation of **1a**. The integration ration between **1c** and its isomer is 100:1.

Synthesis of *N*,5-dimethyl-*N*-(4-(4-(methylamino)-3-nitrobenzyl)-2-nitrophenyl)-picolinamide (4)

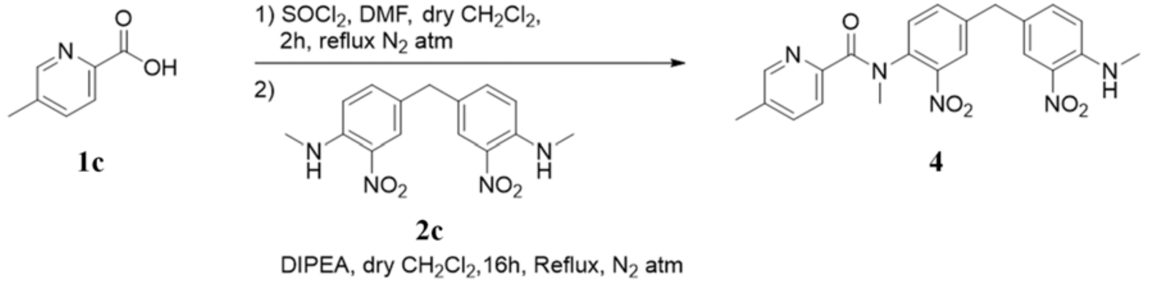

In a flask charged with **1c** (2.00 g, 14.6 mmol, 1.0 eq) and evacuated and backfilled with N_2 three times were added 120 mL of dry dichloromethane. Thionyl chloride (10.6 mL, 146 mmol, 10 eq) was added dropwise, followed by *N*,*N*-dimethylformamide (0.733 mL, 8.75 mmol, 0.6 eq). The mixture was refluxed under N_2 atmosphere for 2 hours. The volatiles were removed under high vacuum, and the solid residue was further dried 30 minutes. Meanwhile, 4,4'-methylenebis(*N*-methyl-2-nitroaniline) (**2c**, 6.92 g, 21.9 mmol, 1.5 eq) was solubilized in about 150 mL of dry dichloromethane. The acyl chloride residue was resuspended in about 80 mL of dry dichloromethane, then added dropwise to the solution of **2c**. *N*,*N*-diisopropylethylamine (11.4 mL, 65.6 mmol, 3 eq) was then added slowly, and the mixture was refluxed overnight under N_2 atmosphere. The crude mixture was concentrated under reduced pressure, then partitioned between 200 mL of dichloromethane and 250 mL of a half-saturated aqueous ammonium chloride solution. The organic phase was separated and the aqueous phase was further extracted three times. The combined organic phases were dried over anhydrous sodium sulfate, filtered, and concentrated under reduced pressure. The crude product was purified by column chromatography on silica gel (SiO_2, CH_2Cl_2/MeOH 99:1) to give **4** as an orange solid (4.00 g, 9.19 mmol, yield 63%).

ESI-MS (soft positive, $CHCl_3/CH_3OH/HCO_2H$ 49.95:49.95:0.1) *m/z*: $[M+H]^+$ 435.8, $[M+Na]^+$ 457.8. ^{1}H-NMR (400 MHz, $CDCl_3$, 298 K) δ (ppm): mixture of rotamers: 8.02-6.83 (m, 9H), 3.93 (s, 2H), 3.47 (s, 3H), 3.03 (s, 3H), 2.25 (s, 3H).

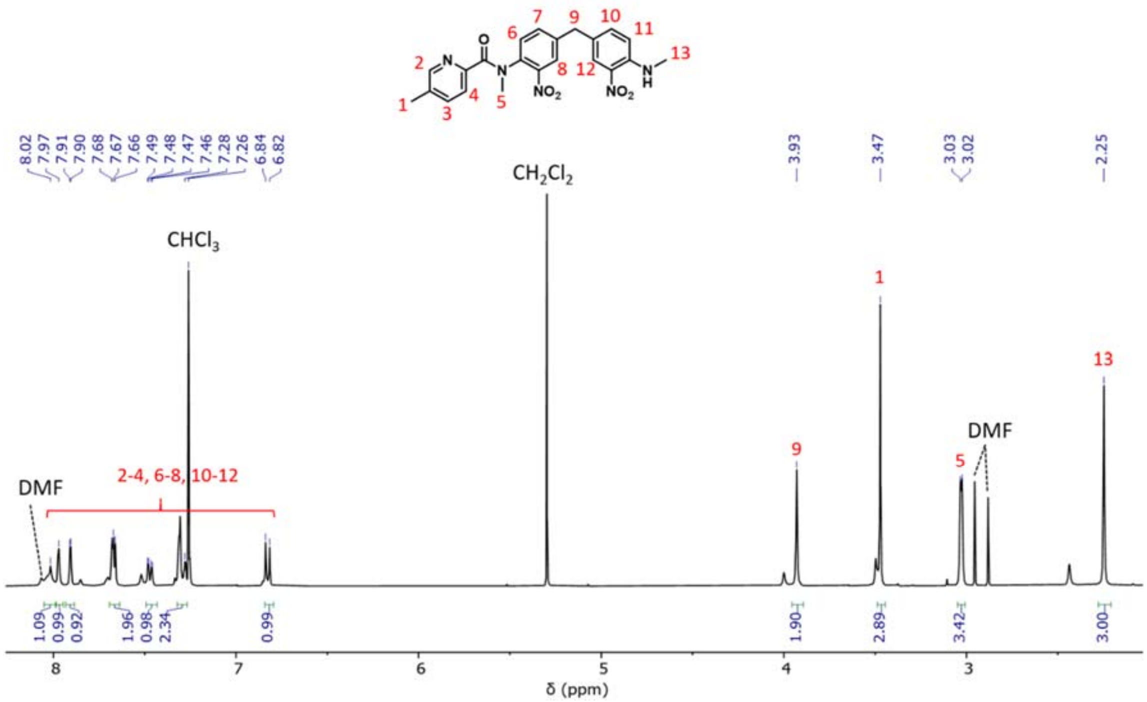

Fig. A2-4 ^{1}H-NMR spectrum of *N*,5-dimethyl-*N*-(4-(4-(methylamino)-3-nitrobenzyl)-2-nitrophenyl)picolinamide (**4**).

Synthesis of *N*[2]-(4-(4-(*N*,5-dimethylpicolinamido)-3-nitrobenzyl)-2-nitrophenyl)-*N*[6],*N*[6]-diethyl-*N*[2]-methylpyridine-2,6-dicarboxamide (5)

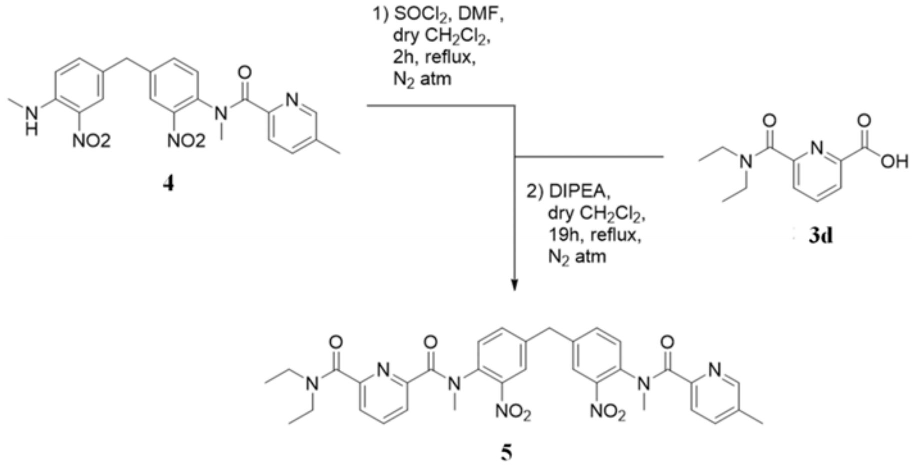

In a flask charged with 6-(diethylcarbamyl)picolinic acid (**3d**, 3.88 g, 17.5 mmol, 2.0 eq) and evacuated and backfilled with N_2 three times were added 120 mL of dry dichloromethane. Thionyl chloride (10.1 mL,175 mmol, 20 eq) was added dropwise, followed by 0.346 mL of *N*,*N*-dimethylformamide (0.439 mL, 5.24 mmol, 0.6 eq). The mixture was refluxed under N_2 atmosphere for 2 hours. The volatiles were removed under high vacuum, and the solid residue was further dried for 30 minutes, then resuspended in about 100 mL of dry dichloromethane. **4** (3.80 g, 8.73 mmol, 1.0 eq) was solubilized in about 50 mL of dry dichloromethane and added dropwise to the acyl chloride

solution. *N*,*N*-diisopropylethylamine (2.28 mL, 10.3 mmol, 1.5 eq) was added slowly, and the mixture was refluxed overnight under N_2 atmosphere. An additional 1.5 eq of base was then added and the mixture was further refluxed for 3 hours under N_2 atmosphere. The crude mixture was concentrated under reduced pressure, then partitioned between 200 mL of dichloromethane and 250 mL of a half-saturated aqueous ammonium chloride solution. The organic phase was separated and the aqueous phase was further extracted three times. The combined organic phases were dried over anhydrous sodium sulfate, filtered, and concentrated under reduced pressure. The crude product was purified by column chromatography on silica gel (SiO_2, CH_2Cl_2/MeOH 98.6:1.4) to give **5** as a beige solid (4.03 g, 6.30 mmol, yield 72%).

ESI-MS (soft positive, $CHCl_3$/CH_3OH/HCO_2H 49.95:49.95:0.1) *m/z*: $[M+H]^+$ 640.3, $[M+NH_4]^+$ 657.3, $[2M+H]^+$ 1279.6.

^{1}H-NMR (400 MHz, $CDCl_3$, 298 K) δ (ppm): mixture of rotamers:

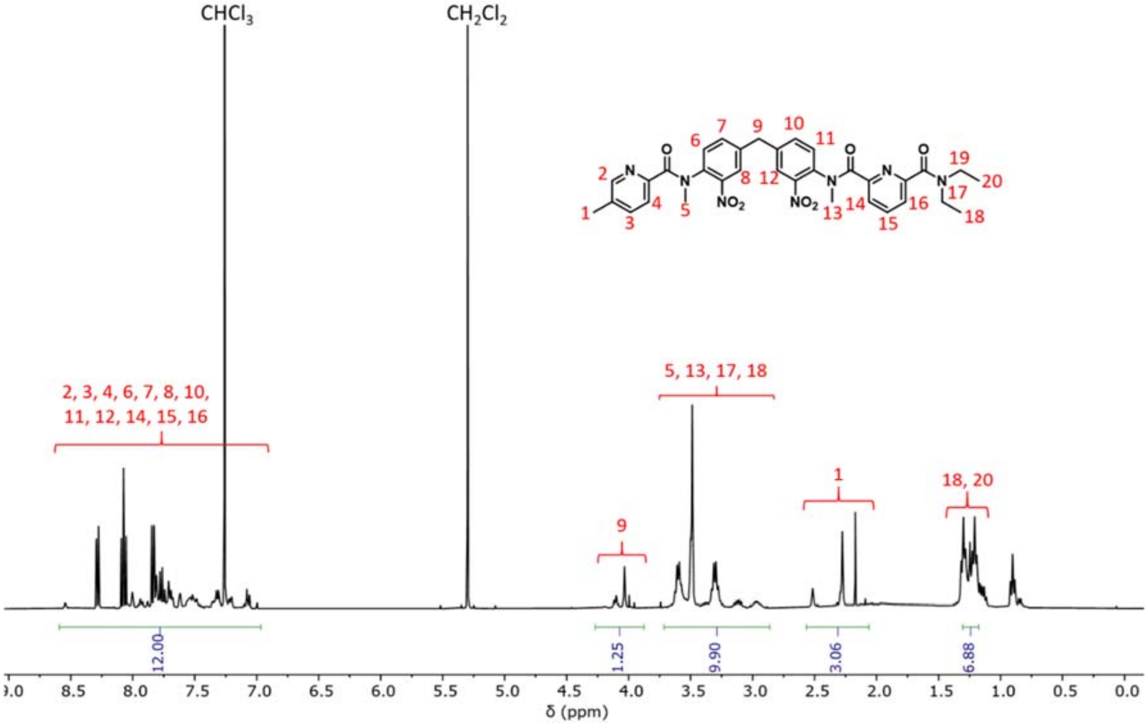

Fig. A2-5 ^{1}H-NMR spectrum of *N*^{2}-(4-(4-(*N*,5-dimethylpicolinamido)-3-nitrobenzyl)-2-nitrophenyl)-*N*^{6},*N*^{6}-diethyl-*N*^{2}-methylpyridine-2,6-dicarboxamide (**5**).

Synthesis of *N*,*N*-diethyl-6-(1-methyl-5-((1-methyl-2-(5-methylpyridin-2-yl)-1*H*-benzo[*d*]imidazol-5-yl)methyl)-1*H*-benzo[*d*]imidazol-2-yl)picolinamide (6).

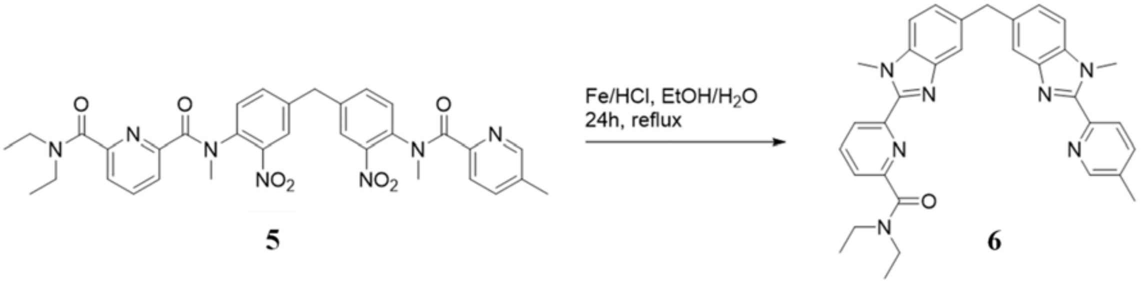

5 (1.42 g, 2.21 mmol, 1.0 eq) was solubilized in a mixture of ethanol/water (427:114 mL). Activated iron (3.72 g, 66.6 mmol, 30.1 eq) and concentrated hydrochloric acid (13.5 mL, 163 mmol, 73.6 eq) were added and the mixture was refluxed for 24 hours under N_2 atmosphere. The ethanol was removed under reduced pressure. 84 mL of distilled water and a solution of 31.9 g of EDTA-disodium salt dihydrate (85.8 mmol, 38.8 eq) in 200 mL of distilled water were added. The pH was raised to 9 by addition of a concentrated aqueous solution of ammonia. 250 mL of dichloromethane and hydrogen peroxide (1.65 mL, 16.1 mmol, 7.3 eq) were added and the biphasic mixture was vigorously stirred for 45 minutes. The organic phase was separated and the aqueous phase was further extracted three times with 200 mL of dichloromethane. The combined organic phases were dried over anhydrous sodium sulfate, filtered, and concentrated under reduced pressure. The crude product was purified by column chromatography over silica gel (SiO_2, CH_2Cl_2/MeOH 98:2) to give **6** as a beige solid (1.05 g, 1.93 mmol, yield 87%).

ESI-MS (soft positive, $CHCl_3$/CH_3OH/HCO_2H 49.95:49.95:0.1) *m/z*: $[M+H]^+$ 544.3, $[2M+H]^+$ 1087.6.

^{1}H-NMR (400 MHz, $CDCl_3$, 298 K) δ (ppm): 8.51 (d, ^4J = 2.3 Hz , 1H), 8.39 (dd, ^3J = 8.1 Hz, ^4J = 1.0 Hz 1H), 8.25 (d, ^3J = 8.1 Hz, 1H), 7.92 (t, ^3J = 7.9 Hz, 1H), 7.70 (d, ^4J = 0.7 Hz, 1H), 7.68 (d, ^4J = 0.7 Hz, 1H), 7.64 (ddd, ^3J = 8.2 Hz, ^4J = 2.3 Hz, ^4J = 0.7 Hz 1H), 7.57 (dd, ^3J = 7.7 Hz, ^4J = 1.1 Hz, 1H), 7.32 (d, ^3J = 8.4 Hz, 1H), 7.31 (d, ^3J = 8.4 Hz, 1H), 7.24 (dd, ^3J = 8.3 Hz, ^4J = 1.6 Hz 1H), 7.20 (dd, ^3J = 8.4 Hz, ^4J = 1.7 Hz 1H), 4.28 (s, 2H), 4.23 (s, 3H), 4.20 (s, 3H), 3.61 (q, ^3J = 7.1 Hz, 2H), 3.36 (t, ^3J = 7.1 Hz, 2H), 2.41 (s, 3H), 1.29 (t, ^3J = 7.2 Hz, 3H), 1.12 (t, ^3J = 7.1 Hz, 3H).

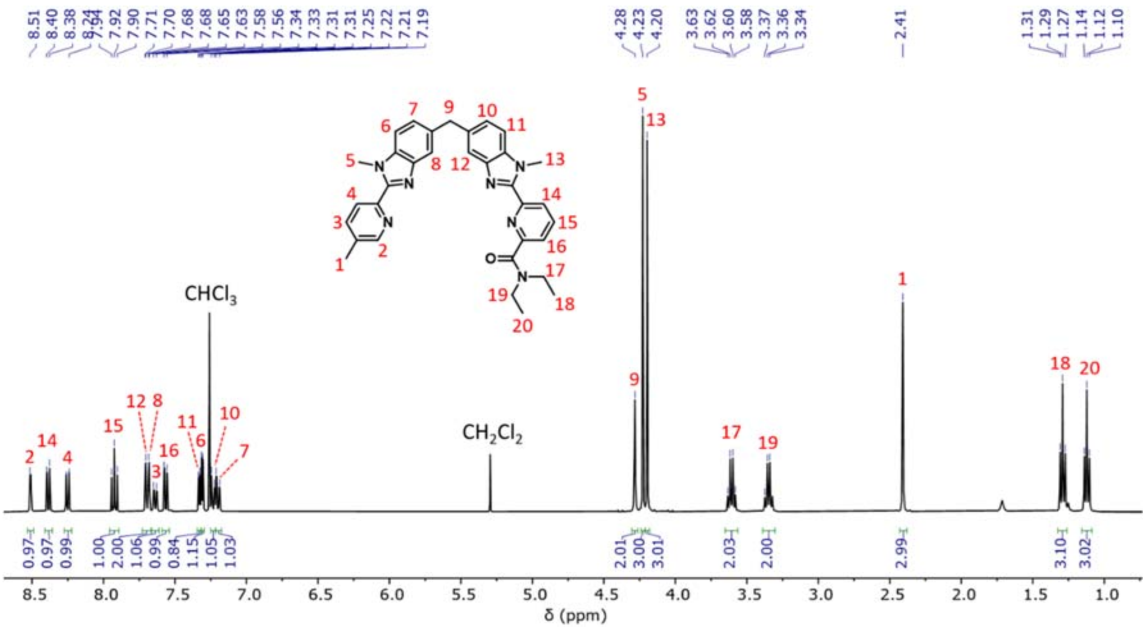

Fig. A2-6 ^{1}H-NMR spectrum of *N*,*N*-diethyl-6-(1-methyl-5-((1-methyl-2-(5-methylpyridin-2-yl)-1*H*-benzo[*d*]imidazol-5-yl)methyl)-1*H*-benzo[*d*]imidazol-2-yl)picolinamide (**6**).

Synthesis of 6-(1-methyl-5-((1-methyl-2-(5-methylpyridin-2-yl)-1*H*-benzo[*d*]imidazol-5-yl)methyl)-1*H*-benzo[*d*]imidazol-2-yl)picolinic acid (7).

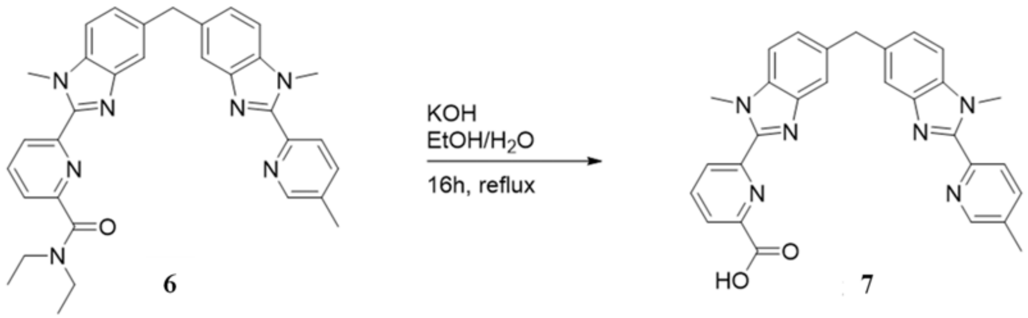

6 (500 mg, 0.920 mmol, 1.0 eq) was solubilized in 125 mL of ethanol and 400 mL of distilled water were added. A solution of 32.25 g of potassium hydroxide (500 mmol, 543 eq) in 100 mL of distilled water was added and the mixture was refluxed for 16 hours. Ethanol was evaporated under reduced pressure. The aqueous phase was then acidified to pH 3 by addition of concentrated hydrochloric acid. The resulting precipitate of **7** was collected by filtration on a Gooch filter, washed with distilled water and ethanol, and dried in the oven (420 mg, 0.860 mmol, yield 94%).

^{1}H-NMR (400 MHz, DMSO-d_6, 298 K) δ 8.70 (s, 1H), 8.52 (d, ^3J = 9.2 Hz, 1H), 8.28 – 8.11 (m, 3H), 7.94 (d, ^3J = 8.2 Hz, 1H), 7.80 (d, ^3J = 8.6 Hz, 1H), 7.74 – 7.63 (m, 3H), 7.46 (d, ^3J = 10.0 Hz, 1H), 7.35 (d, ^3J = 8.6 Hz, 1H), 4.33 (s, 3H), 4.30 (s, 2H), 4.24 (s, 3H), 2.44 (s, 3H).

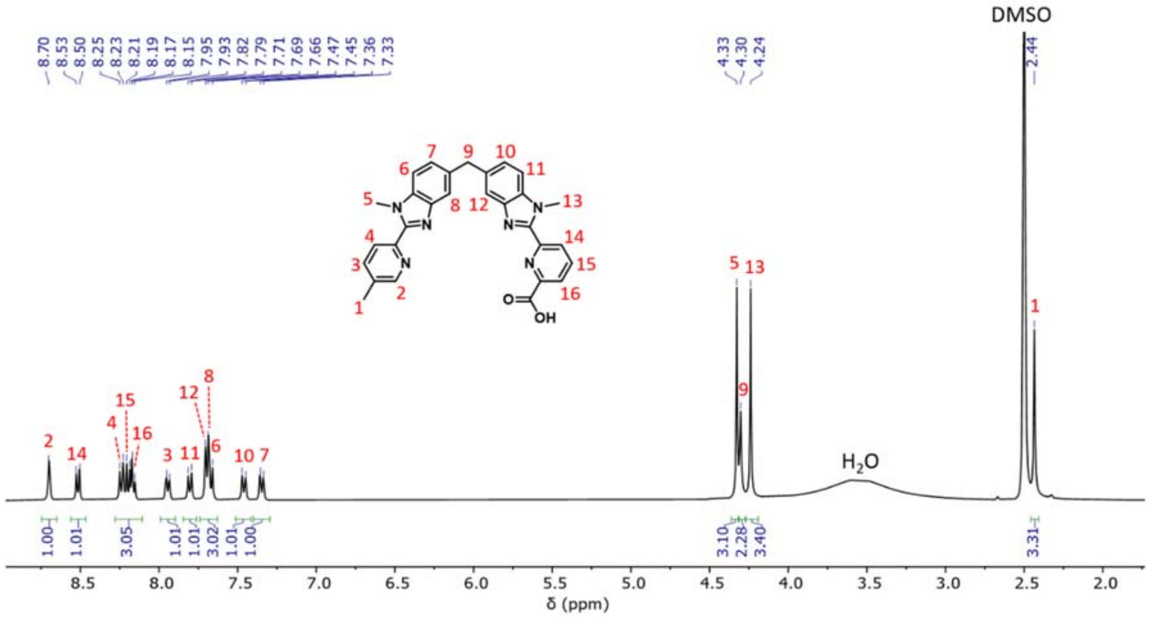

Fig. A2-7 ^{1}H-NMR spectrum of 6-(1-methyl-5-((1-methyl-2-(5-methylpyridin-2-yl)-1*H*-benzo[*d*]imidazol-5-yl)methyl)-1*H*-benzo[*d*]imidazol-2-yl)picolinic acid (**7**).

^{13}C-NMR (101 MHz, DMSO-d_6, 298 K) δ (ppm) : 165.72, 149.96, 148.17, 147.87, 147.80, 143.16, 139.46, 139.14, 138.93, 137.99, 137.01, 136.94, 136.00, 135.14, 133.62, 127.26, 126.21, 125.35, 125.27, 125.00, 118.13, 115.80, 112.16, 111.37, 40.97, 37.44, 33.20, 32.90, 18.05.

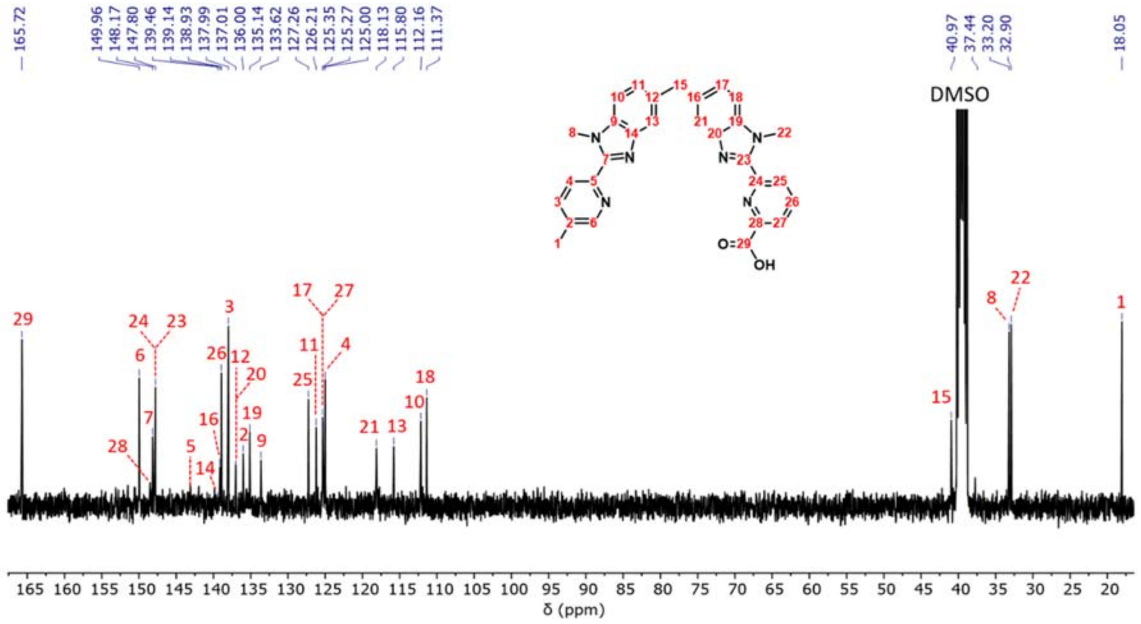

Fig. A2-8 ^{13}C-NMR spectrum of 6-(1-methyl-5-((1-methyl-2-(5-methylpyridin-2-yl)-1*H*-benzo[*d*]imidazol-5-yl)methyl)-1*H*-benzo[*d*]imidazol-2-yl)picolinic acid (**7**).

Synthesis of N^2,N^2-diethyl-N^6-methyl-N^6-(4-(4-(*N*-methyl-6-(1-methyl-5-((1-methyl-2-(5-methylpyridin-2-yl)-1*H*-benzo[*d*]imidazol-5-yl)methyl)-1*H*-benzo[*d*]imidazol-2-yl)picolinamido)-3-nitrobenzyl)-2-nitrophenyl)pyridine-2,6-dicarboxamide (8).

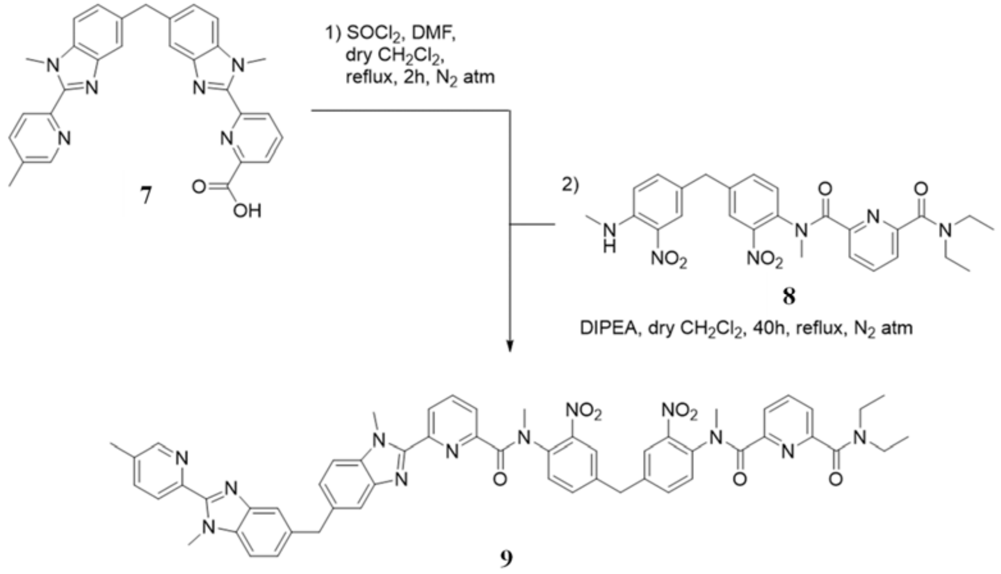

In a 500 mL three-neck flask charged with **7** (0.725 g, 1.48 mmol, 1.0 eq), then evacuated and backfilled with N_2 three times, were added 20 mL of dry dichloromethane. To this suspension were added 16.2 mL of thionyl chloride (223 mmol, 150 eq) dropwise, followed by 0.069 mL (0.890 mmol, 0.6 eq) of *N*,*N*-dimethylformamide. The mixture was refluxed 2 hours under N_2 atmosphere. The volatiles were evaporated under high vacuum, and the solid residue was further dried 1 hour, then resuspended in 20 mL of dry dichloromethane. **8** (1.54 g, 9.82 mmol, 2.0 eq) was solubilized in about 40 mL of dry dichloromethane and the solution was carefully added to the acyl chloride residue. 0.517 mL of *N*,*N*-diisopropylethylamine (2.97 mmol, 2.0 eq) were added, and the resulting mixture was refluxed 16 hours under N_2 atmosphere. In the morning, an additional 0.517 mL of base was added and the mixture was further refluxed 8 hours under N_2 atmosphere. Another 0.253 mL of base were added and the mixture was refluxed overnight. The crude mixture was concentrated under reduced pressure, then partitioned between 150 mL of dichloromethane and 250 mL of a half-saturated aqueous solution of ammonium chloride. The organic phase was separated and the aqueous phase was further extracted three times with 150 mL of dichloromethane. The combined organic phases were dried over anhydrous sodium sulfate Na_2SO_4, filtered, and concentrated under reduced pressure. The crude product was purified by column chromatography on silica gel (SiO_2, CH_2Cl_2/MeOH 97:3) to give **9** as a beige solid (0.894 g, 0.902 mmol, yield 61%).

ESI-MS (soft positive, $CHCl_3$/CH_3OH/HCO_2H 49.95:49.95:0.1) *m*/*z*: $[M+H]^+$ 991.4, $[M+Na]^+$ 1013.3.

^{1}H-NMR (400 MHz, $CDCl_3$, 298 K) δ (ppm): mixture of rotamers: 8.54 – 6.52 (m, 21H), 4.35 – 4.18 (m, 5H), 3.94 – 2.86 (m, 15H), 2.41 (s, 3H), 1.31 – 1.06 (m, 3H), 0.94 – 0.82 (m, 3H).

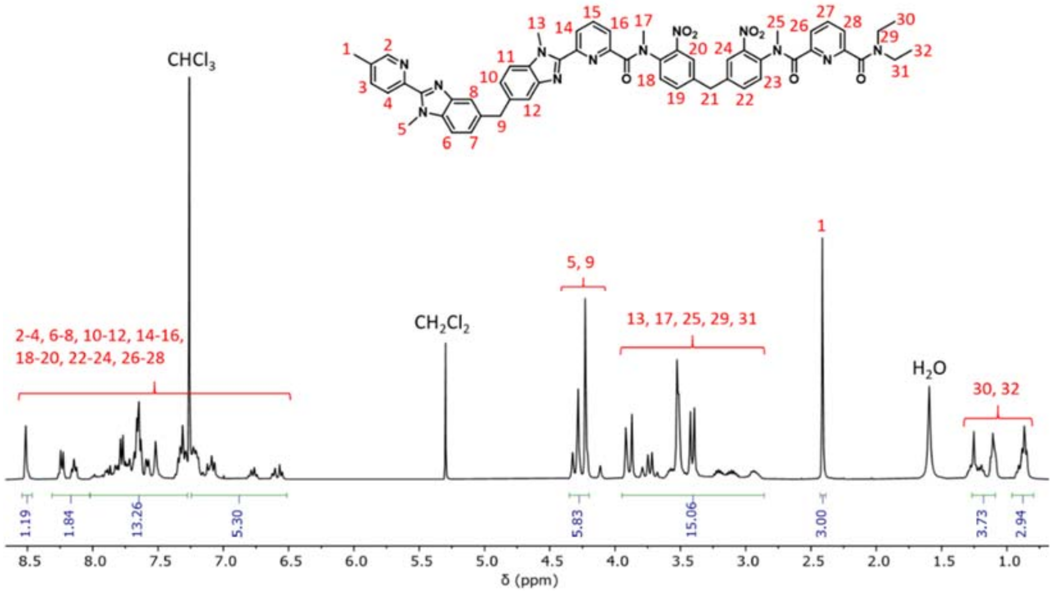

Fig. A2-9 ^{1}H-NMR spectrum of *N*²,*N*²-diethyl-*N*⁶-methyl-*N*⁶-(4-(4-(*N*-methyl-6-(1-methyl-5-((1-methyl-2-(5-methylpyridin-2-yl)-1*H*-benzo[*d*]imidazol-5-yl)methyl)-1*H*-benzo[*d*]imidazol-2-yl)picolinamido)-3-nitrobenzyl)-2-nitrophenyl)pyridine-2,6-dicarboxamide (**9**).

Synthesis of *N*,*N*-diethyl-6-(1-methyl-5-((1-methyl-2-(6-(1-methyl-5-((1-methyl-2-(5-methylpyridin-2-yl)-1*H*-benzo[d]imidazol-5-yl)methyl)-1*H*-benzo[*d*]imidazol-2-yl)pyridin-2-yl)-1*H*-benzo[*d*]imidazol-5-yl)methyl)-1*H*-benzo[*d*]imidazol-2-yl)picolinamide (L4).

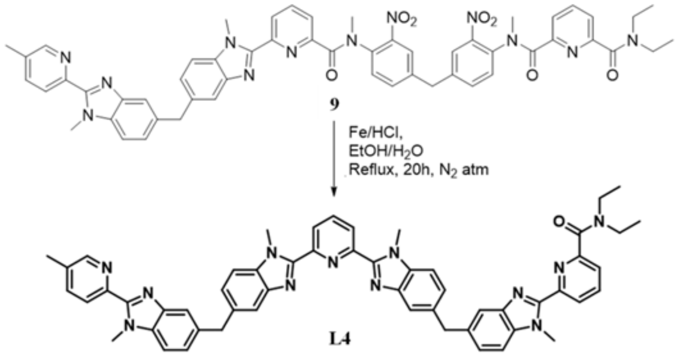

9 (1.84 mmol, 1. eq) was solubilized in a mixture of ethanol/water (545:136 mL) and activated iron (3.08 g, 55.1 mmol, 30 eq) and concentrated hydrochloric acid (11.5 mL, 139 mmol, 75.9 eq) were added. The mixture was refluxed for 20 hours under N_2 atmosphere. Excess iron was removed with a magnet and ethanol was evaporated under reduced pressure. 100 mL of distilled water were added, as well as a solution of 36.7 g of EDTA disodium dihydrate (98.6 mmol, 53.7 eq) in 250 mL of distilled water. The pH was raised to about 8 by addition of concentrated ammonium hydroxide. 250 mL of dichloromethane and 1.7 mL of hydrogen peroxide were added. The biphasic mixture was

stirred vigorously for 2 hours. The organic phase was separated and the aqueous phase was further extracted three times with 200 mL of dichloromethane. The combined organic phases were dried over anhydrous sodium sulfate, filtered, and concentrated under reduced pressure. The crude product was purified by column chromatography on aluminum oxide (Al_2O_3, CH_2Cl_2/MeOH 99:1). The pure product was solubilized in a mixture of dichloromethane and *n*-hexane and evaporated under reduced pressure until **L4** precipitated as a white powder which was collected by filtration (0.920 g, 1.03 mmol, yield 56%). The compound was crystallized by slow diffusion of isopropanol into a 1:1 solution of acetonitrile/dichloromethane at 4°C.

ESI-MS (soft positive, $CHCl_3/CH_3OH/HCO_2H$ 49.95:49.95:0.1) *m/z*: $[M+2H]^{2+}$ 448.4, $[M+H]^+$ 895.5.

Elemental analysis: Calculated for $C_{55}H_{50}N_{12}O{\cdot}0.35CH_2Cl_2$ (%): C 71.89, H 5.53, N 18.17; Found (%): C 72.03, H 5.18, N 17.97.

^{1}H-NMR (400 MHz, CD_2Cl_2, 298 K) δ (ppm): 8.52 (d, ^4J = 2.3 Hz, 1H), 8.42 – 8.35 (m, 3H), 8.23 (d, ^3J = 8.1 Hz, 1H), 8.02 (t, ^3J = 7.9 Hz, 1H), 7.94 (t, ^3J = 7.9 Hz, 1H), 7.69 – 761 (m, 5H), 7.52 (dd, ^3J = 7.7 Hz, ^4J = 1.0 Hz, 1H), 7.41 – 7.33 (m, 4H), 7.29 – 7.20 (m, 4H), 4.28 (s, 2H), 4.27 (s, 2H), 4.22 (s, 3H), 4.22 – 4.20 (m, 9H), 3.57 (q, ^3J = 7.2 Hz, 2H), 3.35 (q, ^3J = 7.2 Hz, 2H), 2.40 (s, 3H), 1.26 (t, ^3J = 7.2 Hz, 3H), 1.11 (t, ^3J = 7.1 Hz, 3H).

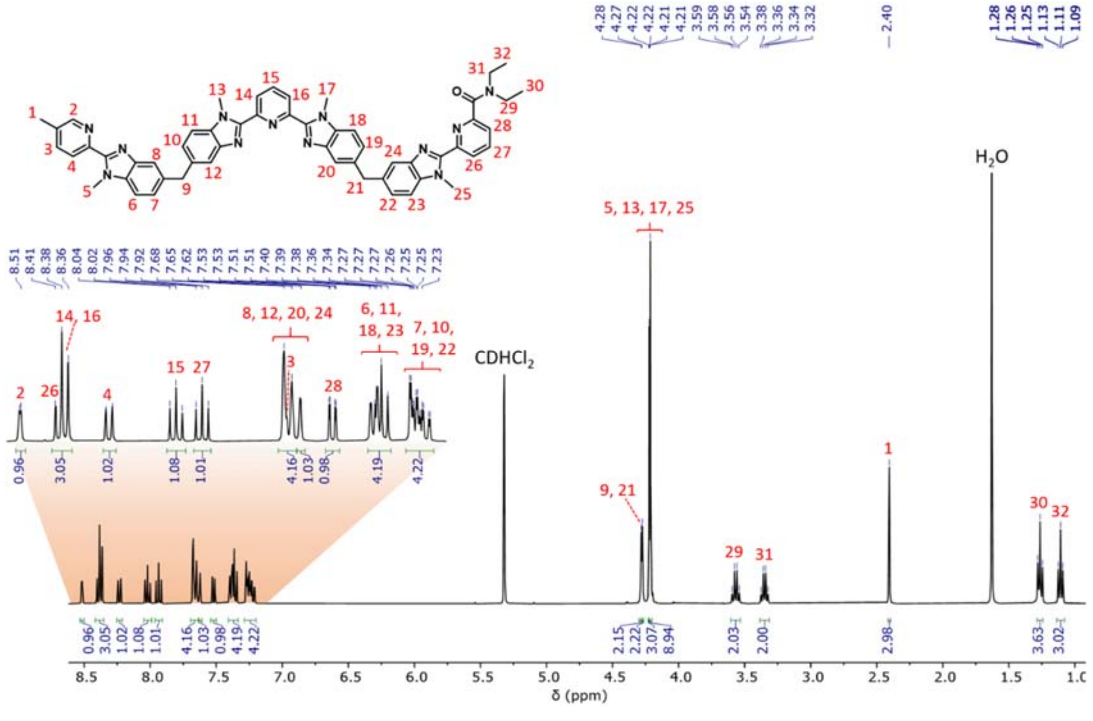

Fig. A2-10 ^{1}H-NMR spectrum of *N*,*N*-diethyl-6-(1-methyl-5-((1-methyl-2-(6-(1-methyl-5-((1-methyl-2-(5-methylpyridin-2-yl)-1*H*-benzo[d]imidazol-5-yl)methyl)-1*H*-benzo[*d*]imidazol-2-yl)pyridin-2-yl)-1*H*-benzo[*d*]imidazol-5-yl)methyl)-1*H*-benzo[*d*]imidazol-2-yl)picolinamide (**L4**). The aromatic region was expanded for clarity.

^{13}C-NMR (101 MHz, $CDCl_3$, 298 K) δ (ppm): 168.57, 154.94, 151.07, 150.81, 150.78, 150.27, 150.22, 150.19, 150.01, 149.32, 148.63, 143.43, 143.42, 143.38, 143.35, 138.24, 138.21, 137.67, 137.19, 137.07, 137.02, 136.70, 136.48, 136.42, 136.38, 136.36, 134.05, 125.35, 125.33, 125.21, 125.18, 125.16, 125.14, 124.67, 124.42, 122.80, 120.15, 120.14, 120.07, 119.87, 110.33, 110.29, 110.27, 110.16, 43.31, 42.57, 42.56, 40.05, 33.10, 33.04, 32.97, 32.96, 18.59, 14.54, 12.99.

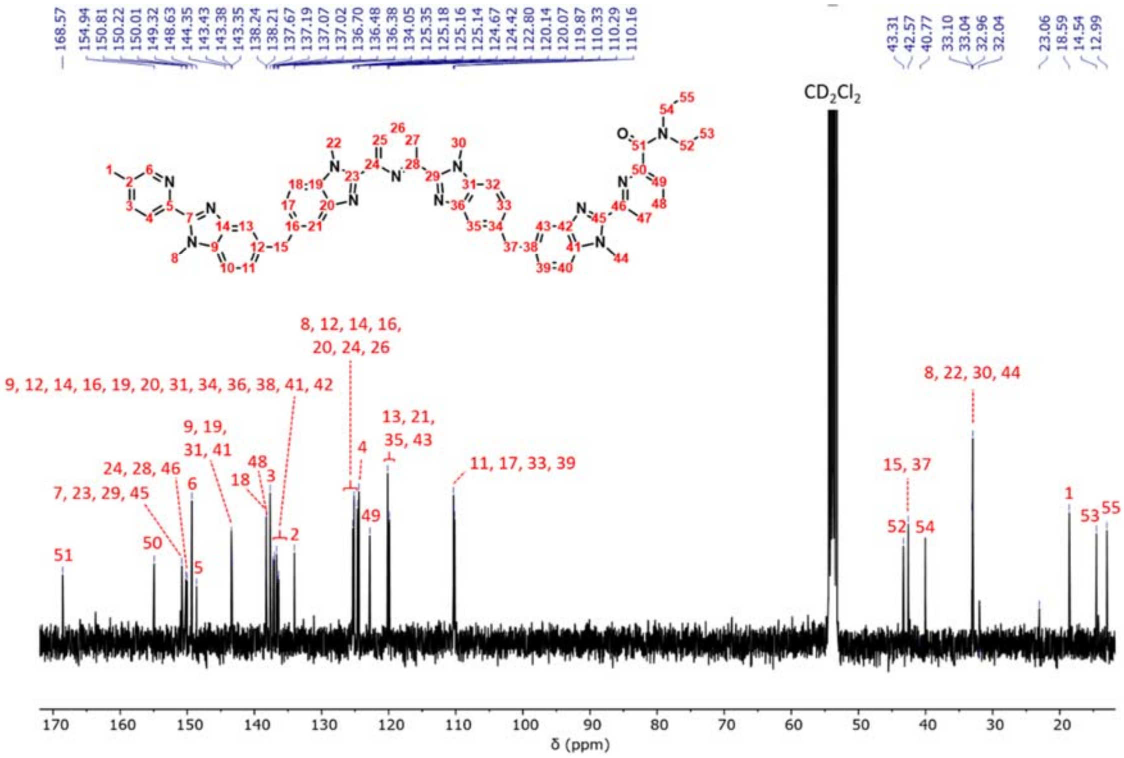

Fig. A2-11 ^{13}C-NMR spectrum of *N*,*N*-diethyl-6-(1-methyl-5-((1-methyl-2-(6-(1-methyl-5-((1-methyl-2-(5-methylpyridin-2-yl)-1*H*-benzo[d]imidazol-5-yl)methyl)-1*H*-benzo[*d*]imidazol-2-yl)pyridin-2-yl)-1*H*-benzo[*d*]imidazol-5-yl)methyl)-1*H*-benzo[*d*]imidazol-2-yl)picolinamide (**L4**).

Synthesis of heterodimetallic d-f-f complexes with L4.

These complexes were prepared *in situ* for ^{1}H-NMR studies. Stock solutions of $1.5\cdot10^{-2}$ M of $Zn(CF_3SO_3)_2$, and $3.0\cdot10^{-2}$ M of $La(CF_3SO_3)_3$, $Lu(CF_3SO_3)_3$ and $Eu(CF_3SO_3)_3$ were prepared in CD_3CN. 10 µL of D_2O were added to the solution of $La(CF_3SO_3)_3$ to facilitate the complete dissolution of the salt. 10.1 mg (11.2 µmol, 3 eq) of **L4** were solubilized in 250 µL of $CDCl_3$. 250 µL of the solution of $Zn(CF_3SO_3)_2$, and 250 µL of the solution of $Ln(CF_3SO_3)_3$ were successively added to give 750 µL of a 5.0 mM solution of $[(\mathbf{L4}_3Zn)Ln_2](CF_3SO_3)_8$.

Fragile pale yellow crystals of $[(\mathbf{L4}_3Zn)Eu_2](CF_3SO_3)_8\cdot12(C_3H_8O)$ suitable for X-ray diffraction studies were obtained by slow diffusion of isopropanol into a solution of the complex in acetonitrile at room temperature. Large cubic colorless crystals of $[(\mathbf{L4}_3Zn)La_2](CF_3SO_3)_8\cdot7.25(CH_3CN)$ suitable

for X-ray diffraction studies were obtained by slow diffusion of diethyl ether into a solution of the complex in acetonitrile at room temperature.

Synthesis of heterotrimetallic d-f-f ' complexes with L4.

These complexes were prepared *in situ* for ^{1}H-NMR studies. Stock solutions of $1.5\cdot10^{-2}$ M of $Zn(CF_3SO_3)_2$, and $3.0\cdot10^{-2}$ M of $La(CF_3SO_3)_3$, $Lu(CF_3SO_3)_3$ and $Eu(CF_3SO_3)_3$ were prepared in CD_3CN. 10 µL of D_2O were added to the solution of $La(CF_3SO_3)_3$ to facilitate the complete dissolution of the salt. 10.1 mg (11.2 µmol, 3 eq) of **L4** were solubilized in 250 µL of $CDCl_3$. 250 µL of the solution of $Zn(CF_3SO_3)_2$, 125 µL of the solution of $Ln^1(CF_3SO_3)_3$ and 125 µL of the solution of $Ln^2(CF_3SO_3)_3$ and were successively added to reach final concentrations of 15 mM of **L4**, and 5.0 mM of $Zn(CF_3SO_3)_2$, $Ln^A(CF_3SO_3)_3$ and $Ln^B(CF_3SO_3)_3$.

References

A2-1 G. M. Sheldrick, *Acta Cryst. C,* 2015, **71**, 3-8.

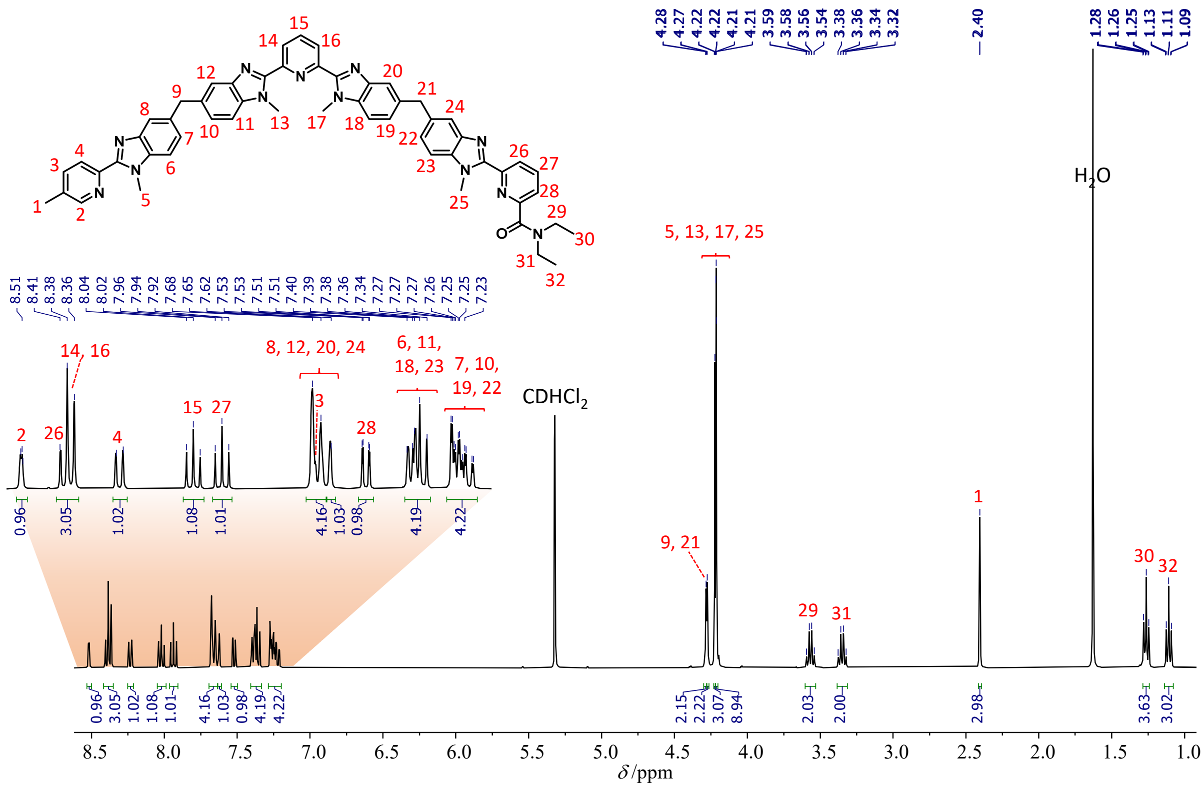

Fig. S1 ^{1}H-NMR spectrum of the ligand **L4**. The aromatic region was expanded for clarity.

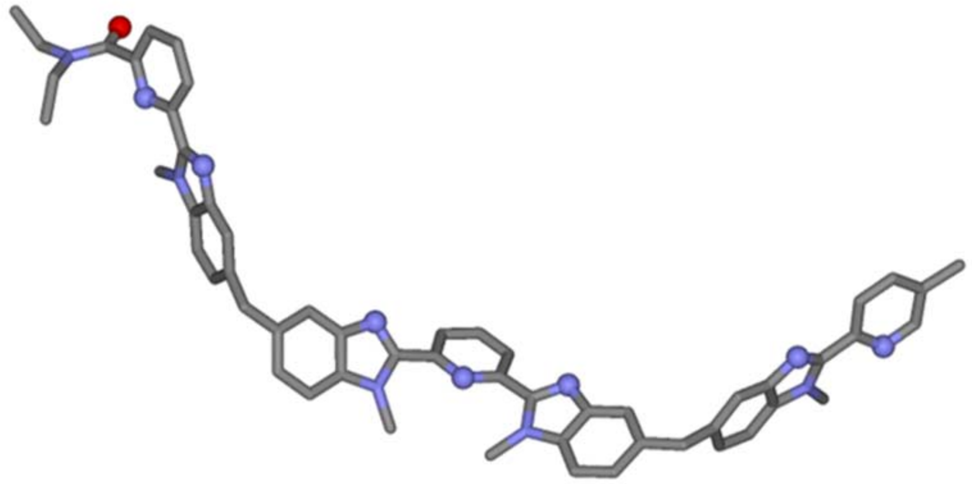

Fig. S2 Solid-state molecular structure of the ligand **L4**. The potential donor sites are highlighted with spheres. Hydrogen atoms and disordered solvent molecules are omitted for clarity. Color code: C = grey, N= blue, O = red.

Table S1. Crystal data and structure refinement for **L4**·C_3H_8O.

Compound	**L4**
Empirical formula	$C_{58}H_{58}N_{12}O_2$
Chemical formula	$(C_{55}H_{50}N_{12}O)(C_3H_8O)$
Formula weight	955.16
Temperature	120.00(10) K
Wavelength	1.54184 Å
Crystal system	Monoclinic
Space group	*C* 2/*c*
Unit cell dimensions	*a* = 37.7381(5) Å
	b = 6.47585(9) Å
	c = 42.6340(7) Å
	α = 90°
	β = 108.0349(17)°
	γ = 90°
Volume	9907.2(3) $Å^3$
Z	8
Density (calculated)	1.281 mg/m^3
Absorption coefficient	0.641 mm^{-1}
F(000)	4048
Crystal size	0.19 x 0.17 x 0.04 mm^3
Theta range for data collection	2.463 to 74.254°.
Index ranges	$-42 \leq h \leq 46$, $-7 \leq k \leq 7$, $-52 \leq l \leq 53$
Reflections collected	40064
Independent reflections	9853 [R(int) = 0.0379]
Completeness to theta = 67.684°	99.50%
Absorption correction	Analytical
Max. and min. transmission	0.977 and 0.900
Refinement method	Full-matrix least-squares on F^2
Data / restraints / parameters	9853 / 11 / 666
Goodness-of-fit on F^2	1.059
Final R indices [I>2sigma(I)]	R_1 = 0.0758, wR_2 = 0.2031
R indices (all data)	R_1 = 0.0935, wR_2 = 0.2156
Extinction coefficient	n/a
Largest diff. peak and hole	0.342 and -0.429 e.$Å^{-3}$

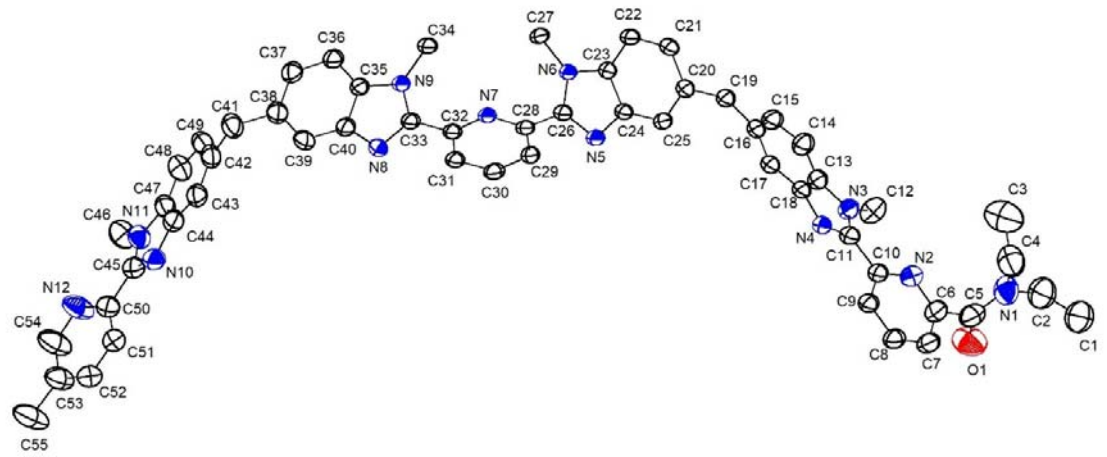

Fig. S3 ORTEP view of **L4** (thermal ellipsoids are drawn at 50% probability). Hydrogen atoms and the isopropanol solvent molecules (located around a 2-fold symmetry axis) are omitted for clarity reasons.

Table S2. Bond lengths (Å) and angles (°) for ligand **L4**·C_3H_8O

Bond lengths (Å)			
O(1)-C(5)	1.228(5)	C(29)-H(29)	0.95
N(1)-C(2)	1.469(5)	C(29)-C(30)	1.394(4)
N(1)-C(4)	1.459(5)	C(30)-H(30)	0.95
N(1)-C(5)	1.332(5)	C(30)-C(31)	1.375(4)
N(2)-C(6)	1.351(4)	C(31)-H(31)	0.95
N(2)-C(10)	1.346(4)	C(31)-C(32)	1.388(4)
N(3)-C(11)	1.380(4)	C(32)-C(33)	1.469(4)
N(3)-C(12)	1.458(4)	C(34)-H(34A)	0.98
N(3)-C(13)	1.381(4)	C(34)-H(34B)	0.98
N(4)-C(11)	1.319(4)	C(34)-H(34C)	0.98
N(4)-C(18)	1.383(4)	C(35)-C(36)	1.387(4)
N(5)-C(24)	1.392(3)	C(35)-C(40)	1.407(4)
N(5)-C(26)	1.326(3)	C(36)-H(36)	0.95
N(6)-C(23)	1.380(3)	C(36)-C(37)	1.384(4)
N(6)-C(26)	1.365(3)	C(37)-H(37)	0.95
N(6)-C(27)	1.457(3)	C(37)-C(38)	1.413(4)
N(7)-C(28)	1.342(3)	C(38)-C(39)	1.380(4)
N(7)-C(32)	1.352(3)	C(38)-C(41)	1.517(4)
N(8)-C(33)	1.322(3)	C(39)-H(39)	0.95

N(8)-C(40)	1.385(4)	C(39)-C(40)	1.397(4)
N(9)-C(33)	1.374(3)	C(41)-H(41A)	0.99
N(9)-C(34)	1.460(3)	C(41)-H(41B)	0.99
N(9)-C(35)	1.378(3)	C(41)-C(42)	1.515(5)
N(10)-C(44)	1.378(4)	C(42)-C(43)	1.380(5)
N(10)-C(45)	1.324(4)	C(42)-C(49)	1.411(5)
N(11)-C(45)	1.370(4)	C(43)-H(43)	0.95
N(11)-C(46)	1.456(4)	C(43)-C(44)	1.405(4)
N(11)-C(47)	1.391(4)	C(44)-C(47)	1.390(5)
N(12)-C(50)	1.339(5)	C(45)-C(50)	1.479(5)
N(12)-C(54)	1.341(5)	C(46)-H(46A)	0.98
C(1)-H(1A)	0.98	C(46)-H(46B)	0.98
C(1)-H(1B)	0.98	C(46)-H(46C)	0.98
C(1)-H(1C)	0.98	C(47)-C(48)	1.389(5)
C(1)-C(2)	1.488(7)	C(48)-H(48)	0.95
C(2)-H(2A)	0.99	C(48)-C(49)	1.381(5)
C(2)-H(2B)	0.99	C(49)-H(49)	0.95
C(3)-H(3A)	0.98	C(50)-C(51)	1.380(5)
C(3)-H(3B)	0.98	C(51)-H(51)	0.95
C(3)-H(3C)	0.98	C(51)-C(52)	1.376(5)
C(3)-C(4)	1.478(8)	C(52)-H(52)	0.95
C(4)-H(4A)	0.99	C(52)-C(53)	1.370(5)
C(4)-H(4B)	0.99	C(53)-C(54)	1.382(6)
C(5)-C(6)	1.507(5)	C(53)-C(55)	1.523(6)
C(6)-C(7)	1.366(5)	C(54)-H(54)	0.95
C(7)-H(7)	0.95	C(55)-H(55A)	0.98
C(7)-C(8)	1.375(5)	C(55)-H(55B)	0.98
C(8)-H(8)	0.95	C(55)-H(55C)	0.98
C(8)-C(9)	1.390(4)	C(58)-H(58A)	1
C(9)-H(9)	0.95	C(58)-H(58)	1
C(9)-C(10)	1.378(4)	C(58)-C(56A)	1.552(17)
C(10)-C(11)	1.476(4)	C(58)-O(3A)	1.303(14)
C(12)-H(12A)	0.98	C(58)-C(57A)	1.576(17)
C(12)-H(12B)	0.98	C(58)-C(56B)	1.577(18)

C(12)-H(12C)	0.98	C(58)-O(3B)	1.402(15)
C(13)-C(14)	1.406(4)	C(58)-C(57B)	1.561(18)
C(13)-C(18)	1.391(4)	C(56A)-H(56A)	0.9798
C(14)-H(14)	0.95	C(56A)-H(56B)	0.9805
C(14)-C(15)	1.379(4)	C(56A)-H(56C)	0.9793
C(15)-H(15)	0.95	O(3A)-H(3AA)	0.7788
C(15)-C(16)	1.401(4)	C(57A)-H(57A)	0.9807
C(16)-C(17)	1.385(4)	C(57A)-H(57B)	0.9798
C(16)-C(19)	1.514(4)	C(57A)-H(57C)	0.9795
C(17)-H(17)	0.95	C(56B)-H(56D)	0.9802
C(17)-C(18)	1.404(4)	C(56B)-H(56E)	0.9805
C(19)-H(19A)	0.99	C(56B)-H(56F)	0.9807
C(19)-H(19B)	0.99	O(3B)-H(3BA)	0.8688
C(19)-C(20)	1.520(4)	C(57B)-H(57D)	0.9798
C(20)-C(21)	1.402(4)	C(57B)-H(57E)	0.9802
C(20)-C(25)	1.386(4)	C(57B)-H(57F)	0.98
C(21)-H(21)	0.95	O(1S)-H(1S)	0.8351
C(21)-C(22)	1.385(4)	O(1S)-C(2S)	1.296(16)
C(22)-H(22)	0.95	C(2S)-H(2S)	1
C(22)-C(23)	1.389(4)	C(2S)-C(4S)	1.443(17)
C(23)-C(24)	1.403(4)	C(2S)-C(3S)	1.439(17)
C(24)-C(25)	1.396(4)	C(4S)-H(4SA)	0.9807
C(25)-H(25)	0.95	C(4S)-H(4SB)	0.979
C(26)-C(28)	1.477(3)	C(4S)-H(4SC)	0.9811
C(27)-H(27A)	0.98	C(3S)-H(3SA)	0.9802
C(27)-H(27B)	0.98	C(3S)-H(3SB)	0.9797
C(27)-H(27C)	0.98	C(3S)-H(3SC)	0.9798
C(28)-C(29)	1.388(4)		

Bond angles (°)			
C(4)-N(1)-C(2)	116.5(4)	N(8)-C(33)-N(9)	112.8(2)
C(5)-N(1)-C(2)	120.4(4)	N(8)-C(33)-C(32)	122.5(2)
C(5)-N(1)-C(4)	123.0(3)	N(9)-C(33)-C(32)	124.7(2)
C(10)-N(2)-C(6)	117.1(3)	N(9)-C(34)-H(34A)	109.5

C(11)-N(3)-C(12)	129.9(3)	N(9)-C(34)-H(34B)	109.5
C(11)-N(3)-C(13)	105.6(3)	N(9)-C(34)-H(34C)	109.5
C(13)-N(3)-C(12)	124.5(3)	H(34A)-C(34)-H(34B)	109.5
C(11)-N(4)-C(18)	105.0(3)	H(34A)-C(34)-H(34C)	109.5
C(26)-N(5)-C(24)	104.6(2)	H(34B)-C(34)-H(34C)	109.5
C(23)-N(6)-C(27)	124.2(2)	N(9)-C(35)-C(36)	132.1(3)
C(26)-N(6)-C(23)	106.6(2)	N(9)-C(35)-C(40)	105.4(2)
C(26)-N(6)-C(27)	129.2(2)	C(36)-C(35)-C(40)	122.4(3)
C(28)-N(7)-C(32)	116.9(2)	C(35)-C(36)-H(36)	121.8
C(33)-N(8)-C(40)	105.0(2)	C(37)-C(36)-C(35)	116.5(3)
C(33)-N(9)-C(34)	128.9(2)	C(37)-C(36)-H(36)	121.8
C(33)-N(9)-C(35)	106.7(2)	C(36)-C(37)-H(37)	118.7
C(35)-N(9)-C(34)	124.3(2)	C(36)-C(37)-C(38)	122.5(3)
C(45)-N(10)-C(44)	104.3(3)	C(38)-C(37)-H(37)	118.7
C(45)-N(11)-C(46)	130.7(3)	C(37)-C(38)-C(41)	118.9(3)
C(45)-N(11)-C(47)	106.0(3)	C(39)-C(38)-C(37)	119.8(3)
C(47)-N(11)-C(46)	123.3(3)	C(39)-C(38)-C(41)	121.3(3)
C(50)-N(12)-C(54)	117.2(3)	C(38)-C(39)-H(39)	120.5
H(1A)-C(1)-H(1B)	109.5	C(38)-C(39)-C(40)	119.0(3)
H(1A)-C(1)-H(1C)	109.5	C(40)-C(39)-H(39)	120.5
H(1B)-C(1)-H(1C)	109.5	N(8)-C(40)-C(35)	110.0(2)
C(2)-C(1)-H(1A)	109.5	N(8)-C(40)-C(39)	130.3(3)
C(2)-C(1)-H(1B)	109.5	C(39)-C(40)-C(35)	119.7(3)
C(2)-C(1)-H(1C)	109.5	C(38)-C(41)-H(41A)	108.4
N(1)-C(2)-C(1)	113.8(4)	C(38)-C(41)-H(41B)	108.4
N(1)-C(2)-H(2A)	108.8	H(41A)-C(41)-H(41B)	107.4
N(1)-C(2)-H(2B)	108.8	C(42)-C(41)-C(38)	115.6(3)
C(1)-C(2)-H(2A)	108.8	C(42)-C(41)-H(41A)	108.4
C(1)-C(2)-H(2B)	108.8	C(42)-C(41)-H(41B)	108.4
H(2A)-C(2)-H(2B)	107.7	C(43)-C(42)-C(41)	120.7(3)
H(3A)-C(3)-H(3B)	109.5	C(43)-C(42)-C(49)	120.1(3)
H(3A)-C(3)-H(3C)	109.5	C(49)-C(42)-C(41)	119.1(3)
H(3B)-C(3)-H(3C)	109.5	C(42)-C(43)-H(43)	120.5
C(4)-C(3)-H(3A)	109.5	C(42)-C(43)-C(44)	118.9(3)

C(4)-C(3)-H(3B)	109.5	C(44)-C(43)-H(43)	120.5
C(4)-C(3)-H(3C)	109.5	N(10)-C(44)-C(43)	129.5(3)
N(1)-C(4)-C(3)	111.3(5)	N(10)-C(44)-C(47)	111.0(3)
N(1)-C(4)-H(4A)	109.4	C(47)-C(44)-C(43)	119.4(3)
N(1)-C(4)-H(4B)	109.4	N(10)-C(45)-N(11)	113.4(3)
C(3)-C(4)-H(4A)	109.4	N(10)-C(45)-C(50)	121.5(3)
C(3)-C(4)-H(4B)	109.4	N(11)-C(45)-C(50)	125.1(3)
H(4A)-C(4)-H(4B)	108	N(11)-C(46)-H(46A)	109.5
O(1)-C(5)-N(1)	122.2(4)	N(11)-C(46)-H(46B)	109.5
O(1)-C(5)-C(6)	119.0(4)	N(11)-C(46)-H(46C)	109.5
N(1)-C(5)-C(6)	118.7(3)	H(46A)-C(46)-H(46B)	109.5
N(2)-C(6)-C(5)	115.9(3)	H(46A)-C(46)-H(46C)	109.5
N(2)-C(6)-C(7)	123.9(3)	H(46B)-C(46)-H(46C)	109.5
C(7)-C(6)-C(5)	120.2(3)	C(44)-C(47)-N(11)	105.3(3)
C(6)-C(7)-H(7)	120.9	C(48)-C(47)-N(11)	131.9(3)
C(6)-C(7)-C(8)	118.3(3)	C(48)-C(47)-C(44)	122.8(3)
C(8)-C(7)-H(7)	120.9	C(47)-C(48)-H(48)	121.6
C(7)-C(8)-H(8)	120.3	C(49)-C(48)-C(47)	116.8(3)
C(7)-C(8)-C(9)	119.3(3)	C(49)-C(48)-H(48)	121.6
C(9)-C(8)-H(8)	120.3	C(42)-C(49)-H(49)	119
C(8)-C(9)-H(9)	120.6	C(48)-C(49)-C(42)	121.9(3)
C(10)-C(9)-C(8)	118.8(3)	C(48)-C(49)-H(49)	119
C(10)-C(9)-H(9)	120.6	N(12)-C(50)-C(45)	118.3(3)
N(2)-C(10)-C(9)	122.5(3)	N(12)-C(50)-C(51)	121.9(3)
N(2)-C(10)-C(11)	118.5(3)	C(51)-C(50)-C(45)	119.8(3)
C(9)-C(10)-C(11)	119.0(3)	C(50)-C(51)-H(51)	120.3
N(3)-C(11)-C(10)	124.9(3)	C(52)-C(51)-C(50)	119.4(3)
N(4)-C(11)-N(3)	113.1(3)	C(52)-C(51)-H(51)	120.3
N(4)-C(11)-C(10)	122.0(3)	C(51)-C(52)-H(52)	119.9
N(3)-C(12)-H(12A)	109.5	C(53)-C(52)-C(51)	120.1(4)
N(3)-C(12)-H(12B)	109.5	C(53)-C(52)-H(52)	119.9
N(3)-C(12)-H(12C)	109.5	C(52)-C(53)-C(54)	116.6(4)
H(12A)-C(12)-H(12B)	109.5	C(52)-C(53)-C(55)	122.4(4)
H(12A)-C(12)-H(12C)	109.5	C(54)-C(53)-C(55)	120.9(4)

H(12B)-C(12)-H(12C)	109.5	N(12)-C(54)-C(53)	124.7(4)
N(3)-C(13)-C(14)	131.9(3)	N(12)-C(54)-H(54)	117.7
N(3)-C(13)-C(18)	106.4(3)	C(53)-C(54)-H(54)	117.7
C(18)-C(13)-C(14)	121.8(3)	C(53)-C(55)-H(55A)	109.5
C(13)-C(14)-H(14)	121.7	C(53)-C(55)-H(55B)	109.5
C(15)-C(14)-C(13)	116.6(3)	C(53)-C(55)-H(55C)	109.5
C(15)-C(14)-H(14)	121.7	H(55A)-C(55)-H(55B)	109.5
C(14)-C(15)-H(15)	118.6	H(55A)-C(55)-H(55C)	109.5
C(14)-C(15)-C(16)	122.8(3)	H(55B)-C(55)-H(55C)	109.5
C(16)-C(15)-H(15)	118.6	C(56A)-C(58)-H(58A)	111.7
C(15)-C(16)-C(19)	119.9(3)	C(56A)-C(58)-C(57A)	105(2)
C(17)-C(16)-C(15)	119.8(3)	O(3A)-C(58)-H(58A)	111.7
C(17)-C(16)-C(19)	120.1(3)	O(3A)-C(58)-C(56A)	122.3(17)
C(16)-C(17)-H(17)	120.7	O(3A)-C(58)-C(57A)	91.9(13)
C(16)-C(17)-C(18)	118.7(3)	C(57A)-C(58)-H(58A)	111.7
C(18)-C(17)-H(17)	120.7	C(56B)-C(58)-H(58)	108
N(4)-C(18)-C(13)	110.0(3)	O(3B)-C(58)-H(58)	108
N(4)-C(18)-C(17)	129.7(3)	O(3B)-C(58)-C(56B)	104(2)
C(13)-C(18)-C(17)	120.3(3)	O(3B)-C(58)-C(57B)	105.1(13)
C(16)-C(19)-H(19A)	108.4	C(57B)-C(58)-H(58)	108
C(16)-C(19)-H(19B)	108.4	C(57B)-C(58)-C(56B)	123(2)
C(16)-C(19)-C(20)	115.6(2)	C(58)-C(56A)-H(56A)	111.9
H(19A)-C(19)-H(19B)	107.4	C(58)-C(56A)-H(56B)	107.8
C(20)-C(19)-H(19A)	108.4	C(58)-C(56A)-H(56C)	108.7
C(20)-C(19)-H(19B)	108.4	H(56A)-C(56A)-H(56B)	109.5
C(21)-C(20)-C(19)	119.6(2)	H(56A)-C(56A)-H(56C)	109.6
C(25)-C(20)-C(19)	120.2(2)	H(56B)-C(56A)-H(56C)	109.4
C(25)-C(20)-C(21)	120.1(2)	C(58)-O(3A)-H(3AA)	110.6
C(20)-C(21)-H(21)	118.7	C(58)-C(57A)-H(57A)	108.8
C(22)-C(21)-C(20)	122.6(2)	C(58)-C(57A)-H(57B)	109.3
C(22)-C(21)-H(21)	118.7	C(58)-C(57A)-H(57C)	110.3
C(21)-C(22)-H(22)	121.9	H(57A)-C(57A)-H(57B)	109.5
C(21)-C(22)-C(23)	116.3(2)	H(57A)-C(57A)-H(57C)	109.4
C(23)-C(22)-H(22)	121.9	H(57B)-C(57A)-H(57C)	109.5

N(6)-C(23)-C(22)	131.8(2)	C(58)-C(56B)-H(56D)	110.9
N(6)-C(23)-C(24)	105.7(2)	C(58)-C(56B)-H(56E)	109.7
C(22)-C(23)-C(24)	122.5(2)	C(58)-C(56B)-H(56F)	107.9
N(5)-C(24)-C(23)	109.8(2)	H(56D)-C(56B)-H(56E)	109.5
N(5)-C(24)-C(25)	130.4(2)	H(56D)-C(56B)-H(56F)	109.4
C(25)-C(24)-C(23)	119.8(2)	H(56E)-C(56B)-H(56F)	109.5
C(20)-C(25)-C(24)	118.5(2)	C(58)-O(3B)-H(3BA)	103.3
C(20)-C(25)-H(25)	120.7	C(58)-C(57B)-H(57D)	109.4
C(24)-C(25)-H(25)	120.7	C(58)-C(57B)-H(57E)	110.4
N(5)-C(26)-N(6)	113.3(2)	C(58)-C(57B)-H(57F)	108.6
N(5)-C(26)-C(28)	122.9(2)	H(57D)-C(57B)-H(57E)	109.5
N(6)-C(26)-C(28)	123.8(2)	H(57D)-C(57B)-H(57F)	109.5
N(6)-C(27)-H(27A)	109.5	H(57E)-C(57B)-H(57F)	109.5
N(6)-C(27)-H(27B)	109.5	C(2S)-O(1S)-H(1S)	107.1
N(6)-C(27)-H(27C)	109.5	O(1S)-C(2S)-H(2S)	94.3
H(27A)-C(27)-H(27B)	109.5	O(1S)-C(2S)-C(4S)	97.0(15)
H(27A)-C(27)-H(27C)	109.5	O(1S)-C(2S)-C(3S)	118(2)
H(27B)-C(27)-H(27C)	109.5	C(4S)-C(2S)-H(2S)	94.3
N(7)-C(28)-C(26)	117.2(2)	C(3S)-C(2S)-H(2S)	94.3
N(7)-C(28)-C(29)	123.9(2)	C(3S)-C(2S)-C(4S)	143(3)
C(29)-C(28)-C(26)	118.8(2)	C(2S)-C(4S)-H(4SA)	111.5
C(28)-C(29)-H(29)	120.9	C(2S)-C(4S)-H(4SB)	108.2
C(28)-C(29)-C(30)	118.3(3)	C(2S)-C(4S)-H(4SC)	108.6
C(30)-C(29)-H(29)	120.9	H(4SA)-C(4S)-H(4SB)	109.6
C(29)-C(30)-H(30)	120.7	H(4SA)-C(4S)-H(4SC)	109.4
C(31)-C(30)-C(29)	118.5(3)	H(4SB)-C(4S)-H(4SC)	109.5
C(31)-C(30)-H(30)	120.7	C(2S)-C(3S)-H(3SA)	107.3
C(30)-C(31)-H(31)	120.1	C(2S)-C(3S)-H(3SB)	111
C(30)-C(31)-C(32)	119.7(2)	C(2S)-C(3S)-H(3SC)	110.1
C(32)-C(31)-H(31)	120.1	H(3SA)-C(3S)-H(3SB)	109.4
N(7)-C(32)-C(31)	122.6(2)	H(3SA)-C(3S)-H(3SC)	109.5
N(7)-C(32)-C(33)	117.8(2)	H(3SB)-C(3S)-H(3SC)	109.5
C(31)-C(32)-C(33)	119.5(2)		

Table S3. Planes data for ligand **L4**·C_3H_8O.

Plane description	Interplanar angle (°)
Pyridine 1<br>N(12) C(50) C(51) C(52) C(53) C(54)<br>Benzimidazole 1<br>C(45) N(10) C(44) C(43) C(42) C(49) C(48) C(47) N(11)	20.83
Pyridine 2<br>N(7) C(28) C(29) C(30) C(31) C(32)<br>Benzimidazole 2<br>C(33) N(9) C(35) C(36) C(37) C(38) C(39) C(40) N(8)	31.19
Pyridine 2<br>N(7) C(28) C(29) C(30) C(31) C(32)<br>Imidazole 3<br>N(5) C(26) N(6) C(23) C(24)	45.07
Pyridine 3<br>N(12) C(50) C(51) C(52) C(53) C(54)<br>Benzimidazole 4<br>N(11) C(45) N(10) C(44) C(47)	16.45
Pyridine 3<br>N(12) C(50) C(51) C(52) C(53) C(54)<br>Carbonyl group<br>O(1) C(5) N(1) C(6)	72.31

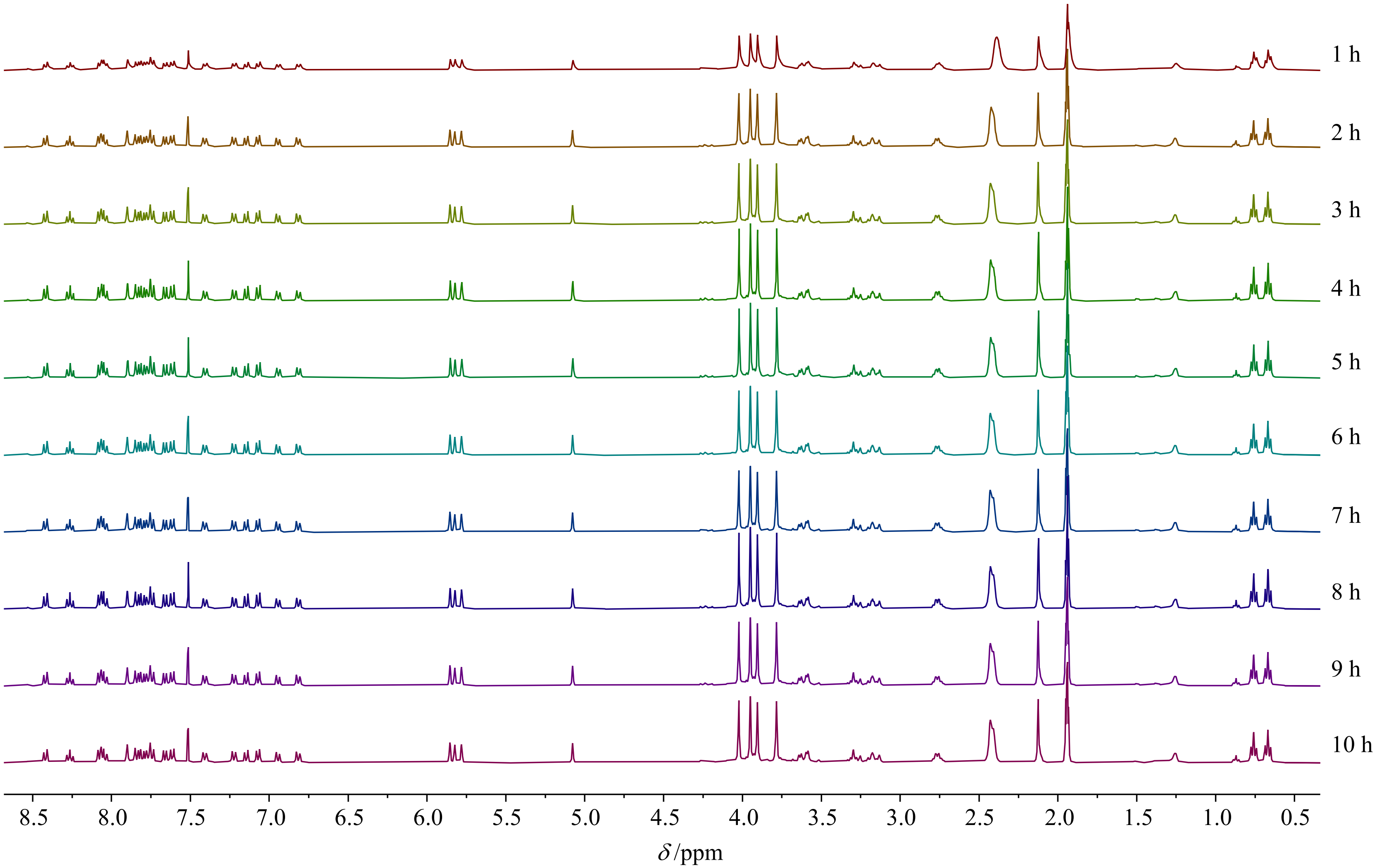

Fig. S4 Kinetics of formation of the *HHH*-[(**L4**$_3$Zn)La$_2$]$^{8+}$ complex over 10 hours after the concomitant mixing of a 1:2:3 mixture of Zn(CF$_3$SO$_3$)$_2$, La(CF$_3$SO$_3$)$_3$ and **L4** (^{1}H-NMR, 400 MHz, 2:1 CD$_3$CN/CDCl$_3$, 298 K).

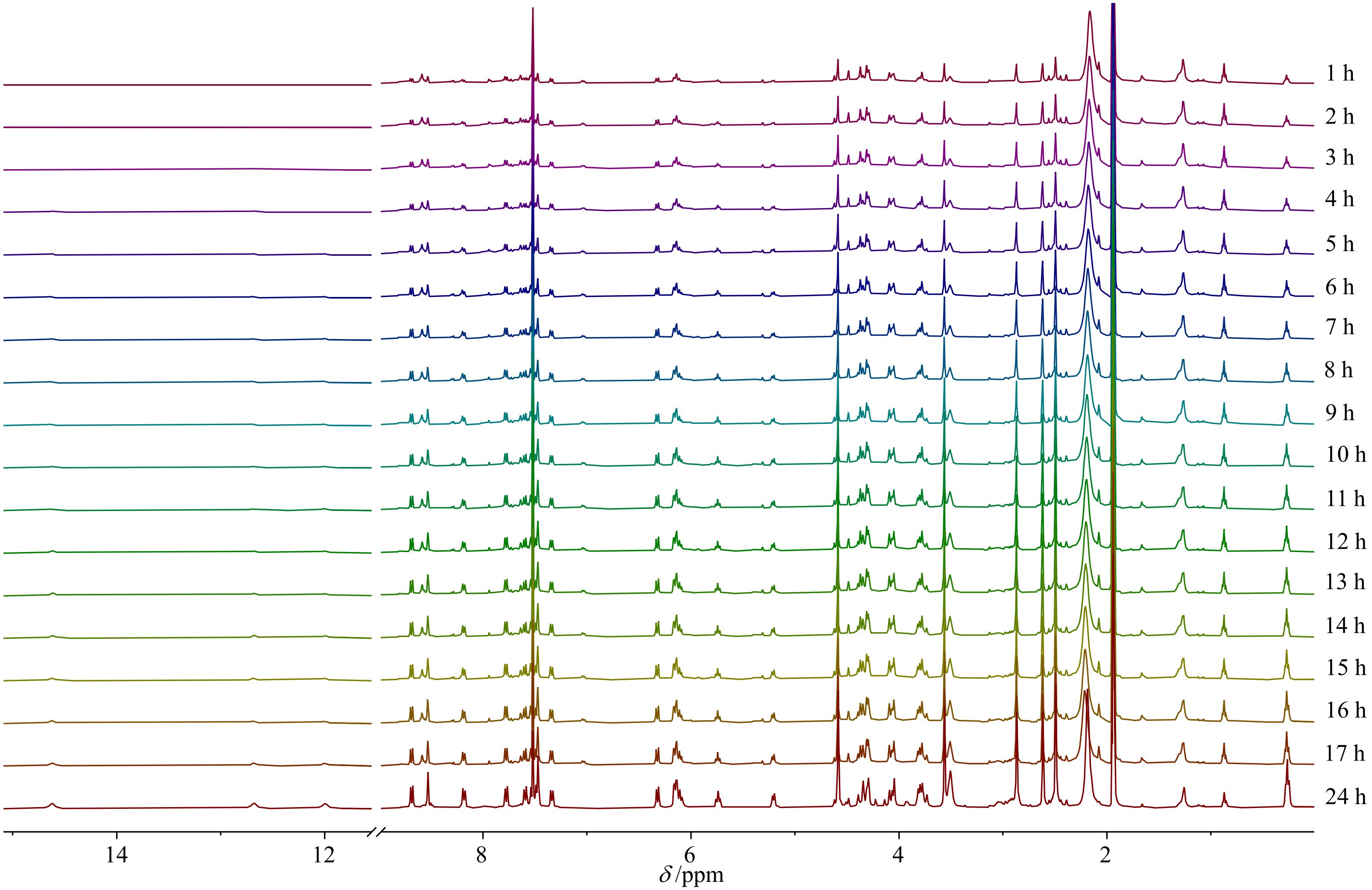

Fig. S5 Kinetics of formation of the *HHH*-[(**L4**$_3$Zn)Eu$_2$]$^{8+}$ complex over 24 hours after the concomitant mixing of a 1:2:3 mixture of Zn(CF_3SO_3)$_2$, Eu(CF_3SO_3)$_3$ and **L4** (^{1}H-NMR, 400 MHz, 2:1 CD_3CN/$CDCl_3$, 298 K).

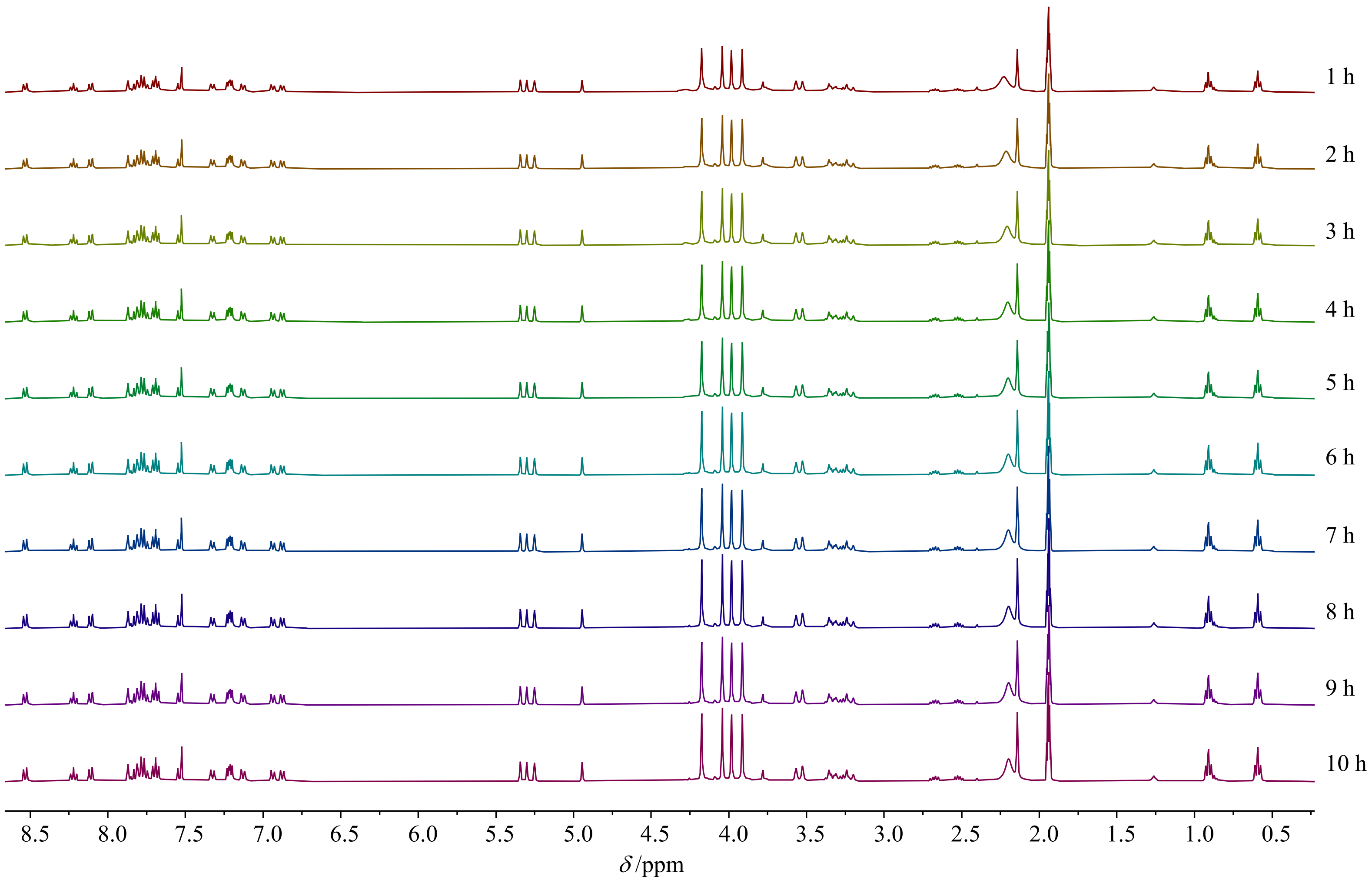

Fig. S6 Kinetics of formation of *HHH*-[(**L4**$_3$Zn)Lu$_2$]$^{8+}$ over 10 hours after the concomitant mixing of a 1:2:3 mixture of Zn(CF_3SO_3)$_2$, Lu(CF_3SO_3)$_2$ and **L4** (^{1}H-NMR, 400 MHz, 2:1 CD_3CN/$CDCl_3$, 298 K).

The 31 signals arising from the three wrapped ligand strands could be fully assigned with the help of correlation (Figs S9-S12) and Nuclear Overhauser Enhancement (NOE) spectroscopies (Figs S13-S15). The loss of planar symmetry produced by the helical wrapping of the ligand strands in the final helicates is ascertained by the methylene protons 9, 21, 29 and 31, which become diastereotopic. The usual *transoid*-to-*cisoid* conformation changes of the benzimidazole-pyridine units required for coordinating the metal ions are confirmed by the presence of NOE correlations between the protons 4, 14, 16 and 26 and the methyl groups 5, 13, 17 and 25, respectively (Figs S13-S15).

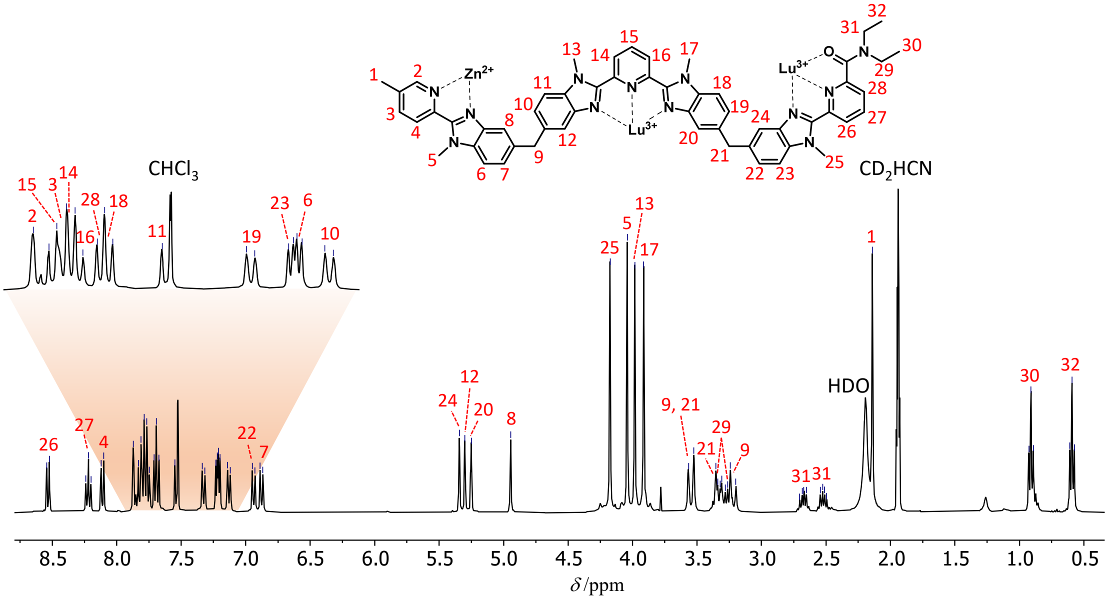

Fig. S7 ^{1}H-NMR spectrum of *HHH*-[(**L4**$_3$Zn)Lu$_2$]$^{8+}$ complex (2:1 $CD_3CN/CDCl_3$, 400 MHz, 298 K). The aromatic region is expanded for clarity.

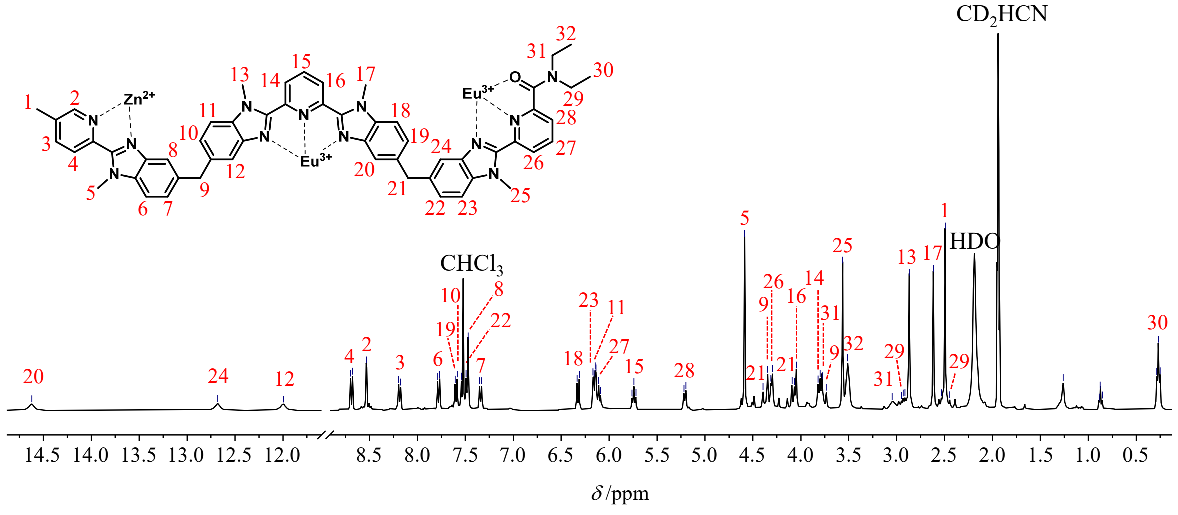

Fig. S8 ^{1}H-NMR spectrum of *HHH*-[(**L4**$_3$Zn)Eu$_2$]$^{8+}$ complex (2:1 $CD_3CN/CDCl_3$, 400 MHz, 298 K).

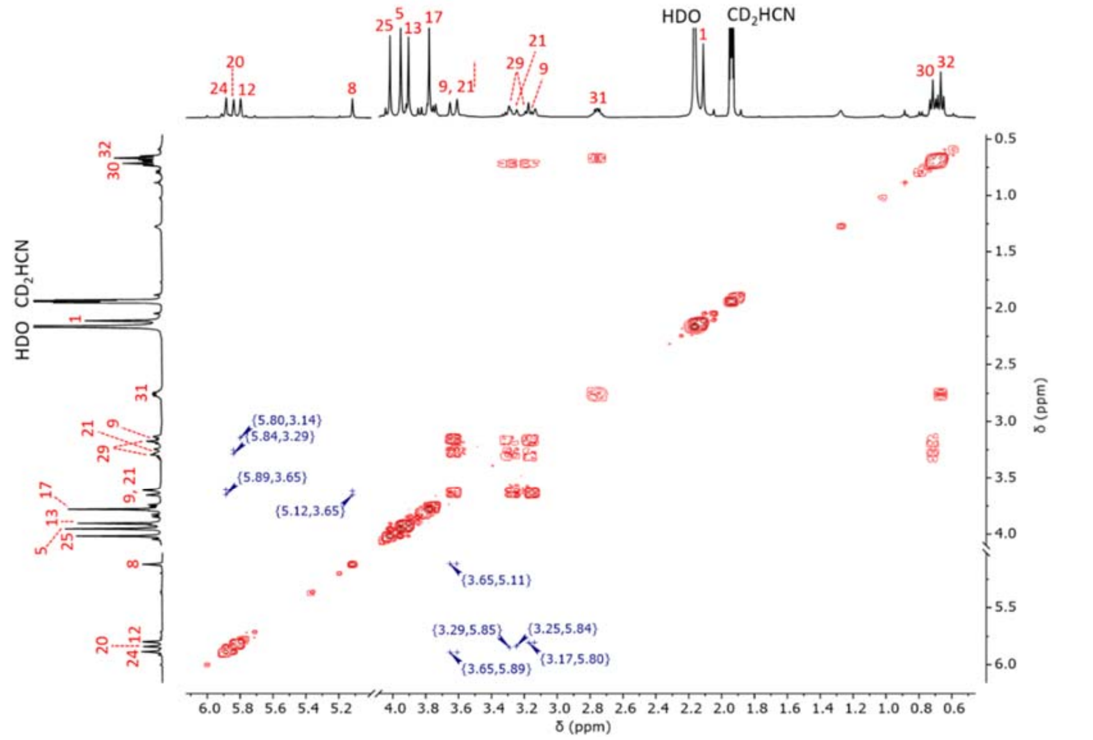

Fig. S9 COSY (CD_3CN, 400 MHz, 298 K) of the *HHH*-[($\mathbf{L4}_3$Zn)La_2]$^{8+}$ complex. Relevant ^4J correlations between the methylene protons 9 and 21 and the benzimidazole protons 8, 12, 20 and 24 are highlighted.

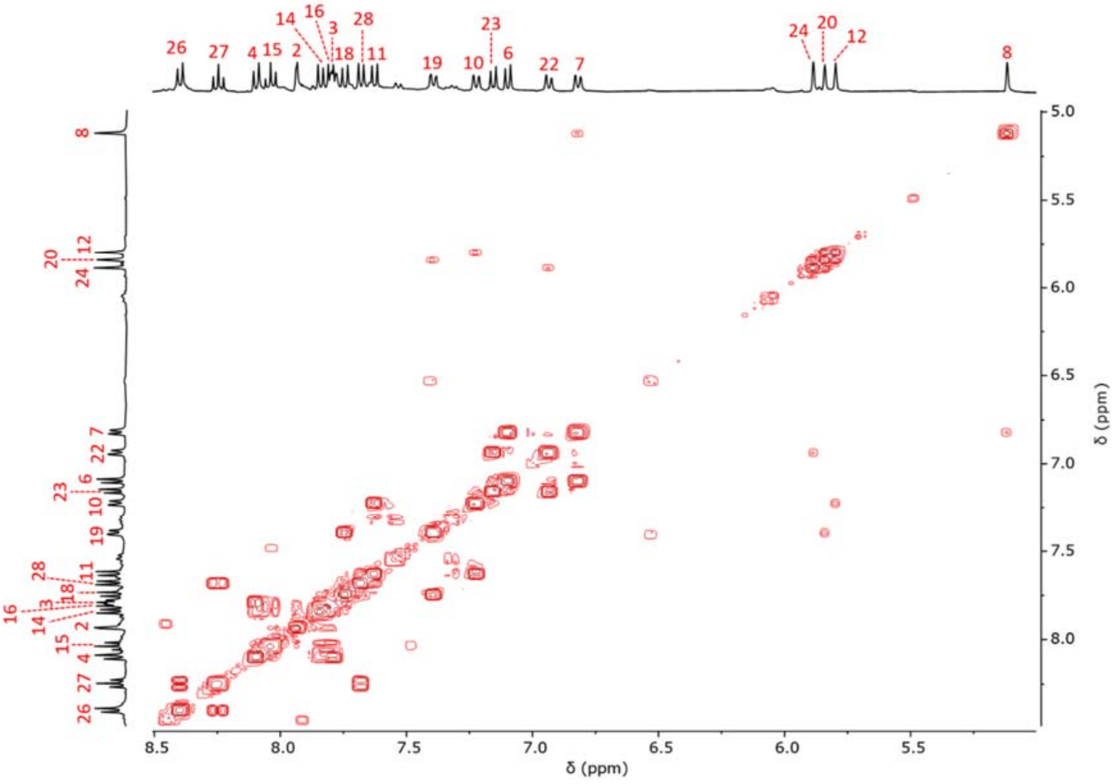

Fig. S10 COSY (CD_3CN, 400 MHz, 298 K) of the *HHH*-[($\mathbf{L4}_3$Zn)La_2]$^{8+}$ complex.

a)

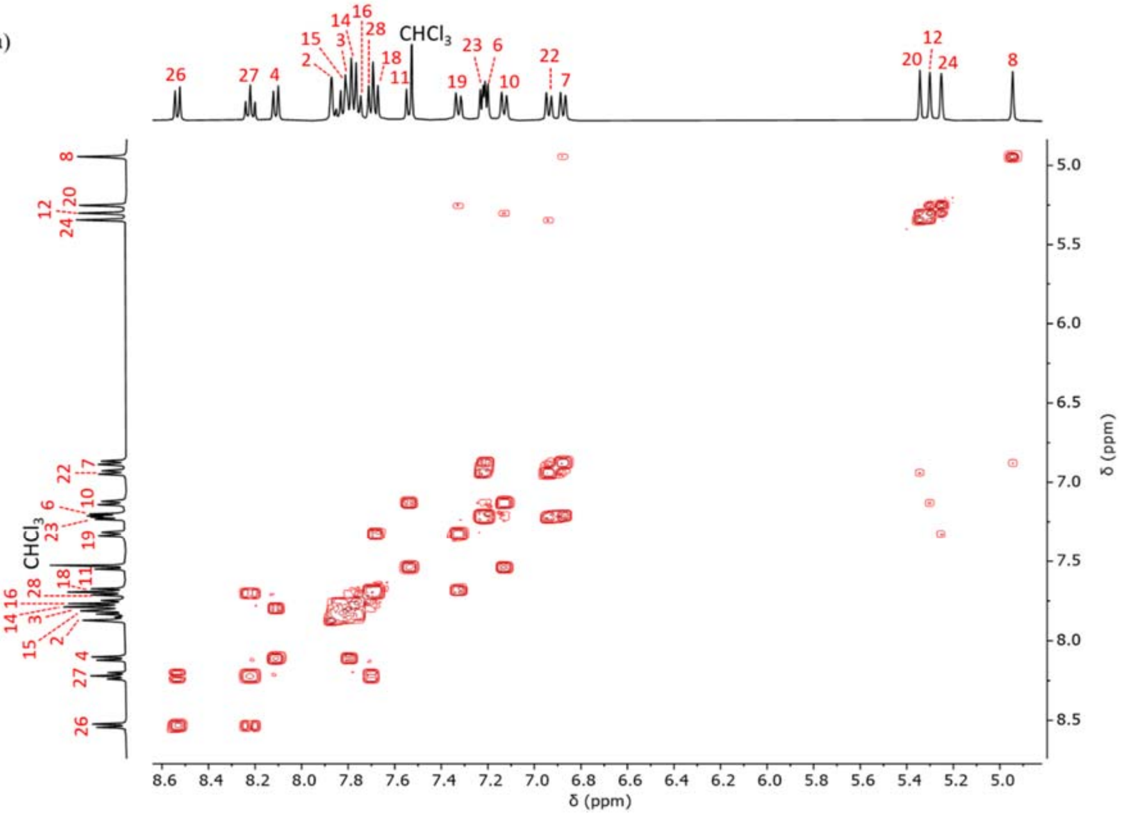

b)

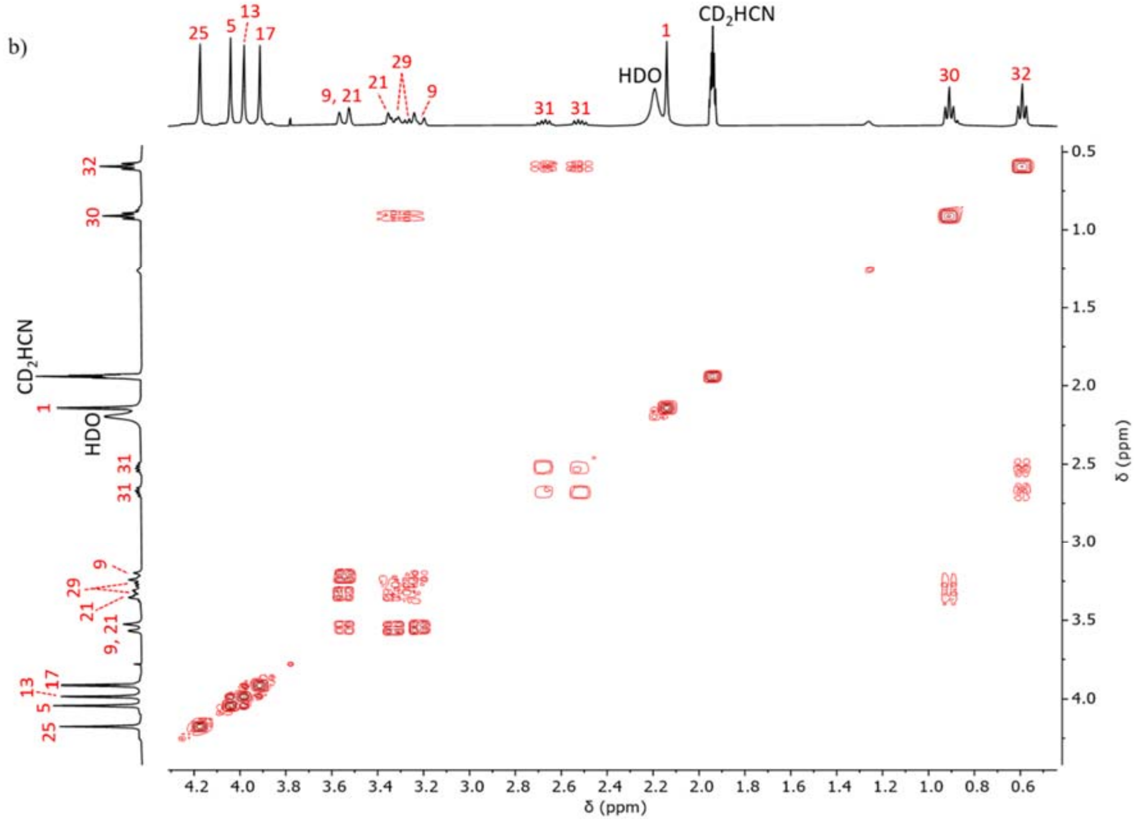

Fig. S11 COSY (2:1 $CD_3CN/CDCl_3$, 400 MHz, 298 K) of the *HHH*-[(**L4**$_3$Zn)Lu$_2$]$^{8+}$ complex.

a)

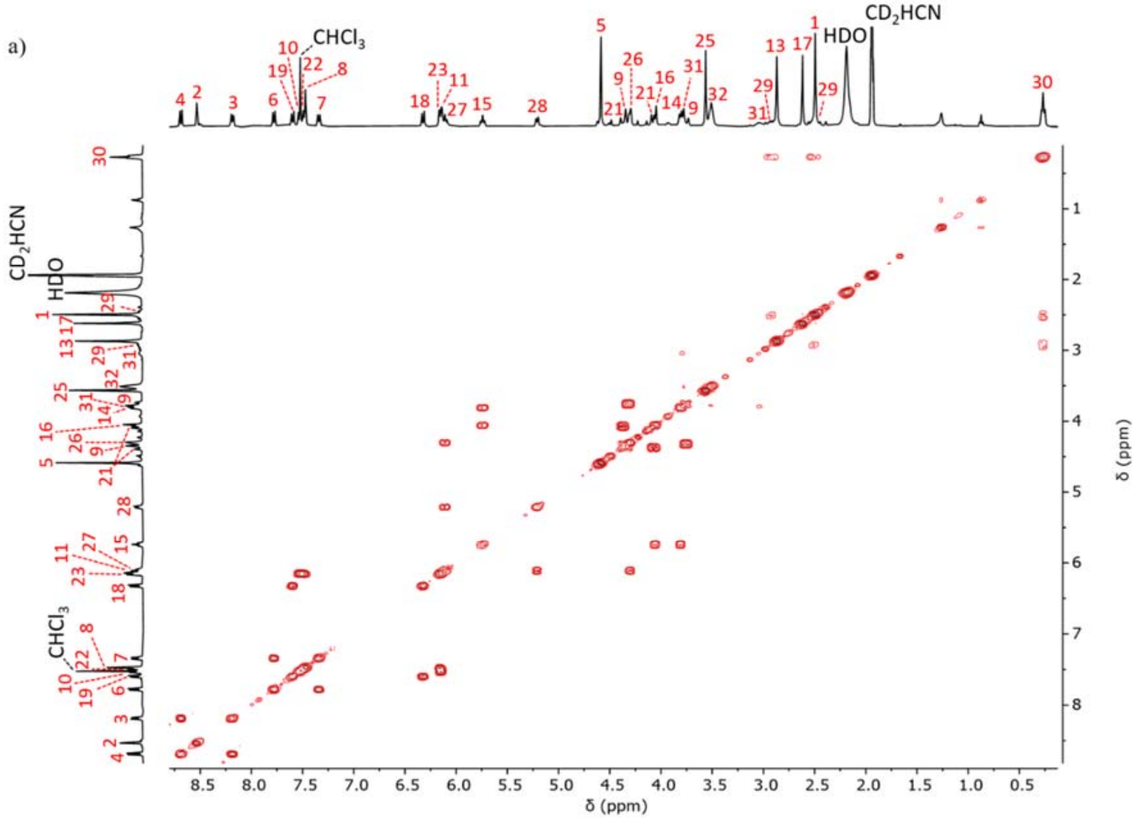

b)

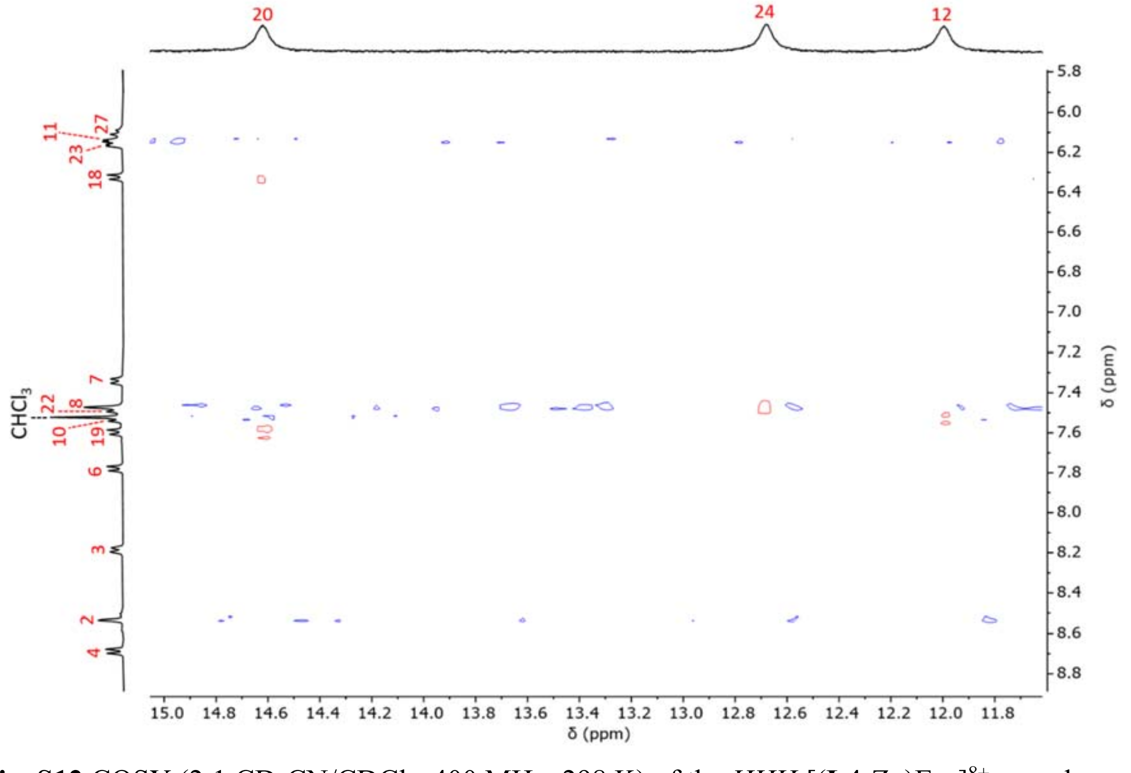

Fig. S12 COSY (2:1 $CD_3CN/CDCl_3$, 400 MHz, 298 K) of the *HHH*-[(**L4**$_3$Zn)Eu_2]$^{8+}$ complex.

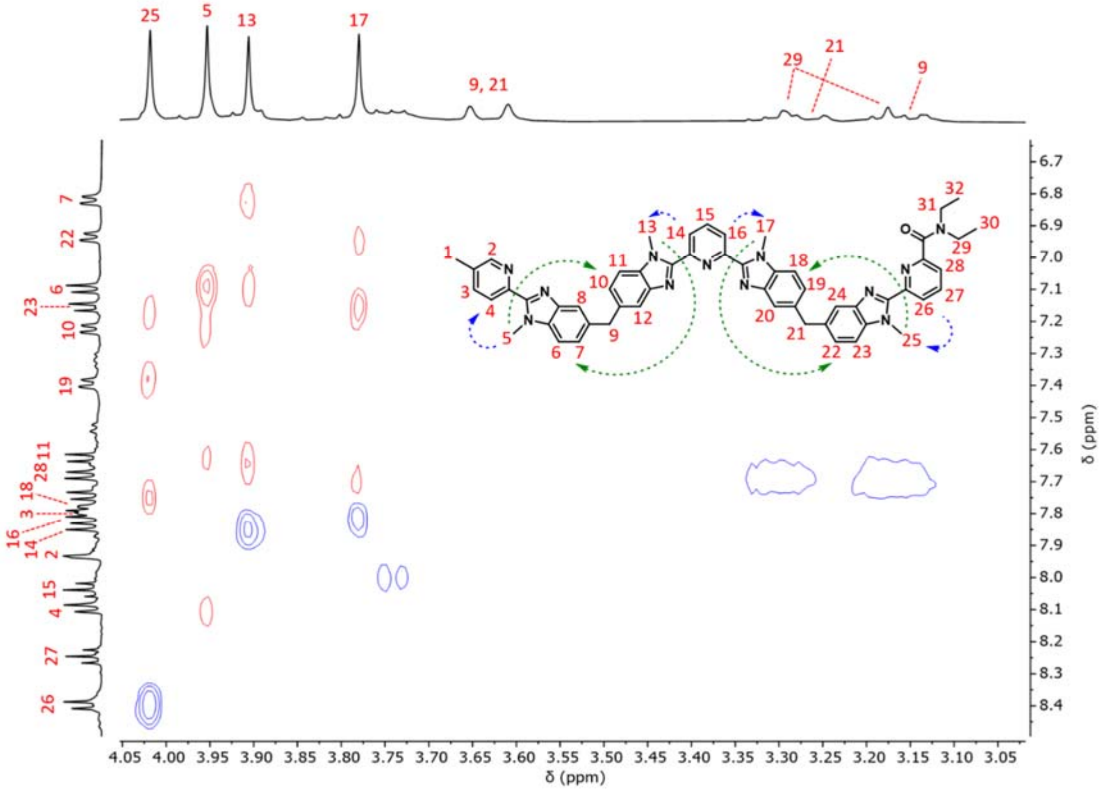

Fig. S13 NOESY (CD_3CN, 400 MHz, 298 K) of the *HHH*-[(**L4**$_3$Zn)La$_2$]$^{8+}$ complex. The relevant NOE correlations confirming the cisoid conformation and representing the interstrand interactions are highlighted on the structure with blue and green arrows respectively.

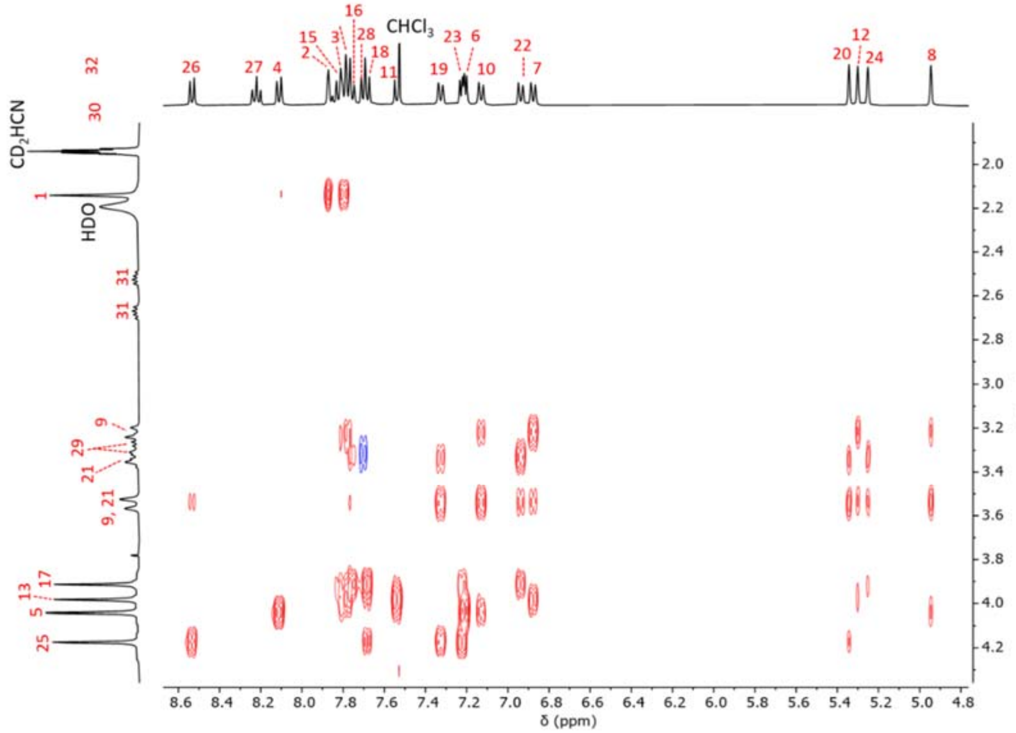

Fig. S14 NOESY (2:1 $CD_3CN/CDCl_3$, 400 MHz, 298 K) of the *HHH*-[(**L4**$_3$Zn)Lu$_2$]$^{8+}$ complex.

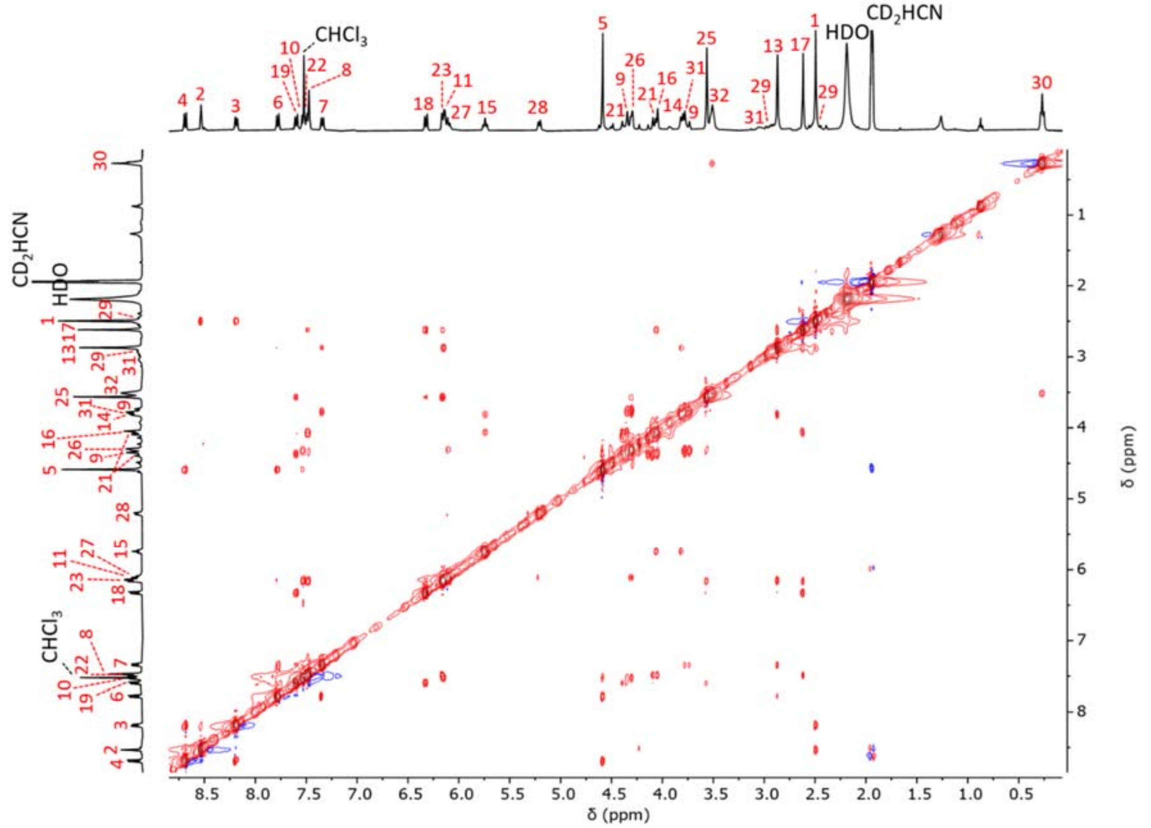

Fig. S15 NOESY (2:1 $CD_3CN/CDCl_3$, 400 MHz, 298 K) of the *HHH*-[(**L4**$_3$Zn)Eu_2]$^{8+}$ complex.

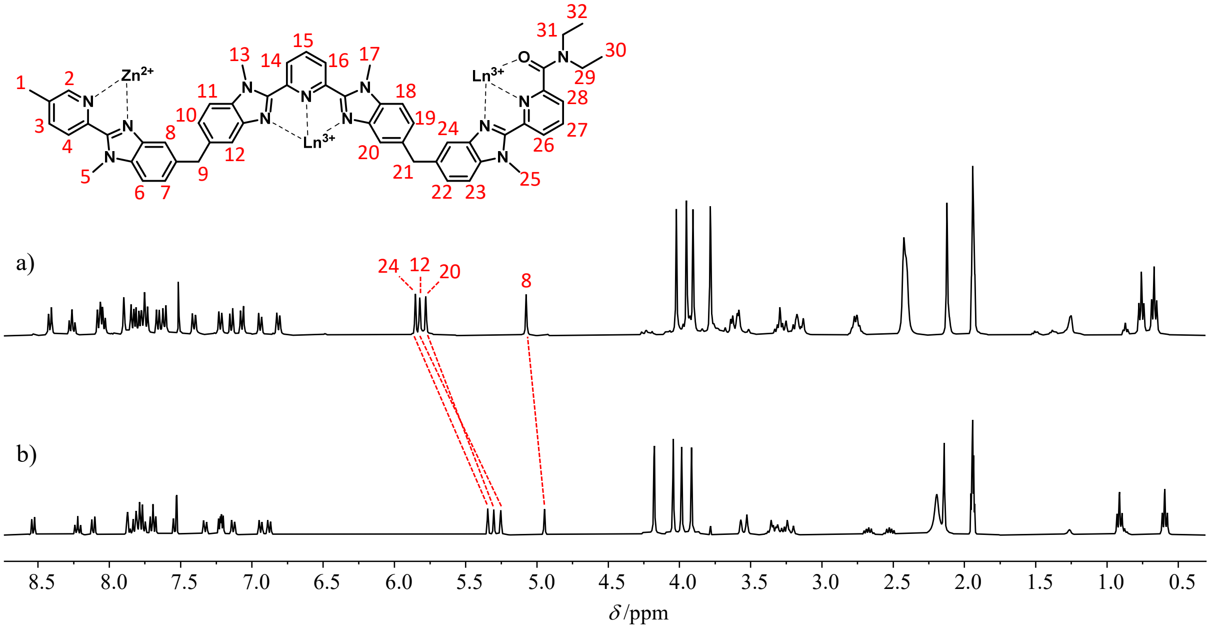

Fig. S16 Comparison of the ^{1}H-NMR spectra (2:1 $CD_3CN/CDCl_3$, 400 MHz, 298 K) of a) *HHH*-[(**L4**$_3$Zn)La_2]$^{8+}$ and b) *HHH*-[(**L4**$_3$Zn)Lu_2]$^{8+}$.

Table S4 Comparison of the ^{1}H-NMR chemical shifts for **L4** ($CDCl_3$), *HHH*-[(**L4**$_3$Zn)La_2]$^{8+}$and *HHH*-[(**L4**$_3$Zn)Lu_2]$^{8+}$ (2:1 $CD_3CN/CDCl_3$).

	Bidentate binding site												
	H^1 (Me)	H^2 (CH)	H^3 (CH)	H^4 (CH)	H^5 (Me)	H^6 (CH)	H^7 (CH)	H^8 (CH)	H^9 (CH_2)				
**L4**	2.40	8.51	7.64	8.25	4.23	7.32	7.21	7.69	4.30				
HHH-[(**L4**$_3$Zn)La_2]$^{8+}$	2.12	7.90	7.76	8.07	3.95	7.07	6.81	5.07	3.61	3.15			
HHH-[(**L4**$_3$Zn)Lu_2]$^{8+}$	2.14	7.87	7.79	8.11	4.04	7.20	6.88	4.95	3.55	3.22			
$\Delta\delta_{La\text{-}Lu}$ (ppm)[a]	-0.02	0.03	-0.03	-0.04	-0.09	-0.13	-0.07	0.12	0.06	-0.07			
	Tridentate binding site (N_9)												
	H^{10} (CH)	H^{11} (CH)	H^{12} (CH)	H^{13} (Me)	H^{14} (CH)	H^{15} (CH)	H^{16} (CH)	H^{17} (Me)	H^{18} (CH)	H^{19} (CH)	H^{20} (CH)	H^{21} (CH_2)	
**L4**	7.25	7.35	7.73	4.20	8.38	8.01	8.38	4.20	7.34	7.24	7.73	4.31	
HHH-[(**L4**$_3$Zn)La_2]$^{8+}$	7.22	7.61	5.78	3.9	7.84	8.05	7.80	3.78	7.74	7.40	5.82	3.61	3.30
HHH-[(**L4**$_3$Zn)Lu_2]$^{8+}$	7.13	7.54	5.3	3.98	7.77	7.81	7.76	3.91	7.68	7.32	5.25	3.55	3.33
$\Delta\delta_{La\text{-}Lu}$ (ppm)[a]	0.09	0.07	0.48	-0.08	0.07	0.24	0.04	-0.13	0.06	0.08	0.57	0.06	-0.03
	Tridentate binding site (N_6O_3)												
	H^{22} (CH)	H^{23} (CH)	H^{24} (CH)	H^{25} (Me)	H^{26} (CH)	H^{27} (CH)	H^{28} (CH)	H^{29} (CH_2)		H^{30} (Me)	H^{31} (CH_2)		H^{32} (Me)
**L4**	7.24	7.33	7.71	4.19	8.39	7.93	7.57	3.61		1.29	3.35		1.12
HHH-[(**L4**$_3$Zn)La_2]$^{8+}$	6.94	7.14	5.85	4.02	8.42	8.26	7.66	3.29	3.16	0.76	2.76	2.76	0.67
HHH-[(**L4**$_3$Zn)Lu_2]$^{8+}$	6.94	7.23	5.34	4.17	8.53	8.22	7.7	3.31	3.25	0.91	2.68	2.52	0.59
$\Delta\delta_{La\text{-}Lu}$ (ppm)[a]	0	-0.09	0.51	-0.15	-0.11	0.04	-0.04	-0.02	-0.09	-0.15	0.08	0.24	0.08

[a] Positive numbers indicate an upfield shift and negative numbers indicate a downfield shift.

Table S5 Comparison of the ^{1}H-NMR chemical shifts for *HHH*-[(**L4**$_3$Zn)La$_2$]$^{8+}$and *HHH*-[(**L4**$_3$Zn)Eu$_2$]$^{8+}$ (2:1 $CD_3CN/CDCl_3$).

Bidentate binding site (N_6)													
	H^{1} (Me)	H^{2} (CH)	H^{3} (CH)	H^{4} (CH)	H^{5} (Me)	H^{6} (CH)	H^{7} (CH)	H^{8} (CH)	H^{9} (CH_2)				
HHH-[(**L4**$_3$Zn)La$_2$]$^{8+}$	2.12	7.90	7.76	8.07	3.95	7.07	6.81	5.07	3.61	3.15			
HHH-[(**L4**$_3$Zn)Eu$_2$]$^{8+}$	2.49	8.53	8.19	8.69	4.59	7.78	7.34	7.47	4.32	3.76			
$\Delta\delta_{La\text{-}Eu}$ (ppm)^{a}	-0.37	-0.63	-0.43	-0.62	-0.64	-0.71	-0.53	-2.40	-0.71	-0.61			
Tridentate binding site (N_9)													
	H^{10} (CH)	H^{11} (CH)	H^{12} (CH)	H^{13} (Me)	H^{14} (CH)	H^{15} (CH)	H^{16} (CH)	H^{17} (Me)	H^{18} (CH)	H^{19} (CH)	H^{20} (CH)	H^{21} (CH_2)	
HHH-[(**L4**$_3$Zn)La$_2$]$^{8+}$	7.22	7.61	5.78	3.90	7.84	8.05	7.80	3.78	7.74	7.40	5.82	3.61	3.30
HHH-[(**L4**$_3$Zn)Eu$_2$]$^{8+}$	7.53	6.14	12.00	2.87	3.81	5.74	4.06	2.62	6.32	7.60	14.62	4.37	4.07
$\Delta\delta_{La\text{-}Eu}$ (ppm)^{a}	-0.31	1.47	-6.22	1.03	4.03	2.31	3.74	1.16	1.42	-0.20	-8.80	-0.76	-0.77
Tridentate binding site (N_6O_3)													
	H^{22} (CH)	H^{23} (CH)	H^{24} (CH)	H^{25} (Me)	H^{26} (CH)	H^{27} (CH)	H^{28} (CH)	H^{29} (CH_2)		H^{30} (Me)	H^{31} (CH_2)		H^{32} (Me)
HHH-[(**L4**$_3$Zn)La$_2$]$^{8+}$	6.94	7.14	5.85	4.02	8.42	8.26	7.66	3.29	3.16	0.76	2.76	2.76	0.67
HHH-[(**L4**$_3$Zn)Eu$_2$]$^{8+}$	7.48	6.16	12.68	3.56	4.30	6.11	5.21	2.92	2.51	0.27	3.80	3.04	3.51
$\Delta\delta_{La\text{-}Eu}$ (ppm)^{a}	-0.54	0.98	-6.83	0.46	4.12	2.15	2.45	0.37	0.65	0.49	-1.04	-0.28	-2.84

^{a} Positive values indicate an upfield shift and negative values indicate a downfield shift.

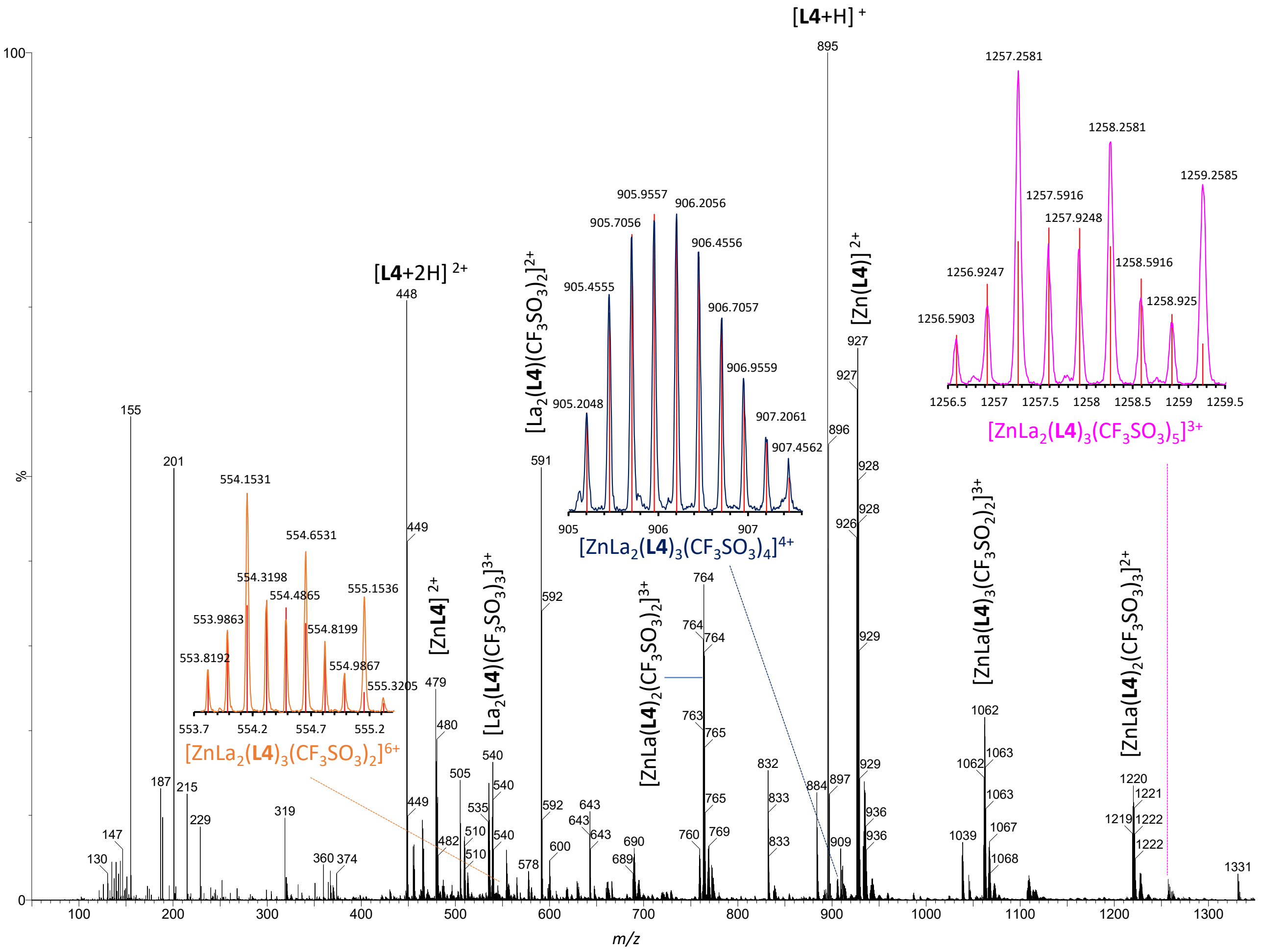

Fig. S17 High-resolution ESI-TOF spectrum of a $8.83 \cdot 10^{-4}$ M solution of *HHH*-$[(\mathbf{L4}_3Zn)La_2]^{8+}$ in acetonitrile highlighting the peaks for $\{[ZnLa_2(\mathbf{L4})_3](CF_3SO_3)_2\}^{6+}$ (*m*/*z* 553-555), $[\mathbf{L4}+2H]^{2+}$ (*m*/*z* 448), $[\mathbf{L4}+H]^{+}$ (*m*/*z* 895), $\{[ZnLa_2(\mathbf{L4})_3](CF_3SO_3)_4\}^{4+}$ (*m*/*z* 905-907), and $\{[ZnLa_2(\mathbf{L4})_3](CF_3SO_3)_5\}^{3+}$ (*m*/*z* 1256-1259).

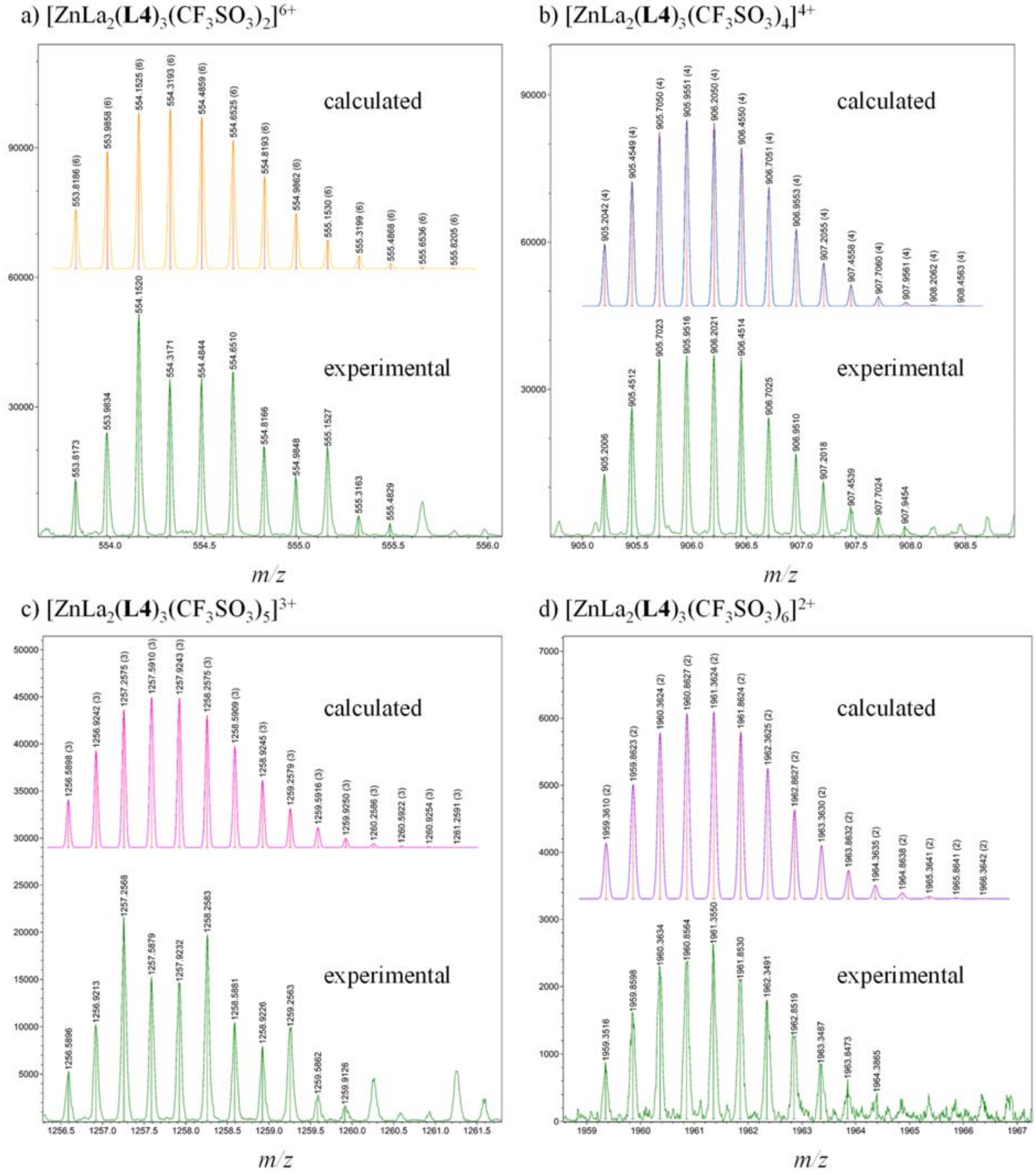

Fig. S18 Comparison of the experimental and theoretical isotopic patterns for $\{[ZnLa_2(\mathbf{L4})_3](CF_3SO_3)_n\}^{(8-n)+}$ (n = 2, 4, 5, 6) adducts observed in the high-resolution ESI-TOF spectrum of a $8.83 \cdot 10^{-4}$ M solution of *HHH*-$[(\mathbf{L4}_3Zn)La_2]^{8+}$ in acetonitrile.

Table S6 List of the peaks detected in the ESI-TOF spectrum of a $8.83 \cdot 10^{-4}$ M solution of *HHH*-$[(\mathbf{L4}_3Zn)La_2]^{8+}$ in acetonitrile. The ions were identified by interpretation of the isotopic pattern. The peaks corresponding to the series of triflate adducts $\{[ZnLa_2(\mathbf{L4})_3](CF_3SO_3)_n\}^{(8-n)+}$ (n = 2, 4, 5, 6) are highlighted in pink.

Ion	Exp *m/z*	Theo *m/z*	Δ *m/z*
$[\mathbf{L4}+2H]^{2+}$	448.2201	448.2194	0.0007
$[Zn(\mathbf{L4})]^{2+}$	479.1763	479.1761	0.0002
$\{[La_2(\mathbf{L4})](CF_3SO_3)_3\}^{3+}$	539.6931	539.6973	0.0042
$\{[ZnLa_2(\mathbf{L4})_3](CF_3SO_3)_2\}^{6+}$	553.8160	553.8192	0.0032
$\{[Zn(\mathbf{L4}+H)](CF_3SO_3)\}^{2+}$	554.1531	554.1560	0.0029
$\{[La(\mathbf{L4})](CF_3SO_3)\}^{2+}$	591.1444	591.1407	0.0037
$[La(\mathbf{L4})_2]^{3+}$	642.5811	642.5842	0.0031
$\{ZnLa(\mathbf{L4})_2](CF_3SO_3)_2\}^{3+}$	763.1931	763.1952	0.0021
$\{[La_2(\mathbf{L4})](CF_3SO_3)_4\}^{4+}$	884.0204	884.0219	0.0015
$[\mathbf{L4}+H]^{+}$	895.4270	895.4309	0.0039
$\{[ZnLa_2(\mathbf{L4})_3](CF_3SO_3)_4\}^{4+}$	905.2007	905.2048	0.0041
$[Zn(\mathbf{L4})_2]^{2+}$	926.3889	926.3876	0.0013
$\{[La(\mathbf{L4})_2](CF_3SO_3)\}^{2+}$	1038.3521	1038.3523	0.0002
$\{[ZnLa(\mathbf{L4})_3](CF_3SO_3)_2\}^{3+}$	1061.3363	1061.3362	0.0001
$\{[ZnLa(\mathbf{L4})_2](CF_3SO_3)_3\}^{2+}$	1219.2698	1219.2688	0.0010
$\{[ZnLa_2(\mathbf{L4})_3](CF_3SO_3)_5\}^{3+}$	1256.5880	1256.5903	0.0023
$\{[Zn(\mathbf{L4}+H)](CF_3SO_3)\}^{+}$	1257.2626	1257.2640	0.0014
$\{[La(\mathbf{L4})](CF_3SO_3)_2\}^{+}$	1331.2318	1331.2334	0.0016
$\{[ZnLa_2(\mathbf{L4})_3](CF_3SO_3)_6\}^{2+}$	1959.3546	1959.3616	0.0070

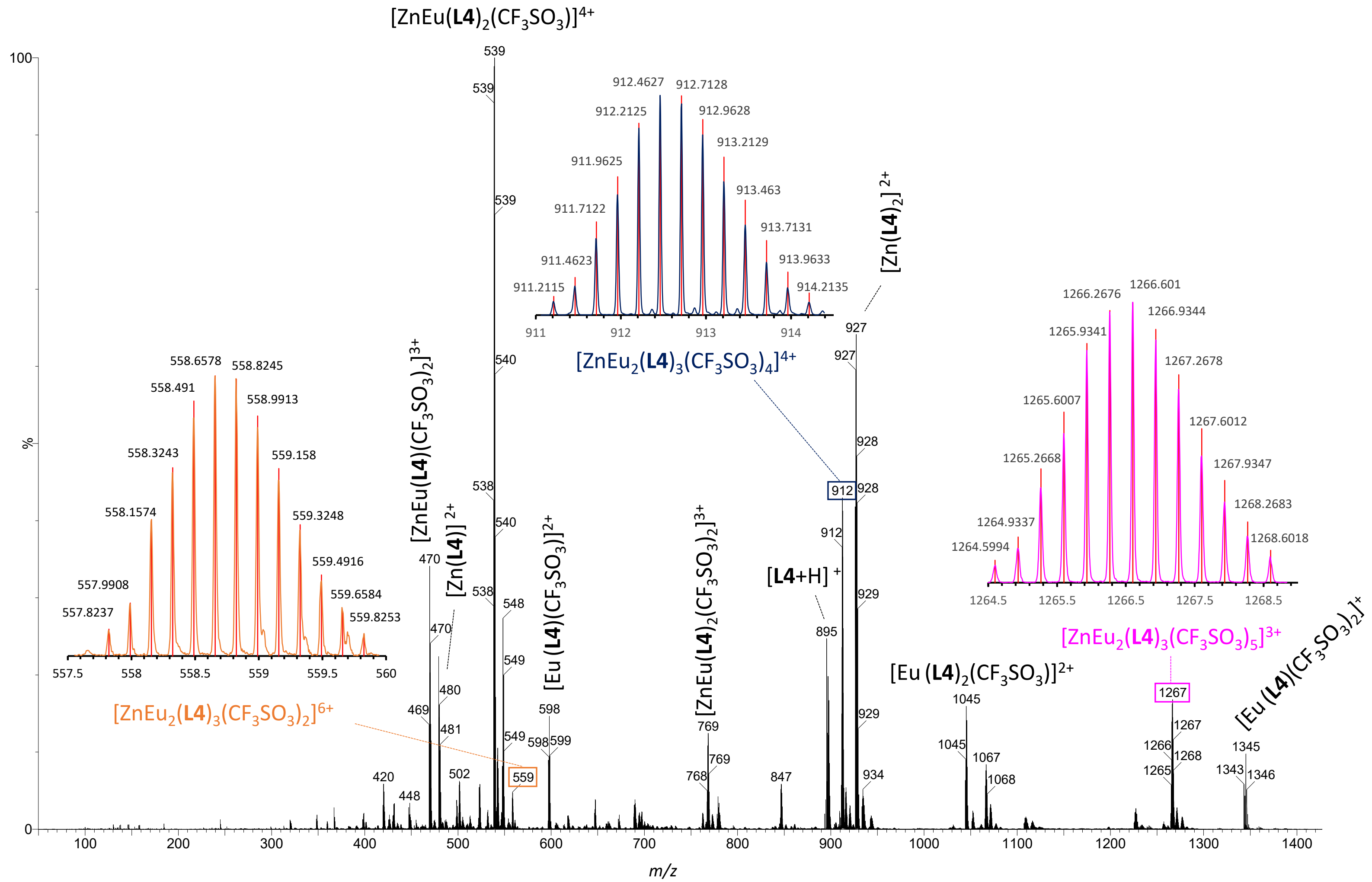

Fig. S19 High-resolution ESI-TOF spectrum of a $9.87 \cdot 10^{-4}$ M solution of the *HHH*-$[ZnEu_2(\mathbf{L4})_3]^{8+}$ complex in acetonitrile highlighting the peaks for $\{[ZnEu_2(\mathbf{L4})_3](CF_3SO_3)_2\}^{6+}$ (*m/z* 553-555), $[\mathbf{L4}+2H]^{2+}$ (*m/z* 448), $[\mathbf{L4}+H]^{+}$ (*m/z* 895), $\{[ZnEu_2(\mathbf{L4})_3](CF_3SO_3)_4\}^{4+}$ (*m/z* 905-907), and $\{[ZnEu_2(\mathbf{L4})_3](CF_3SO_3)_5\}^{3+}$ (*m/z* 1256-1259).

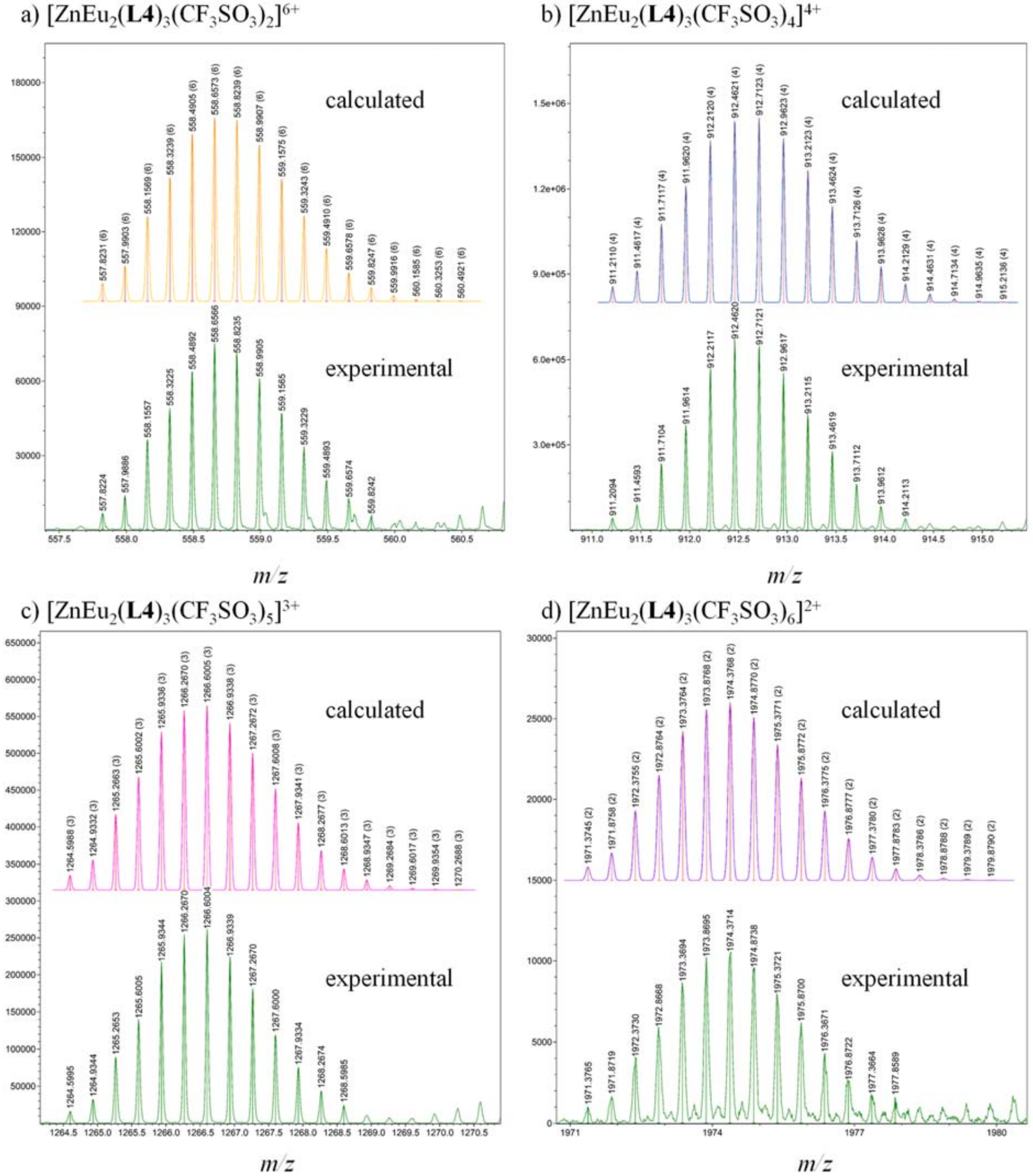

Fig. S20 Comparison of the experimental and theoretical isotopic patterns for $\{[ZnEu_2(\mathbf{L4})_3](CF_3SO_3)_n\}^{(8-n)+}$ (n = 2, 4, 5, 6) adducts observed in the high-resolution ESI-TOF spectrum of a $9.87 \cdot 10^{-4}$ M solution of *HHH*-$[(\mathbf{L4}_3Zn)Eu_2]^{8+}$ in acetonitrile.

Table S7 List of the peaks detected in the ESI-TOF spectrum of a $9.87\cdot10^{-4}$ M solution of the *HHH*-$[(\mathbf{L4}_3Zn)Eu_2]^{8+}$ in acetonitrile. The ions were identified by interpretation of the isotopic pattern. The peaks corresponding to the series of triflate adducts $\{[ZnEu_2(\mathbf{L4})_3](CF_3SO_3)_n\}^{(8-n)+}$ (n = 2, 4, 5, 6) are highlighted in orange.

Ion	Exp m/z	Theo m/z	Δ m/z
$[\mathbf{L4}+2H]^{2+}$	448.2157	448.2194	0.0037
$\{[ZnEu(\mathbf{L4})](CF_3SO_3)_2\}^{3+}$	469.0587	469.0587	0.0000
$[Zn(\mathbf{L4})]^{2+}$	479.1763	479.1761	0.0002
$\{[ZnEu(\mathbf{L4})_2](CF_3SO_3)\}^{4+}$	538.1636	538.1618	0.0018
$\{[Eu_2(\mathbf{L4})](CF_3SO_3)_3\}^{3+}$	547.7034	547.7063	0.0029
$\{[ZnEu_2(\mathbf{L4})_3](CF_3SO_3)_2\}^{6+}$	557.8187	557.8237	0.0050
$\{[Eu(\mathbf{L4})](CF_3SO_3)\}^{2+}$	597.1442	597.1475	0.0033
$\{[ZnEu(\mathbf{L4})_2](CF_3SO_3)_2\}^{3+}$	767.1946	767.1997	0.0051
$\{[Eu_2(\mathbf{L4})_2](CF_3SO_3)_3\}^{3+}$	845.8421	845.8473	0.0052
$[\mathbf{L4}+H]^{+}$	895.4331	895.4309	0.0022
$\{[ZnEu_2(\mathbf{L4})_3](CF_3SO_3)_4\}^{4+}$	911.2065	911.2115	0.0050
$[Zn(\mathbf{L4})_2]^{2+}$	926.3826	926.3876	0.0050
$\{[Eu(\mathbf{L4})_2](CF_3SO_3)\}^{2+}$	1044.3539	1044.359	0.0051
$\{[ZnEu_2(\mathbf{L4})_3](CF_3SO_3)_5\}^{3+}$	1264.6008	1264.5994	0.0014
$\{[Eu(\mathbf{L4})](CF_3SO_3)_2\}^{+}$	1343.2556	1343.2469	0.0087
$\{[ZnEu_2(\mathbf{L4})_3](CF_3SO_3)_6\}^{2+}$	1971.3734	1971.3751	0.0017

Table S8 Crystal data and structure refinement for [(**L4**$_3$Zn)Eu$_2$](CF$_3$SO$_3$)$_8$·12(C$_3$H$_8$O) and [(**L4**$_3$Zn)La$_2$](CF$_3$SO$_3$)$_8$·7.25(CH$_3$CN).

	[(**L4**$_3$Zn)Eu$_2$] (CF$_3$SO$_3$)$_8$· 12(C$_3$H$_8$O)	[(**L4**$_3$Zn)La$_2$] (CF$_3$SO$_3$)$_8$· 7.25(CH$_3$CN)
Empirical formula	$C_{175}H_{155}Eu_2F_{21}N_{36}O_{25}S_7Zn$	$C_{187.75}H_{171.75}F_{24}La_2N_{43.25}O_{27}S_8Zn$
Chemical formula moiety	$C_{165}H_{148}Eu_2N_{36}O_3Zn$ ·$(CF_3SO_3)_8$·$(C_3H_8O)_{12}$	$C_{165}H_{150}La_2N_{36}O_3Zn$· $(CF_3SO_3)_8$·$(CH_3CN)_{7.25}$
Formula weight	4155.05	4521.59
Temperature	119.99(10) K	99.99(10) K
Wavelength	1.54184 Å	1.54184 Å
Crystal system	Triclinic	Monoclinic
Space group	*P* -1	*I* 2/*a*
Unit cell dimensions	*a* = 16.5519(3) Å	*a* = 27.68761(13) Å
	b = 17.2409(3) Å	*b* = 50.6972(3) Å
	c = 48.1977(9) Å	*c* = 30.80194(17) Å
	α = 92.6755(14)°	α = 90°
	β = 95.8386(14)°	β = 104.6438(5)°
	γ = 118.0562(18)°	γ = 90°
Volume	12004.7(4) Å^3	41831.7(4) Å^3
Z	2	8
Density (calculated)	1.149 Mg.m^{-3}	1.436 Mg.m^{-3}
Absorption coefficient	5.018 mm^{-1}	4.806 mm^{-1}
F(000)	4228	18440
Crystal size	0.251 x 0.122 x 0.041 mm^3	0.24 x 0.21 x 0.15 mm^3
Theta range for data collection	2.781 to 73.236 °	2.592 to 72.743 °
Index ranges	$-20 \leq h \leq 17$, $-21 \leq k \leq 20$, $-58 \leq l \leq 58$	$-34 \leq h \leq 25$, $-62 \leq k \leq 62$, $-30 \leq l \leq 37$
Reflections collected	85991	185218
Independent reflections	40878 [R(int) = 0.0536]	41060 [R(int) = 0.0253]
Completeness to theta = 72.000°	86.40%	99.80%
Absorption correction	Analytical	Analytical
Max. and min. transmission	0.812 and 0.452	0.575 and 0.444
Refinement method	Full-matrix least-squares on F^2	Full-matrix least-squares on F^2
Data / restraints / parameters	40878 / 114 / 2244	41060 / 189 / 2715
Goodness-of-fit on F^2	1.167	1.048
Final R indices [I>2sigma(I)]	R_1 = 0.1086, wR_2 = 0.3049	R_1 = 0.0669, wR_2 = 0.1996
R indices (all data)	R_1 = 0.1352, wR_2 = 0.3255	R_1 = 0.0722, wR_2 = 0.2058
Largest diff. peak and hole	1.732 and -1.328 e.Å^{-3}	2.779 and -0.999 e.Å^{-3}

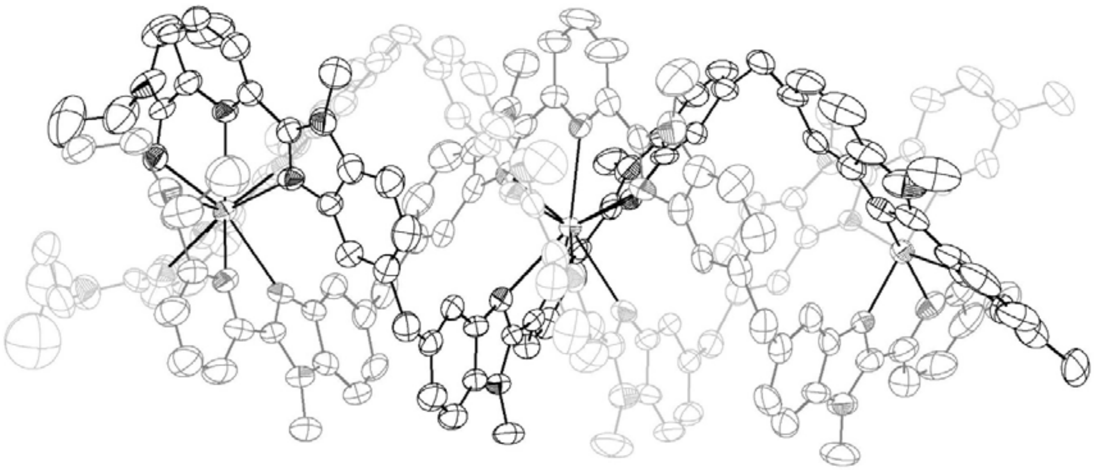

Fig. S21 ORTEP view of *HHH*-$[(\mathbf{L4}_3Zn)Eu_2]^{8+}$ (thermal ellipsoids are drawn at 40% probability level). Hydrogen atoms, counter ions and solvent molecules were omitted for clarity purpose. Each ligand strands were drawn with different grey scale for clarity reason.

Table S9 Selected bond lengths (Å) for $[(\mathbf{L4}_3Zn)Eu_2](CF_3SO_3)_8{\cdot}12(C_3H_8O)$.

	Ligand A	Ligand B	Ligand C
Zn(1)-N(1)	2.197(7)	2.173(6)	2.211(7)
Zn(1)-N(2)	2.087(8)	2.117(6)	2.112(6)
Eu(1)-N(4)	2.616(7)	2.573(7)	2.606(7)
Eu(1)-N(6)	2.615(7)	2.601(7)	2.611(7)
Eu(1)-N(7)	2.581(6)	2.587(8)	2.538(6)
Eu(2)-O(1)	2.428(6)	2.350(6)	2.388(6)
Eu(2)-N(9)	2.585(7)	2.559(7)	2.589(6)
Eu(2)-N(11)	2.624(6)	2.580(8)	2.582(7)

Table S10 Selected bond angles (°) for $[(\mathbf{L4}_3Zn)Eu_2](CF_3SO_3)_8{\cdot}12(C_3H_8O)$.

N(4C)-Eu(1)-N(4)	85.3(2)	O(1B)-Eu(2)-N(9B)	126.0(2)
N(4C)-Eu(1)-N(6C)	62.0(2)	O(1B)-Eu(2)-N(11C)	67.7(2)
N(4C)-Eu(1)-N(6)	140.0(2)	O(1B)-Eu(2)-N(11)	136.0(2)
N(4B)-Eu(1)-N(4)	86.2(2)	O(1B)-Eu(2)-N(11B)	63.0(2)
N(4B)-Eu(1)-N(4C)	85.2(2)	O(1B)-Eu(2)-C(46)	99.9(2)
N(4B)-Eu(1)-N(6B)	62.4(3)	O(1B)-Eu(2)-C(46B)	18.1(2)
N(4B)-Eu(1)-N(6C)	141.5(2)	O(1B)-Eu(2)-C(46C)	80.0(2)
N(4B)-Eu(1)-N(6)	72.5(2)	N(9)-Eu(2)-N(9C)	90.3(2)

N(4B)-Eu(1)-N(7B)	125.0(2)	N(9)-Eu(2)-N(11)	61.7(2)
N(4B)-Eu(1)-N(7)	76.4(2)	N(9)-Eu(2)-C(46)	107.3(2)
N(6B)-Eu(1)-N(4)	140.6(2)	N(9)-Eu(2)-C(46B)	159.4(2)
N(6B)-Eu(1)-N(4C)	69.9(2)	N(9)-Eu(2)-C(46C)	69.5(2)
N(6B)-Eu(1)-N(6C)	117.6(3)	N(9C)-Eu(2)-N(11)	146.0(2)
N(6B)-Eu(1)-N(6)	122.8(2)	N(9C)-Eu(2)-C(46)	151.1(2)
N(6C)-Eu(1)-N(4)	72.2(2)	N(9C)-Eu(2)-C(46B)	82.3(2)
N(6C)-Eu(1)-N(6)	119.6(2)	N(9C)-Eu(2)-C(46C)	109.7(2)
N(6)-Eu(1)-N(4)	61.1(2)	N(9B)-Eu(2)-N(9)	89.0(2)
N(7B)-Eu(1)-N(4)	144.0(2)	N(9B)-Eu(2)-N(9C)	85.7(2)
N(7B)-Eu(1)-N(4C)	80.6(2)	N(9B)-Eu(2)-N(11C)	143.6(2)
N(7B)-Eu(1)-N(6B)	62.8(2)	N(9B)-Eu(2)-N(11)	75.5(2)
N(7B)-Eu(1)-N(6C)	71.9(2)	N(9B)-Eu(2)-N(11B)	63.2(3)
N(7B)-Eu(1)-N(6)	139.3(2)	N(9B)-Eu(2)-C(46)	72.2(2)
N(7)-Eu(1)-N(4)	123.8(2)	N(9B)-Eu(2)-C(46B)	109.5(3)
N(7)-Eu(1)-N(4C)	143.6(2)	N(9B)-Eu(2)-C(46C)	153.0(3)
N(7)-Eu(1)-N(6B)	73.7(2)	N(11C)-Eu(2)-N(9)	74.0(2)
N(7)-Eu(1)-N(6C)	142.1(2)	N(11C)-Eu(2)-N(9C)	63.0(2)
N(7)-Eu(1)-N(6)	62.7(2)	N(11C)-Eu(2)-N(11)	120.2(2)
N(7)-Eu(1)-N(7B)	84.5(2)	N(11C)-Eu(2)-C(46)	143.3(2)
N(7C)-Eu(1)-N(4)	80.4(2)	N(11C)-Eu(2)-C(46B)	85.5(3)
N(7C)-Eu(1)-N(4C)	124.9(2)	N(11C)-Eu(2)-C(46C)	46.8(2)
N(7C)-Eu(1)-N(4B)	145.3(2)	N(11)-Eu(2)-C(46)	45.7(2)
N(7C)-Eu(1)-N(6B)	138.9(2)	N(11)-Eu(2)-C(46B)	130.6(2)
N(7C)-Eu(1)-N(6C)	62.9(2)	N(11)-Eu(2)-C(46C)	79.8(2)
N(7C)-Eu(1)-N(6)	73.0(2)	N(11B)-Eu(2)-N(9)	149.6(2)
N(7C)-Eu(1)-N(7B)	81.0(2)	N(11B)-Eu(2)-N(9C)	76.4(2)
N(7C)-Eu(1)-N(7)	84.7(2)	N(11B)-Eu(2)-N(11C)	120.9(3)
O(1)-Eu(2)-N(9)	124.66(19)	N(11B)-Eu(2)-N(11)	117.5(2)
O(1)-Eu(2)-N(9C)	143.1(2)	N(11B)-Eu(2)-C(46)	77.0(2)
O(1)-Eu(2)-N(9B)	83.7(2)	N(11B)-Eu(2)-C(46B)	46.4(3)
O(1)-Eu(2)-N(11C)	132.4(2)	N(11B)-Eu(2)-C(46C)	140.7(3)
O(1)-Eu(2)-N(11)	63.3(2)	C(46B)-Eu(2)-C(46)	87.7(2)
O(1)-Eu(2)-N(11B)	67.3(2)	C(46B)-Eu(2)-C(46C)	94.9(3)

O(1)-Eu(2)-C(46)	19.8(2)	C(46C)-Eu(2)-C(46)	98.1(2)
O(1)-Eu(2)-C(46B)	68.4(2)	N(1)-Zn(1)-N(1C)	93.9(3)
O(1)-Eu(2)-C(46C)	95.0(2)	N(1B)-Zn(1)-N(1C)	96.6(3)
O(1C)-Eu(2)-O(1)	75.9(2)	N(1B)-Zn(1)-N(1)	95.6(2)
O(1C)-Eu(2)-N(9)	78.4(2)	N(2)-Zn(1)-N(1C)	169.9(3)
O(1C)-Eu(2)-N(9C)	128.2(2)	N(2)-Zn(1)-N(1)	77.2(3)
O(1C)-Eu(2)-N(9B)	143.2(2)	N(2)-Zn(1)-N(1B)	88.9(3)
O(1C)-Eu(2)-N(11C)	65.2(2)	N(2)-Zn(1)-N(2C)	99.3(3)
O(1C)-Eu(2)-N(11)	68.1(2)	N(2)-Zn(1)-N(2B)	101.4(3)
O(1C)-Eu(2)-N(11B)	131.2(2)	N(2C)-Zn(1)-N(1C)	75.8(3)
O(1C)-Eu(2)-C(46)	78.9(2)	N(2C)-Zn(1)-N(1)	89.9(2)
O(1C)-Eu(2)-C(46B)	91.1(2)	N(2C)-Zn(1)-N(1B)	170.9(3)
O(1C)-Eu(2)-C(46C)	19.5(2)	N(2C)-Zn(1)-N(2B)	98.7(2)
O(1B)-Eu(2)-O(1)	80.1(2)	N(2B)-Zn(1)-N(1C)	88.1(2)
O(1B)-Eu(2)-O(1C)	80.5(2)	N(2B)-Zn(1)-N(1)	171.4(2)
O(1B)-Eu(2)-N(9)	141.3(2)	N(2B)-Zn(1)-N(1B)	75.8(2)
O(1B)-Eu(2)-N(9C)	77.9(2)		

Table S11 Selected intra-ligand least-squares planes data of complex [(**L4**$_3$Zn)Eu$_2$](CF$_3$SO$_3$)$_8$·12(C$_3$H$_8$O).

	Abbreviation	RMSD (Å)	Max deviation (Å) (Atom)
Ligand A			
N1 C5 C4 C3 C2 C1	Py1A	0.014	0.020 (C5)
C8 C9 C10 C11 C12 N3 C6 N2 C7	BZ1A	0.033	0.056 (C9)
N4 C17 N5 C18 C19 C20 C14 C15 C16	BZ2A	0.013	0.023 (C17)
N6 C21 C22 C23 C24 C25	Py2A	0.003	0.005 (C23, C22)
C29 C28 C27 N7 C26 N8 C32 C31 C30	BZ3A	0.028	0.049 (N7)
N9 C37 N10 C38 C39 C40 C34 C36 C35	BZ4A	0.016	0.031 (C34)
N11 C45 C44 C43 C42 C41	Py3A	0.020	0.030 (C42)
Ligand B			
N1B C1B C2B C3B C4B C5B	Py1B	0.021	0.027 (C2B)
N2B C6B N3B C12B C11B C10B C9B C8B C7B	BZ1B	0.032	0.054 (C6B)

C17B N4B C16B C15B C14B C20B C19B C18B N5B	BZ2B	0.014	0.022 (C15B)
N6B C21B C22B C23B C24B C25B	Py2B	0.016	0.027 (C24B)
C26B N8B C32B C31B C30B C29B C28B C27B N7B	BZ3B	0.031	0.056 (N7B)
C35B C34B C40B C39B C38B N10B C37B N9B C36B	BZ4B	0.012	0.018 (C34B)
N11B C41B C42B C43B C44B C45B	Py3B	0.021	0.036 (N11B)
Ligand C			
N1C C5C C4C C3C C2C C1C	Py1C	0.016	0.024 (C2C)
N2C C6C N3C C12C C11C C10C C9C C8C C7C	BZ1C	0.023	0.045 (C12C)
N4C C16C C15C C14C C20C C19C C18C N5C C17C	BZ2C	0.028	0.059 (C19C)
N6C C21C C22C C23C C24C C25C	Py2C	0.021	0.035 (C21C)
N7C C26C N8C C32C C31C C30C C29C C28C C27C	BZ3C	0.021	0.039 (N7C)
N9C C37C N10C C38C C39C C40C C34C C35C C36C	BZ4C	0.025	0.034 (C35C)
N11C C41C C42C C43C C44C C45C	Py3C	0.004	0.006 (C43C)

Table S12 Intra-ligand interplanar angles *α* (°) for [(**L4**$_3$Zn)Eu$_2$](CF$_3$SO$_3$)$_8$·12(C$_3$H$_8$O).

	BZ1A	BZ2A	Py2A	BZ3A	BZ4A	Py3A
Py1A	160.2(4)	85.2(4)	77.7(4)	118.8(4)	44.3(4)	32.6(4)
BZ1A	0	106.3(2)	105.8(3)	67.0(3)	155.4(3)	167.1(4)
BZ2A		0	24.8(3)	128.2(2)	58.4(2)	69.0(3)
Py2A			0	151.7(3)	68.8(3)	75.1(4)
BZ3A				0	105.83(19)	105.9(3)
BZ4A					0	13.0(3)
Py3A						0

	BZ1B	BZ2B	Py2B	BZ3B	BZ4B	Py3B
Py1B	14.0(2)	90.5(2)	82.7(4)	60.5(3)	128.6(3)	111.7(4)

BZ1B	0	111.7(4)	74.8(3)	59.1(2)	142.5(3)	125.4(4)
BZ2B		0	162.0(4)	130.6(3)	56.5(2)	52.3(4)
Py2B			0	32.6(4)	116.1(3)	64.9(4)
BZ3B				0	109.6(2)	80.5(4)
BZ4B					0	162.9(4)
Py3B						0

	BZ1C	BZ2C	Py2C	BZ3C	BZ4C	Py3C
Py1C	11.7(4)	97.1(4)	110.9(5)	130.6(4)	46.8(4)	36.1(5)
BZ1C	0	98.0(2)	110.5(3)	124.4(2)	35.4(3)	24.4(4)
BZ2C		0	14.7(4)	44.9(2)	106.9(2)	98.5(4)
Py2C			0	31.0(4)	112.2(3)	106.4(4)
BZ3C				0	106.52(18)	108.1(4)
BZ4C					0	13.3(4)
Py3C						0

Table S13 Selected least-squares chelate planes data of complex $[(\mathbf{L4}_3Zn)Eu_2](CF_3SO_3)_8\cdot 12(C_3H_8O)$.

	Abbreviation	RMSD (Å)	Max deviation (Å) (Atom)
Terminal tridentate site			
Eu2 O1 C46 C45 N11	Ch1A	0.138	0.220 (C46)
Eu2 N11 C41 C37 N9	Ch2A	0.088	0.125 (N11)
Eu2 N11B C45B C46B O1B	Ch1B	0.107	0.69 (C46B)
Eu2 N9B C37B C41B N11B	Ch2B	0.054	0.082 (C37B)
Eu2 O1C C46C C45C N11C	Ch1C	0.111	0.175 (C46C)
Eu2 N11C C41C C37C N9C	Ch2C	0.114	0.164 (N11C)
Central tridentate site			
Eu1 N4 C17 C21 N6	Ch3A	0.154	0.217 (N4)
Eu1 N6 C25 C26 N7	Ch4A	0.162	0.231 (N7)
Eu1 N6B C21B C17B N4B	Ch3B	0.129	0.182 (N4B)
Eu1 N7B C26B C25B N6B	Ch4B	0.171	0.246 (N7B)
Eu1 N6C C21C C17C N4C	Ch3C	0.115	0.153 (N4C)
Eu1 N7C C26C C25C N6C	Ch4C	0.142	0.205 (N7C)

Didentate site			
Zn1 N1 C5 C6 N2	Ch5A	0.063	0.097 (C5)
Zn1 N2B C6B C5B N1B	Ch5B	0.057	0.083 (C5B)
Zn1 N1C C5C C6C N2C	Ch5C	0.065	0.093 (N1C)

Table S14 Interplanar chelate angles β (°) for $[(\mathbf{L4}_3Zn)Eu_2](CF_3SO_3)_8{\cdot}12(C_3H_8O)$.

	Ch2A	Ch1B	Ch2B	Ch1C	Ch2C
Ch1A	6.1(2)	73.9(3)	73.3(2)	73.9(3)	77.1(3)
Ch2A	0	76.7(3)	75.9(3)	68.8(2)	72.3(3)
Ch1B		0	2.5(3)	79.0(3)	75.3(2)
Ch2B			0	76.4(3)	72.8(3)
Ch1C				0	5.6(2)
Ch2C					0

	Ch4A	Ch3B	Ch4B	Ch3C	Ch4C
Ch3A	11.1(2)	68.0(3)	57.9(9)	68.3(3)	62.6(2)
Ch4A	0	59.8(2)	68.7(3)	58.9(3)	68.9(3)
Ch3B		0	10.6(2)	66.9(3)	72.2(3)
Ch4B			0	62.2(2)	68.8(2)
Ch3C				0	9.1(2)
Ch4C					0

	Ch5A	Ch5B	Ch5C
Ch5A	0	85.2(3)	88.2(3)
Ch5B		0	84.8(3)
Ch5C			0

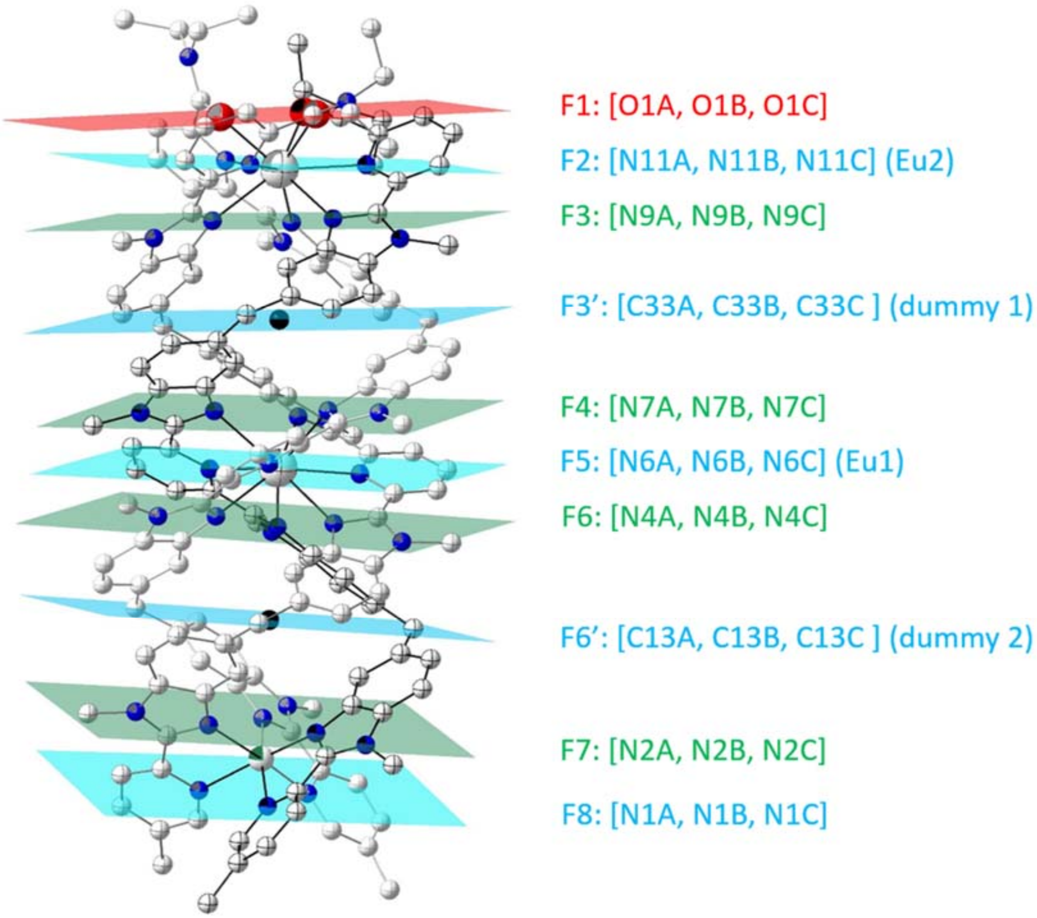

Fig. S22. Helical portions F*i*-F*j* in the molecular structure of *HHH*-$[(\mathbf{L4}_3Zn)Eu_2]^{8+}$.

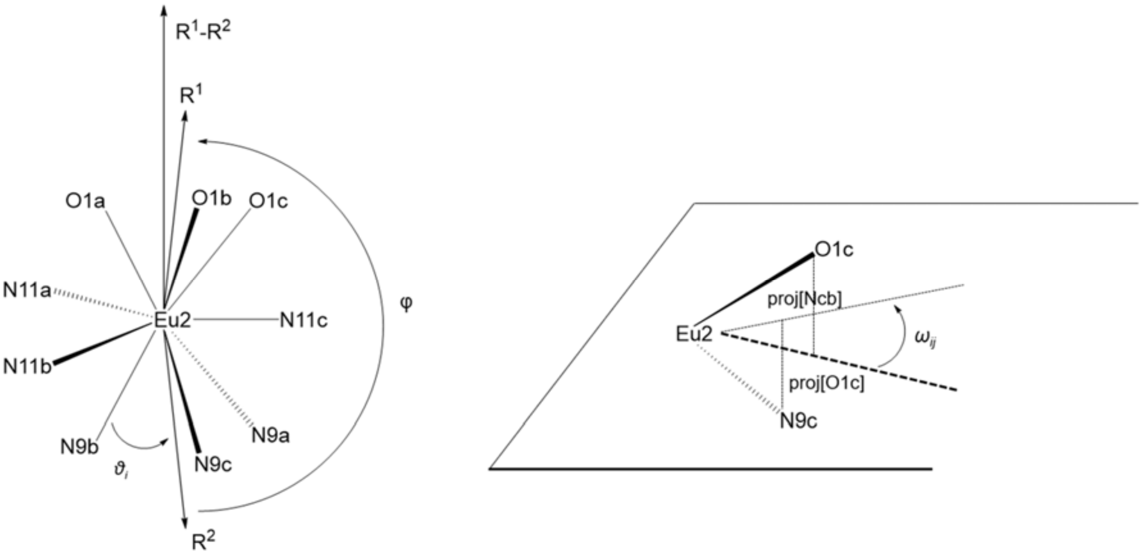

Fig. S23 Definitions of angles φ, θ_i, and ω_{ij} for Eu2 coordination sphere in *HHH*-$[(\mathbf{L4}_3Zn)Eu_2]^{8+}$.

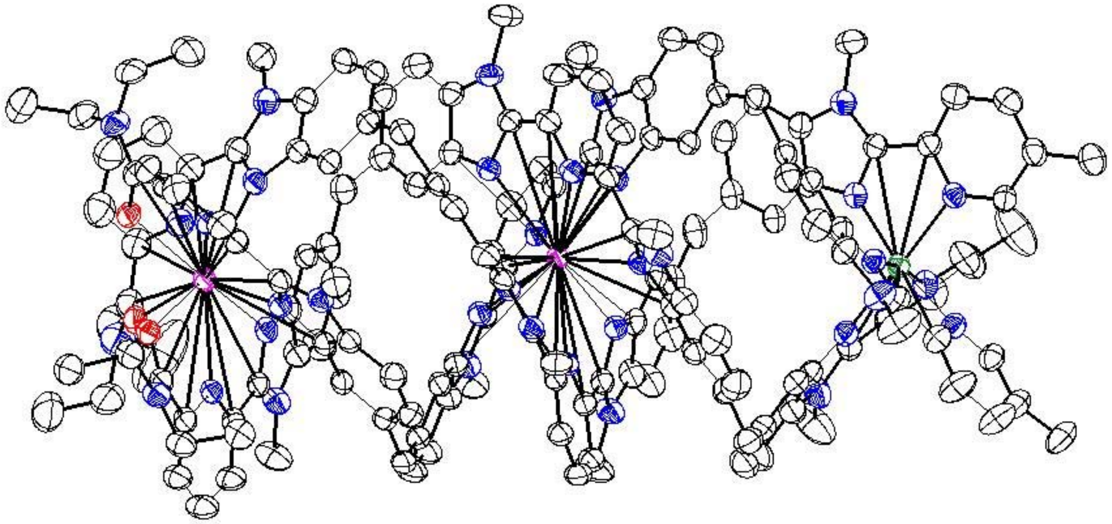

Fig. S24 ORTEP view of *HHH*-$[(\mathbf{L4}_3Zn)La_2]^{8+}$ (thermal ellipsoids are drawn at 50% probability). Hydrogen atoms, triflates counter ions and acetonitrile solvent molecules are omitted for clarity purposes.

Table S15 Selected bond lengths (Å) for $[(\mathbf{L4}_3Zn)La_2](CF_3SO_3)_8\cdot 7.25(CH_3CN)$

	Ligand A	Ligand B	Ligand C
Zn(1)-N(1)	2.220(4)	2.194(4)	2.211(4)
Zn(1)-N(2)	2.147(4)	2.130(4)	2.150(4)
La(2)-N(4)	2.692(4)	2.657(3)	2.650(3)
La(2)-N(6)	2.735(3)	2.710(4)	2.704(4)
La(2)-N(7)	2.707(4)	2.645(4)	2.670(3)
La(1)-O(1)	2.428(3)	2.475(3)	2.450(3)
La(1)-N(9)	2.692(3)	2.714(4)	2.679(4)
La(1)-N(11)	2.684(4)	2.750(4)	2.728(4)

Table S16 Selected bond angles (°) for [(**L4**$_3$Zn)La$_2$](CF$_3$SO$_3$)$_8$·7.25(CH$_3$CN)

N(4)-La(2)-N(6C)	143.57(10)	O(1B)-La(1)-N(9B)	121.11(12)
N(4)-La(2)-N(6B)	71.93(11)	O(1B)-La(1)-N(9C)	144.00(11)
N(4)-La(2)-N(6)	60.74(11)	O(1B)-La(1)-N(11)	69.36(11)
N(4)-La(2)-N(7)	120.86(11)	O(1B)-La(1)-N(11B)	61.36(12)
N(4C)-La(2)-N(4)	87.49(11)	O(1B)-La(1)-N(11C)	139.44(11)
N(4C)-La(2)-N(4B)	85.46(11)	O(1)-La(1)-O(1C)	78.88(10)
N(4C)-La(2)-N(6C)	60.91(10)	O(1)-La(1)-O(1B)	81.02(11)
N(4C)-La(2)-N(6B)	140.00(10)	O(1)-La(1)-N(9)	122.00(10)
N(4C)-La(2)-N(6)	72.31(10)	O(1)-La(1)-N(9B)	145.60(10)
N(4C)-La(2)-N(7C)	121.65(11)	O(1)-La(1)-N(9C)	78.41(11)
N(4C)-La(2)-N(7)	76.16(10)	O(1)-La(1)-N(11)	61.63(10)
N(4B)-La(2)-N(4)	87.84(11)	O(1)-La(1)-N(11B)	133.71(12)
N(4B)-La(2)-N(6C)	73.00(11)	O(1)-La(1)-N(11C)	73.57(10)
N(4B)-La(2)-N(6B)	60.53(11)	N(9)-La(1)-N(9B)	90.66(11)
N(4B)-La(2)-N(6)	141.44(11)	N(9)-La(1)-N(11B)	75.64(11)
N(4B)-La(2)-N(7C)	77.03(10)	N(9)-La(1)-N(11C)	145.14(11)
N(4B)-La(2)-N(7)	144.43(11)	N(9B)-La(1)-N(11B)	59.75(12)
N(6C)-La(2)-N(6B)	120.21(11)	N(9B)-La(1)-N(11C)	73.00(11)
N(6C)-La(2)-N(6)	118.64(11)	N(9C)-La(1)-N(9)	91.01(11)
N(6C)-La(2)-N(7)	71.46(11)	N(9C)-La(1)-N(9B)	91.65(11)
N(6B)-La(2)-N(6)	121.14(11)	N(9C)-La(1)-N(11)	74.90(11)
N(7C)-La(2)-N(4)	145.18(11)	N(9C)-La(1)-N(11B)	147.60(12)
N(7C)-La(2)-N(6C)	60.75(11)	N(9C)-La(1)-N(11C)	59.82(11)
N(7C)-La(2)-N(6B)	73.33(11)	N(11)-La(1)-N(9)	60.55(11)
N(7C)-La(2)-N(6)	141.50(11)	N(11)-La(1)-N(9B)	147.27(10)
N(7C)-La(2)-N(7)	87.00(11)	N(11)-La(1)-N(11B)	120.25(11)
N(7B)-La(2)-N(4)	75.39(11)	N(11)-La(1)-N(11C)	121.20(11)
N(7B)-La(2)-N(4C)	146.99(10)	N(11C)-La(1)-N(11B)	118.06(12)
N(7B)-La(2)-N(4B)	121.09(11)	N(1B)-Zn(1)-N(1)	99.52(14)
N(7B)-La(2)-N(6C)	141.04(11)	N(1B)-Zn(1)-N(1C)	93.52(14)
N(7B)-La(2)-N(6B)	60.57(10)	N(1C)-Zn(1)-N(1)	96.66(15)
N(7B)-La(2)-N(6)	74.71(10)	N(2B)-Zn(1)-N(1B)	75.44(14)
N(7B)-La(2)-N(7C)	85.81(11)	N(2B)-Zn(1)-N(1)	88.54(15)

N(7B)-La(2)-N(7)	88.56(11)	N(2B)-Zn(1)-N(1C)	168.48(14)
N(7)-La(2)-N(6B)	143.83(10)	N(2B)-Zn(1)-N(2C)	99.98(14)
N(7)-La(2)-N(6)	60.13(11)	N(2B)-Zn(1)-N(2)	102.89(14)
O(1C)-La(1)-O(1B)	83.02(11)	N(2C)-Zn(1)-N(1B)	87.01(14)
O(1C)-La(1)-N(9)	146.11(11)	N(2C)-Zn(1)-N(1)	170.39(15)
O(1C)-La(1)-N(9B)	78.39(11)	N(2C)-Zn(1)-N(1C)	75.79(14)
O(1C)-La(1)-N(9C)	120.95(11)	N(2)-Zn(1)-N(1B)	175.13(14)
O(1C)-La(1)-N(11)	134.15(10)	N(2)-Zn(1)-N(1)	75.78(14)
O(1C)-La(1)-N(11B)	71.12(11)	N(2)-Zn(1)-N(1C)	88.38(14)
O(1C)-La(1)-N(11C)	61.63(11)	N(2)-Zn(1)-N(2C)	97.80(14)
O(1B)-La(1)-N(9)	75.38(11)		

Table S17 Selected intra-ligand least-squares planes data of complex [(**L4**$_3$Zn)La$_2$](CF_3SO_3)$_8$·7.25(CH_3CN).

	Abbreviation	RMSD (Å)	Max deviation (Å) (Atom)
Ligand A			
N1 C1 C2 C3 C4 C5	Py1A	0.013	0.019 (C5)
N2 C6 N14 C7 C8 C9 C10 C11 C12	BZ1A	0.035	0.057 (C6)
C15 C14 C20 C19 C18 N5 C17 N4 C16	BZ2A	0.012	0.022 (C17)
N6 C21 C22 C23 C24 C25	Py2A	0.010	0.016 (C24)
C26 N8 C32 C31 C30 C29 C28 C27 N7	BZ3A	0.011	0.019 (C26)
C34 C40 C39 C38 N10 C37 N9 C36 C35	BZ4A	0.011	0.018 (C38)
N11 C41 C42 C43 C44 C45	Py3A	0.022	0.029 (C44,C41)
Ligand B			
N1B C1B C2B C3B C4B C5B	Py1B	0.010	0.013 (C2B)
N2B C6B N3B C7B C8B C9B C10B C11B C12B	BZ1B	0.034	0.056 (C6B)
C14B C20B C19B C18B N5B C17B N4B C16B C15B	BZ2B	0.010	0.020 (C17B)
C21B C22B C23B C24B C25B N6B	Py2B	0.003	0.005 (C23B)
C26B N8B C32B C31B C30B C29B C28B C27B N7B	BZ3B	0.021	0.035 (C26B)

C35B C34B C40B C39B C38B N10B C37B N9B C36B	BZ4B	0.017	0.027 (C36B)
C45B N11B C41B C42B C43B C44B	Py3B	0.014	0.024 (N11B)
Ligand C			
N1C C1C C2C C3C C4C C5C	Py1C	0.018	0.025 (C2C), C5C)
N2C C6C N3C C7C C8C C9C C10C C11C C12C	BZ1C	0.032	0.051 (C10C)
C15C C14C C20C C19C C18C N5C C17C N4C C16C	BZ2C	0.012	0.023 (N4C)
N6C C21C C22C C23C C24C C25C	Py2C	0.007	0.009 (C22C)
N7C C26C N8C C32C C31C C30C C29C C28C C27C	BZ3C	0.011	0.020 (C28C9
N9C C37C N10C C38C C39C C40C C34C C35C C36C	BZ4C	0.024	0.033 (C63C, C34C)
N11C C41C C42C C43C C44C C45C	Py3C	0.012	0.018 (N11C)

Table S18 Intra-ligand interplanar angles α (°) for $[(\mathbf{L4}_3Zn)La_2](CF_3SO_3)_8\cdot 7.25(CH_3CN)$.

	BZ1A	BZ2A	Py2A	BZ3A	BZ4A	Py3A
Py1A	16.41(15)	85.27(14)	103.24(18)	116.00(15)	43.61(15)	129.09(17)
BZ1A	0	70.85(9)	112.15(14)	120.87(10)	27.70(13)	143.92(15)
BZ2A		0	151.87(15)	135.67(12)	53.82(10)	123.38(14)
Py2A			0	17.99(15)	115.57(14)	71.74(17)
BZ3A				0	115.02(10)	74.39(13)
BZ4A					0	169.45(15)
Py3A						0

	BZ1B	BZ2B	Py2B	BZ3B	BZ4B	Py3B
Py1B	12.55(15)	85.95(15)	79.88(17)	64.76(15)	42.87(16)	160.4(2)
BZ1B	0	73.42(9)	68.74(13)	57.14(10)	33.84(14)	167.37(18)
BZ2B		0	23.54(13)	49.11(10)	56.53(10)	108.03(17)
Py2B			0	26.75(13)	66.02(13)	107.35(19)
BZ3B				0	70.99(9)	113.83(17)

BZ4B					0	156.70(18)
Py3B						0

	BZ1C	BZ2C	Py2C	BZ3C	BZ4C	Py3C
Py1C	12.81(16)	81.81(14)	77.77(17)	119.97(15)	135.57(16)	29.62(17)
BZ1C	0	70.50(9)	69.96(13)	123.32(10)	146.84(12)	21.50(14)
BZ2C		0	23.70(14)	133.64(10)	121.85(10)	72.23(13)
Py2C			0	154.31(14)	108.60(13)	80.36(16)
BZ3C				0	72.86(9)	105.88(13)
BZ4C					0	162.38(13)
Py3C						0

Table S19 Selected least-squares chelate planes data of complex [(**L4**$_3$Zn)La$_2$](CF$_3$SO$_3$)$_8$·7.25(CH$_3$CN).

	Abbreviation	RMSD (Å)	Max deviation (Å) (Atom)
Terminal tridentate site			
La1 O1 C46 C45 N11	Ch1A	0.131	0.198 (C46)
La1 N11 C41 C37 N9	Ch2A	0.097	0.133 (N9)
La1 O1B C46B C45B N11B	Ch1B	0.134	0.197 (C46B)
La1 N11B C41B C37B N9B	Ch2B	0.114	0.166 (N11B)
La1 O1C C46C C45C N11C	Ch1C	0.135	0.216 (C46C)
La1 N11C C41C C37C N9C	Ch2C	0.082	0.118 (C41C-N11C)
Central tridentate site			
La2 N4 C17 C21 N6	Ch3A	0.129	0.192 (C17)
La2 N6 C25 C26 N7	Ch4A	0.154	0.213 (N7)
La2 N4B C17B C21B N6B	Ch3B	0.145	0.206 (N4B)
La2 N6B C25B C26B N7B	Ch4B	0.168	0.241 (N7B)
La2 N6C C21C C17C N4C	Ch3C	0.155	0.220 (N4C)
La2 N7C C26C C25C N6C	Ch4C	0.145	0.207 (N7C)
Didentate site			
Zn1 N2 C6 C5 N1	Ch5A	0.084	0.120 (N1)
Zn1 N1B C5B C6B N2B	Ch5B	0.093	0.122 (N1B)

Zn1 N1C C5C C6C N2C	Ch5C	0.073	0.103 (N1C)

Table S20 Interplanar chelate angles β (°) for [(**L4**$_3$Zn)La$_2$](CF$_3$SO$_3$)$_8$·7.25(CH$_3$CN).

	Ch2A	Ch1B	Ch2B	Ch1C	Ch2C
Ch1A	6.33(9)	72.1(1)	76.6(2)	72.8(1)	67.82(9)
Ch2A	0	67.4(1)	72.0(1)	75.34(15)	70.56(12)
Ch1B		0	4.57(15)	66.71(14)	67.67(16)
Ch2B			0	67.2(1)	68.61(13)
Ch1C				0	5.41(11)
Ch2C					0

	Ch4A	Ch3B	Ch4B	Ch3C	Ch4C
Ch3A	8.8(1)	66.0(1)	60.09(9)	64.3(1)	68.7(1)
Ch4A	0	71.6(2)	66.7(1)	59.07(9)	64.6(1)
Ch3B		0	10.08(9)	68.3(1)	61.37(9)
Ch4B			0	73.9(1)	68.1(1)
Ch3C				0	9.51(9)
Ch4C					0

	Ch5A	Ch5B	Ch5C
Ch5A	0	86.0(1)	86.5(1)
Ch5B		0	83.8(1)
Ch5C			0

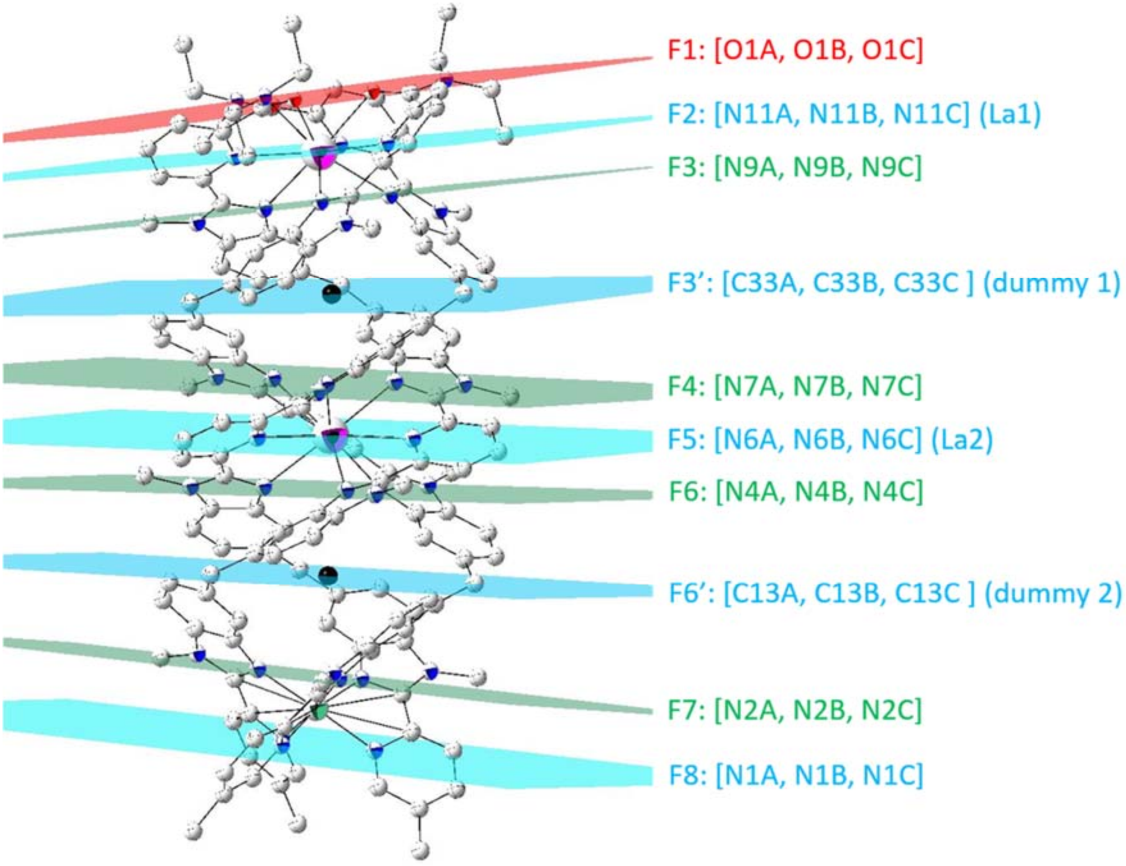

Fig. S25 Helical portions F*i*-F*j* in the molecular structure of *HHH*-[(**L4**$_3$Zn)La$_2$]$^{8+}$

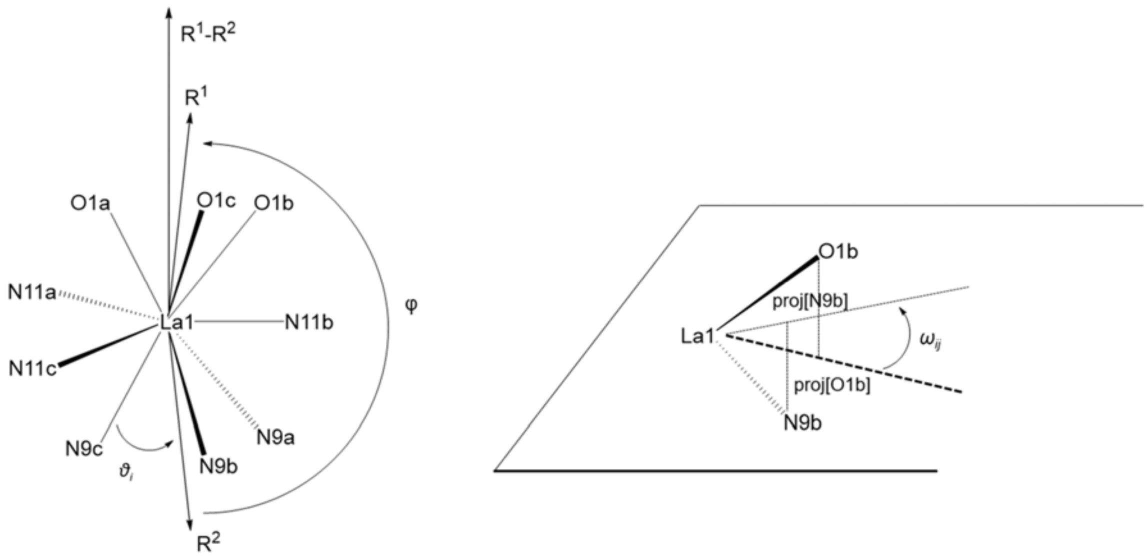

Fig. S26 Definitions of angles φ, θ_i, and ω_{ij} for La1 coordination sphere in *HHH*-[(**L4**$_3$Zn)La$_2$]$^{8+}$.

Table S21 Selected intra-ligand least-squares planes data of complex $[(\mathbf{L4}_3Zn)Lu_2](CF_3SO_3)_8$.[57]

	Abbreviation	RMSD (Å)	Max deviation (Å) (Atom)
Ligand A			
N1A C1A C2A C3A C4A C5A	Py1A	0.015	0.024 (N1A)
C6A N3A C12A C11A C10A C9A C8A C7A N2A	BZ1A	0.052	0.100 (C6A)
N4A C16A C15A C14A C19A C18A C17A N5A C20A	BZ2A	0.035	0.055 (N5A)
N6A C21A C22A C23A C24A C25A	Py2A	0.008	0.012 (C21A)
C26A N8A C32A C31A C30A C29A C28A C27A N7A	BZ3A	0.009	0.017 (N8A)
N9A C40A N10A C37A C38A C39A C34A C35A C36A	BZ4A	0.033	0.048 (C36A)
N11A C41A C42A C43A C44A C45A	Py3A	0.041	0.056 (C41A)
Ligand B			
N1B C1B C2B C3B C4B C5B	Py1B	0.028	0.042 (C4B)
C6B N2B N3B C12B C11B C10B C9B C8B C7B	BZ1B	0.043	0.072 (C6B)
C15B C14B C19B C18B C17B N5B C20B N4B C16B	BZ2B	0.026	0.041 (C17B)
N6B C21B C22B C23B C24B C25B	Py2B	0.018	0.030 (C24B)
N7B C26B N8B C32B C31B C30B C29B C28B C27B	BZ3B	0.049	0.075 (C30B)
C34B C39B C38B C37B N10B C40B N9B C36B C35B	BZ4B	0.053	0.103 (C34B)
N11B C41B C42B C43B C44B C45B	Py3B	0.031	0.052 (C41B)
Ligand C			
N1C C1C C2C C3C C4C C5C	Py1C	0.024	0.038 (C2C)
N2C C6C N3C C12C C11C C10C C9C C8C C7C	BZ1C	0.038	0.052 (C7C)
C14C C19C C18C C17C N5C C20C N4C C16C C15C	BZ2C	0.037	0.051 (N4C)
N6C C21C C22C C23C C24C C25C	Py2C	0.018	0.026 (C22C)

N7C C26C N8C C32C C31C C30C C29C C28C C27C	BZ3C	0.023	0.046 (N7C)
N9C C40C N10C C37C C38C C39C C34C C35C C36C	BZ4C	0.023	0.038 (C35C)
N11C C41C C42C C43C C44C C45C	Py3C	0.046	0.076 (N11C)

Table S22 Intra-ligand interplanar angles α (°) for [(**L4**$_3$Zn)Lu$_2$](CF$_3$SO$_3$)$_8$.[57]

	BZ1A	BZ2A	Py2A	BZ3A	BZ4A	Py3A
Py1A	5.0(7)	93.5(6)	105.8(8)	118.6(6)	42.9(7)	25.1(8)
BZ1A	0	97.7(5)	108.9(7)	119.6(5)	38.2(6)	21.0(7)
BZ2A		0	21.1(7)	49.4(5)	116.7(5)	104.1(7)
Py2A			0	28.3(7)	114.5(7)	108.0(9)
BZ3A				0	105.4(4)	108.4(7)
BZ4A					0	18.6(7)
Py3A						0

	BZ1B	BZ2B	Py2B	BZ3B	BZ4B	Py3B
Py1B	166.8(6)	91.0(6)	81.9(7)	67.5(6)	132.8(7)	130.5(8)
BZ1B	0	77.6(4)	105.8(6)	115.0(5)	34.1(6)	36.4(7)
BZ2B		0	156.0(6)	130.6(5)	54.4(5)	51.2(7)
Py2B			0	26.2(6)	115.9(6)	118.7(8)
BZ3B				0	108.8(5)	110.4(7)
BZ4B					0	3.2(7)
Py3B						0

	BZ1C	BZ2C	Py2C	BZ3C	BZ4C	Py3C
Py1C	13.5(5)	88.2(5)	97.8(7)	63.1(6)	130.2(6)	111.3(7)
BZ1C	0	100.6(3)	107.0(6)	58.2(4)	142.6(5)	123.5(7)
BZ2C		0	24.5(6)	130.4(5)	58.5(5)	52.2(7)

Py2C	0	153.4(7)	67.7(6)	69.0(8)
BZ3C		0	109.3(4)	99.5(7)
BZ4C			0	19.1(8)
Py3C				0

Table S23. Selected least-squares chelate planes data of complex $[(\mathbf{L4}_3Zn)Lu_2](CF_3SO_3)_8$.[57]

	Abbreviation	RMSD (Å)	Max deviation (Å) (Atom)
Terminal tridentate site			
Lu2 O1A C46A C45A N11A	Ch1A	0.133	0.202 (C46A)
Lu2 N11A C41A C40A N9A	Ch2A	0.137	0.191 (N11A)
Lu2 O1B C46B C45B N11B	Ch1B	0.135	0.203 (O1B)
Lu2 N11B C41B C40B N9B	Ch2B	0.116	0.161 (N9B)
Lu2 O1C C46C C45C N11C	Ch1C	0.078	0.119 (C46C)
Lu2 N11C C41C C40C N9C	Ch2C	0.066	0.101 (C40C)
Central tridentate site			
Lu1 N4A C20A C21A N6A	Ch3A	0.146	0.208 (N4A)
Lu1 N6A C25A C26A N7A	Ch4A	0.161	0.226 (N7A)
Lu1 N4B C20B C21B N6B	Ch3B	0.155	0.211 (N4B)
Lu1 N6B C25B C26B N7B	Ch4B	0.135	0.193 (N7B)
Lu1 N4C C20C C21C N6C	Ch3C	0.133	0.186 (Lu1)
Lu1 N6C C25C C26C N7C	Ch4C	0.154	0.216 (N7C)
Didentate site			
Zn N2A C6A C5A N1A	Ch5A	0.060	0.086 (C5A)
Zn N1B C5B C6B N2B	Ch5B	0.055	0.078 (N1B,C5B)
Zn N1C C5C C6C N2C	Ch5C	0.066	0.089 (N1C)

Table S24. Interplanar chelate angles β (°) for [(**L4**$_3$Zn)Lu$_2$](CF$_3$SO$_3$)$_8$.[57]

	Ch2A	Ch1B	Ch2B	Ch1C	Ch2C
Ch1A	5.9(5)	106.0(6)	113.3(4)	103.8(5)	108.8(7)
Ch2A	0	101.1(7)	108.1(6)	105.9(5)	110.3(6)
Ch1B		0	8.6(5)	73.7(6)	67.4(5)
Ch2B			0	77.0(7)	70.2(7)
Ch1C				0	7.4(8)
Ch2C					0

	Ch4A	Ch3B	Ch4B	Ch3C	Ch4C
Ch3A	168.7(5)	113.3(6)	109.7(6)	66.5(5)	59.9(4)
Ch4A	0	61.3(4)	66.9(5)	105.3(6)	110.7(5)
Ch3B		0	11.3(5)	110.6(6)	104.7(6)
Ch4B			0	119.9(4)	112.8(5)
Ch3C				0	10.6(4)
Ch4C					0

	Ch5A	Ch5B	Ch5C
Ch5A	0	90.5(6)	93.0(5)
Ch5B		0	86.2(5)
Ch5C			0

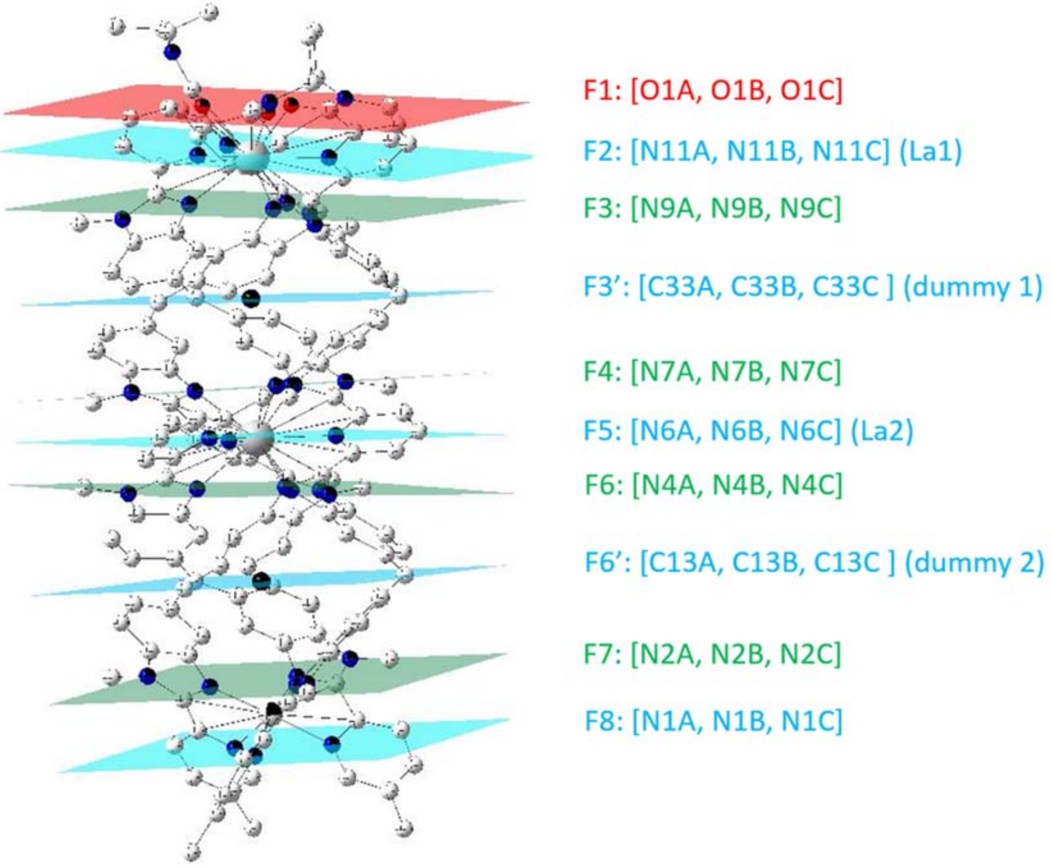

Fig. S27 Helical portions F*i*-F*j* in the molecular structure of [*HHH*-[($\mathbf{L4}_3$Zn)Lu_2]$^{8+}$.[57]

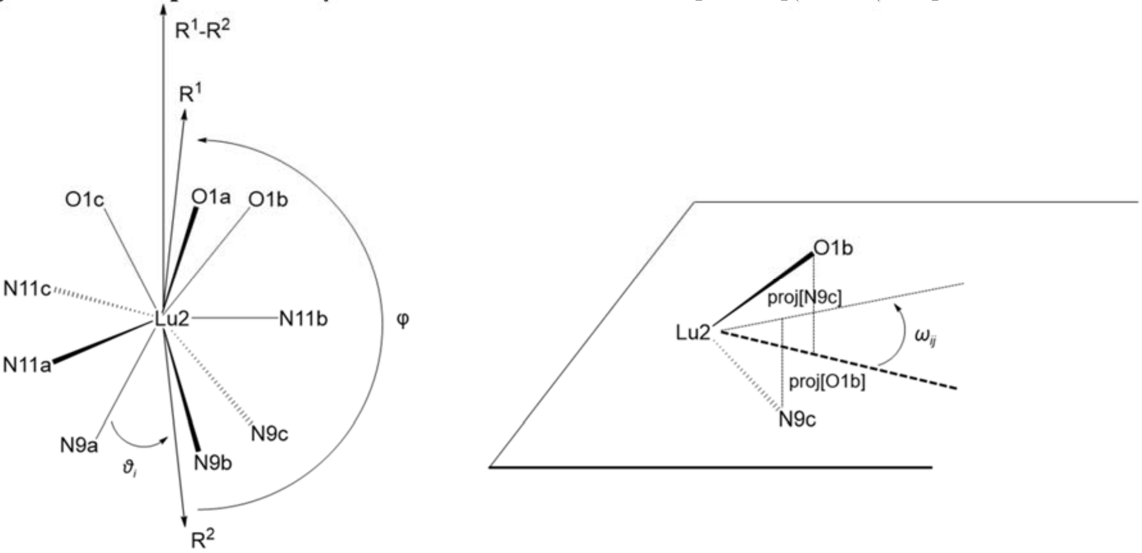

Fig. S28 Definitions of angles φ, θ_i, and ω_{ij} for Lu2 coordination sphere in [*HHH*-[($\mathbf{L4}_3$Zn)Lu_2]$^{8+}$.[57]

Table S25. Helical pitches P_{ij}, linear distances d_{ij} and average twist angle ω_{ij} along the pseudo-C_3 axis in the molecular structures of *HHH*-[(**L4**$_3$Zn)Ln$_2$]$^{8+}$ as found in the associated crystal structures of [(**L4**$_3$Zn)Ln$_2$](CF$_3$SO$_3$)$_8$ (Ln = La, Eu, Lu).

Helical portion [a]	*HHH*-[(**L4**$_3$Zn)La$_2$]$^{8+}$			*HHH*-[(**L4**$_3$Zn)Eu$_2$]$^{8+}$			*HHH*-[(**L4**$_3$Zn)Lu$_2$]$^{8+}$		
	d_{ij} / Å	ω_{ij} /°	P_{ij} / Å	d_{ij} / Å	ω_{ij} / °	P_{ij} / Å	d_{ij} / Å	ω_{ij} /°	P_{ij} / Å
F1-F2 [b]	1.5112	53	10.3	1.4507	55.52	9.41	1.57	61.1	9.2
F2-F3	1.6361	50.6	11.6	1.7039	53.1	11.55	1.53	56.8	9.7
F3-F3'	2.8201	58.8	17.3	2.9591	60.2	17.7	2.98	60.1	17.8
F3'-F4	2.6663	61.3	15.7	2.784	63.06	15.89	2.82	63.3	16.0
F4-F5	1.6296	51.6	11.4	1.6659	53	11.32	1.62	56.2	10.4
F5-F6	1.6093	52.2	11.1	1.5913	53.41	10.73	1.60	55.8	10.3
F6-F6'	2.6554	61.2	15.6	2.9209	62.93	16.71	2.95	61.1	17.4
F6'-F7	3.058	52.8	20.85	3.1434	55.98	20.21	3.23	56.8	20.5
F7-F8	2.1089	50.8	14.95	2.1286	52.05	14.72	2.14	49.5	15.6
Average pitch /Å	14.4			14.1			14.1		
Total length /Å	19.7			20.3			20.4		

[a] Each helical portion F*i*-F*j* is characterized by (i) a linear extension d_{ij} defined by the separation between the facial planes, (ii) an average twist angle ω_{ij} defined by the angular rotation between the projections of N*i* and N*j* belonging to the same ligand strand (Fig.s S23, S26 and S28) and (iii) its pitch P_{ij} defined as the ratio of axial over angular progressions along the helical axis. $P_{ij} = (d_{ij}/\omega_{ij})\cdot 360$ corresponds to the length of a cylinder containing a single turn of the helix defined by the geometrical characteristics d_{ij} and ω_{ij}. [b] See Fig.s S22, S25 and S27for the definitions of the planes F*i*-F*j*.

Table S26 Structural parameters observed in the molecular structures of *HHH*-[(**L4**$_3$Zn)Ln$_2$]$^{8+}$ as found in the associated crystal structures of [(**L4**$_3$Zn)Ln$_2$](CF$_3$SO$_3$)$_8$ (Ln = La, Eu, Lu).

	[(**L4**$_3$Zn)La$_2$]$^{8+}$	[(**L4**$_3$Zn)Eu$_2$]$^{8+}$	[(**L4**$_3$Zn)Lu$_2$]$^{8+\,a}$
d (Zn-Ln$_c$) / Å ^{b}	8.322(1)	8.652(1)	8.781(2)
d (Ln$_c$-Ln$_t$) / Å ^{b}	8.627(1)	8.917(1)	8.939(1)
d (Zn-Ln$_t$) / Å	16.930(1)	17.555(1)	17.718(2)
Zn-Ln$_c$-Ln$_t$ / °	174.72(1)	175.41(1)	178.12(2)
$V_{molecular}$ / Å$^{3\,c}$	2811	2809	2786
Total length / Å	19.7	20.3	20.4
Total helical twist / °	492.3	509.3	520.7
Helical pitch / Å	14.4	14.1	14.1
d (Zn-N$_{py}$) / Å	2.208(13)	2.194(19)	2.20(3)
d (Zn-N$_{bz}$) / Å	2.142(11)	2.105(16)	2.10(2)
Octahedron (Zn) ^{c}	1.697	1.486	1.549
Trigonal prism (Zn) ^{c}	14.579	14.306	14.500
d (Ln$_c$-N$_{bz}$) / Å	2.670(25)	2.584(27)	2.50(2)
d (Ln$_c$-N$_{py}$) / Å	2.716(16)	2.609(7)	2.51(1)
Spherical capped squared antiprism (Ln$_c$) ^{c}	2.821	1.983	1.311
Tricapped trigonal prism (Ln$_c$) ^{c}	2.433	2.158	1.867
d (Ln$_t$-N$_{bz}$) / Å	2.695(18)	2.578(16)	2.48(3)
d (Ln$_t$-N$_{py}$) / Å	2.721(34)	2.595(25)	2.51(1)
d (Ln$_t$-O) / Å	2.451(24)	2.389(39)	2.29(2)
Spherical capped squared antiprism (Ln$_t$) ^{c}	3.060	2.039	1.751
Tricapped trigonal prism (Ln$_t$) ^{c}	2.358	2.025	1.568

^{a} Calculated from reference 57. ^{b} Ln$_c$ = lanthanide bound in the central [Ln$_c$N$_9$] unit, Ln$_t$ = lanthanide in the terminal [Ln$_t$N$_6$O$_3$] unit. ^{c} Molecular Connolly volume computed by using a solvent molecule with a 1.4 Å radius.[64] ^{c}SHAPE's scores for the closest idealized coordination geometries.[69-71].

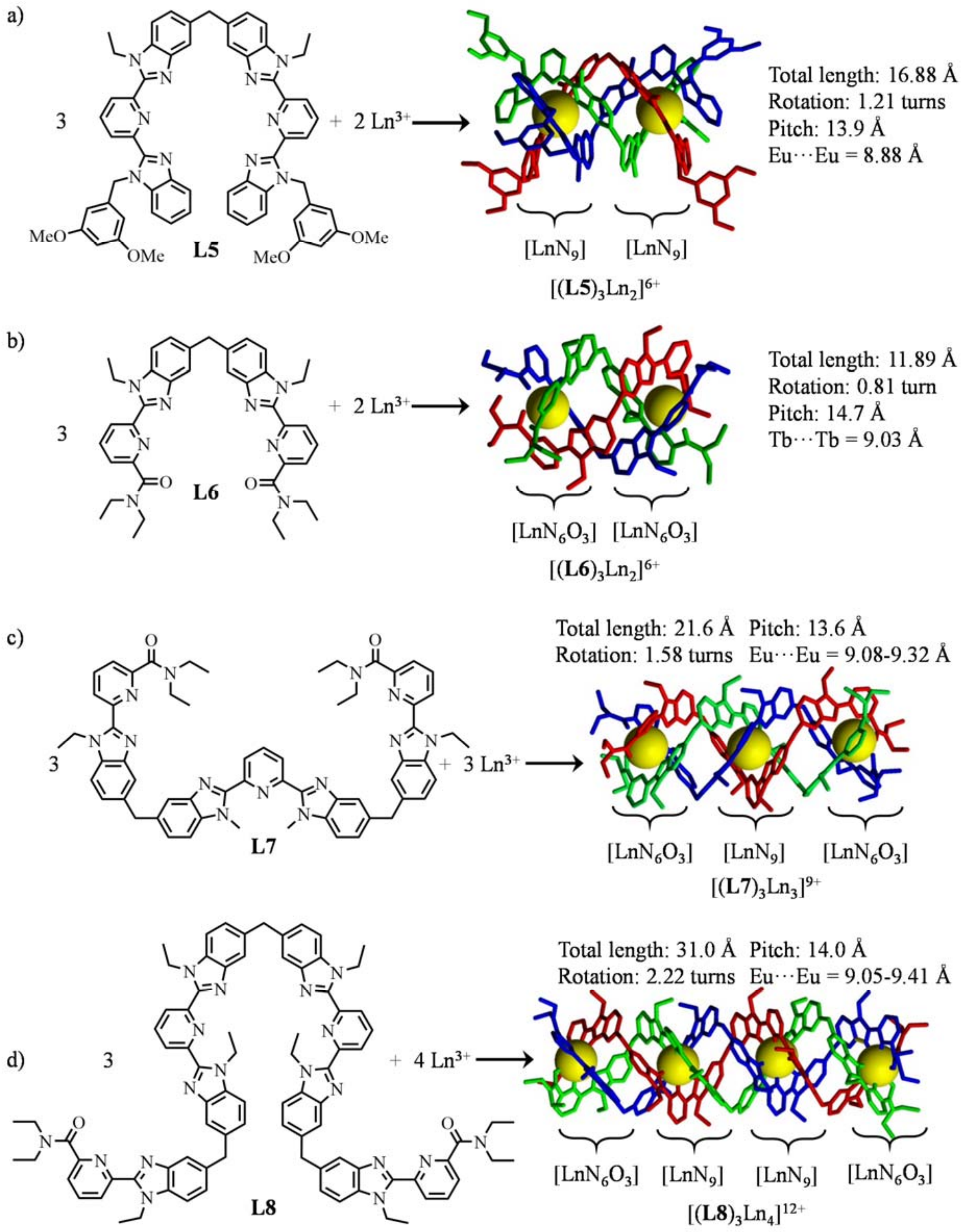

Fig. S29 Formation and molecular structures of dinuclear a) $[(\mathbf{L5})_3\mathrm{Ln}_2]^{6+}$ [65] and b) $[(\mathbf{L6})_3\mathrm{Ln}_2]^{6+}$,[66] c) trinuclear $[(\mathbf{L7})_3\mathrm{Ln}_3]^{9+}$ [67] and d) tetranuclear $[(\mathbf{L8})_3\mathrm{Ln}_4]^{12+}$ [68] homolanthanide triple-stranded helicates containing [$\mathrm{LnN_9}$] and [$\mathrm{LnN_6O_3}$] coordination sites similar to those found in *HHH*-$[(\mathbf{L4}_3\mathrm{Zn})\mathrm{Ln}_2]^{8+}$.

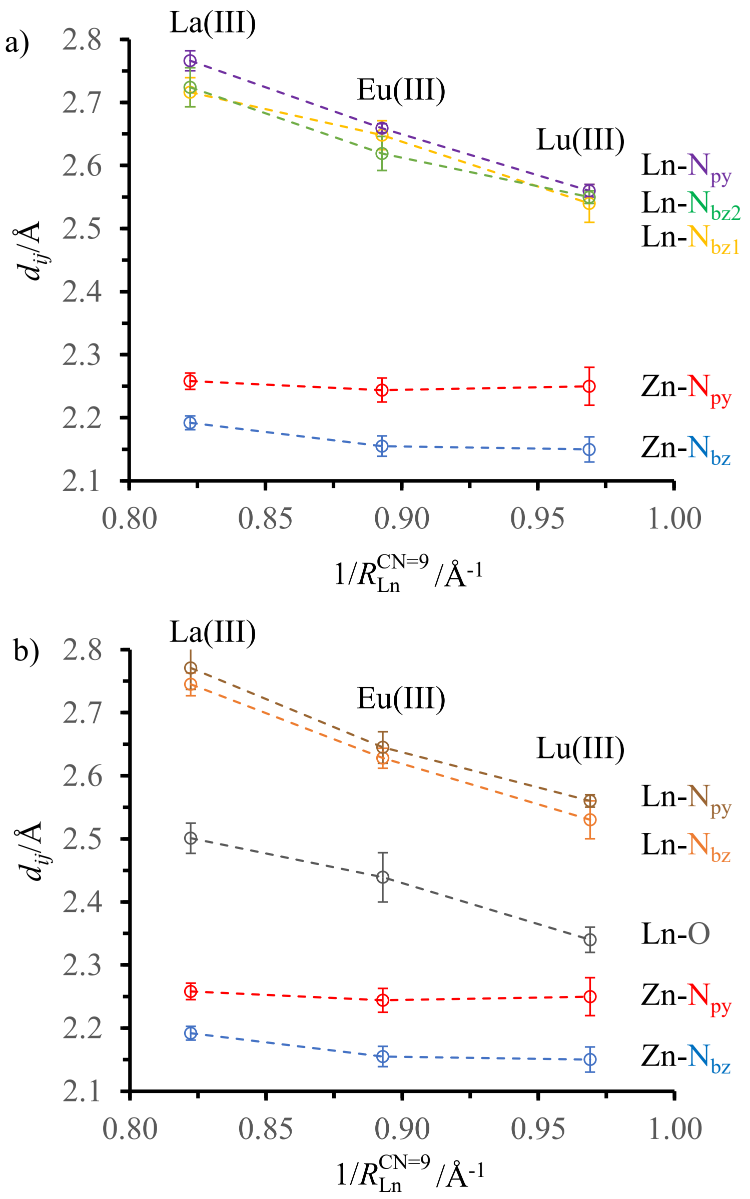

Fig. S30 Average bond lengths measured for the coordination spheres of Zn(II) and a) Ln(III) from the central N_9 tridentate site and b) Ln(III) from the terminal N_6O_3 tridentate site as a function of the inverse of the nine-coordinate lanthanide ionic radii[51] in [*HHH*-[(**L4**$_3$Zn)Ln_2]$^{8+}$ (Ln = La(III), Eu(III), Lu(III)). Standard deviations of the averages are shown with vertical error bars. The dashed traces are only guides for the eyes.

Table S27. Average $v_{\text{M-donor}}$ bond valences (eqn 4) and bond valence sum parameter $V_i = \sum_j v_{ij} = \sum_j e^{[(R_{ij}-d_{ij})/b]}$ calculated with eqn (1) in the molecular structures of *HHH*-[(**L4**$_3$Zn)Ln$_2$]$^{8+}$ as found in the associated crystal structures of [(**L4**$_3$Zn)Ln$_2$](CF$_3$SO$_3$)$_8$ (Ln = La, Eu, Lu)..

		[(**L4**$_3$Zn)La$_2$]$^{8+}$	[(**L4**$_3$Zn)Eu$_2$]$^{8+}$	[(**L4**$_3$Zn)Lu$_2$]$^{8+}$
Zn(II)	$v_{\text{Zn(II)-N(py)}}$ [c]	0.31(1)	0.32(2)	0.31(2)
	$v_{\text{Zn(II)-N(bz)}}$ [d]	0.37(1)	0.40(2)	0.40(2)
	V_{Zn}	2.01	2.17	2.17
Ln(III)$_c$ [a]	$v_{\text{Ln(III)-N(bz)}}$ [d]	0.33(2)	0.32(2)	0.30(2)
	$v_{\text{Ln(III)-N(py)}}$ [c]	0.29(1)	0.298(6)	0.285(8)
	$V_{\text{Ln,c}}$	2.87	2.81	2.62
Ln(III)$_t$ [b]	$v_{\text{Ln(III)-N(bz)}}$ [d]	0.31(1)	0.32(1)	0.31(2)
	$v_{\text{Ln(III)-N(py)}}$ [c]	0.29(3)	0.31(2)	0.288(9)
	$v_{\text{Ln(III)-O}}$	0.44(3)	0.39(4)	0.40(2)
	$V_{\text{Ln,t}}$	3.12	3.07	2.99

[a] Ln(III)$_c$ = trivalent lanthanide coordinated at the central bis(benzimidazole)pyridine N$_9$ tridentate site. [b] Ln(III)$_t$ = trivalent lanthanide coordinated at the terminal benzimidazole-pyridine- carbonyl N$_6$O$_3$ tridentate site. [c] py = pyridine. [d] bz = benzimidazole.

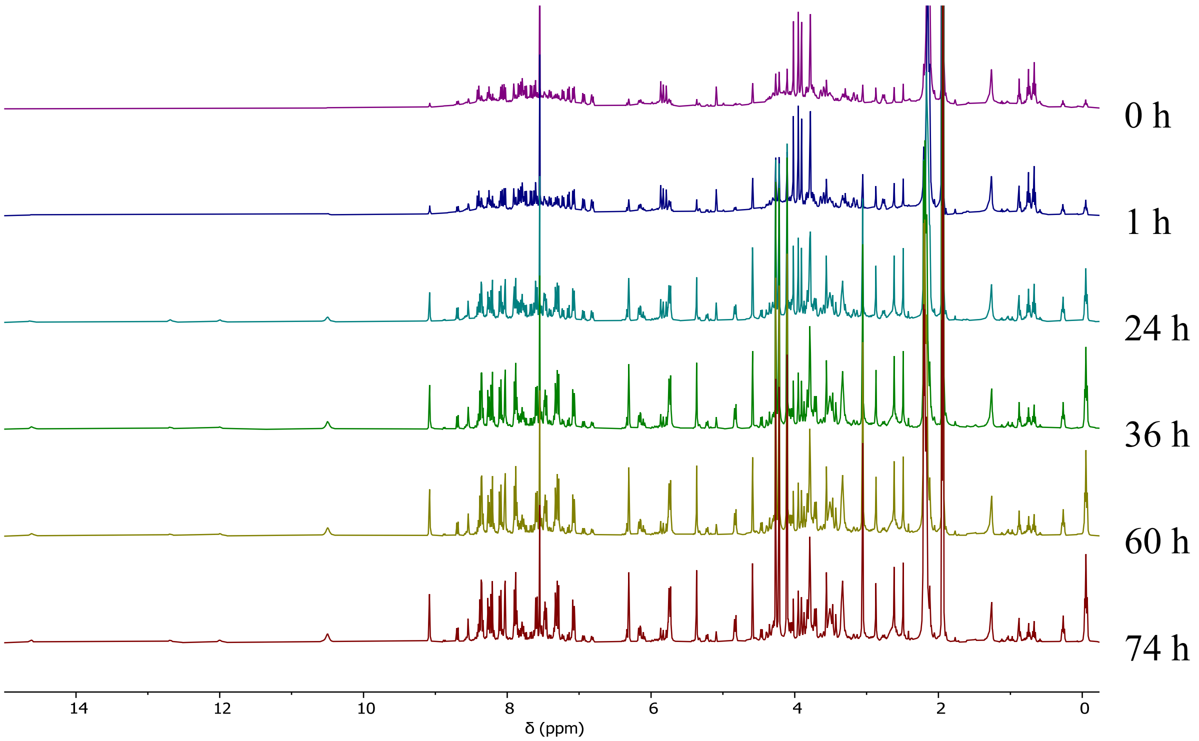

Fig. S31 Evolution of the ^{1}H-NMR spectrum (400 MHz, 2:1 $CD_3CN/CDCl_3$, 298 K) of a 1:1:1:3 mixture of $Zn(CF_3SO_3)_2$, $La(CF_3SO_3)_3$, $Eu(CF_3SO_3)_3$ and **L4** over 74 hours.

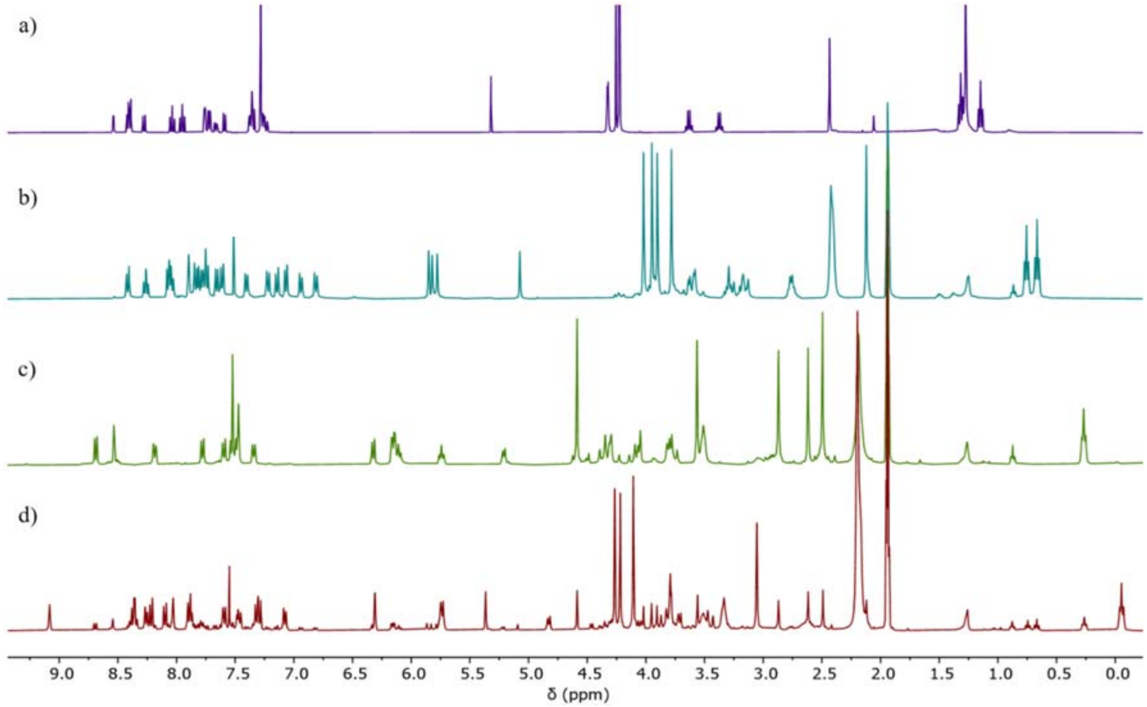

Fig. S32 Comparison of the ^{1}H-NMR spectrum (400 MHz, 298 K) of a) **L4** ($CDCl_3$), b) *HHH*-$[(\mathbf{L4}_3Zn)La_2]^{8+}$ (2:1 $CD_3CN/CDCl_3$), c) *HHH*-$[(\mathbf{L4}_3Zn)Eu_2]^{8+}$ (2:1 $CD_3CN/CDCl_3$) and d) a 1:1:1:3 mixture of $Zn(CF_3SO_3)_2$, $La(CF_3SO_3)_3$, $Eu(CF_3SO_3)_3$ and **L4** at equilibrium (2:1 $CD_3CN/CDCl_3$).

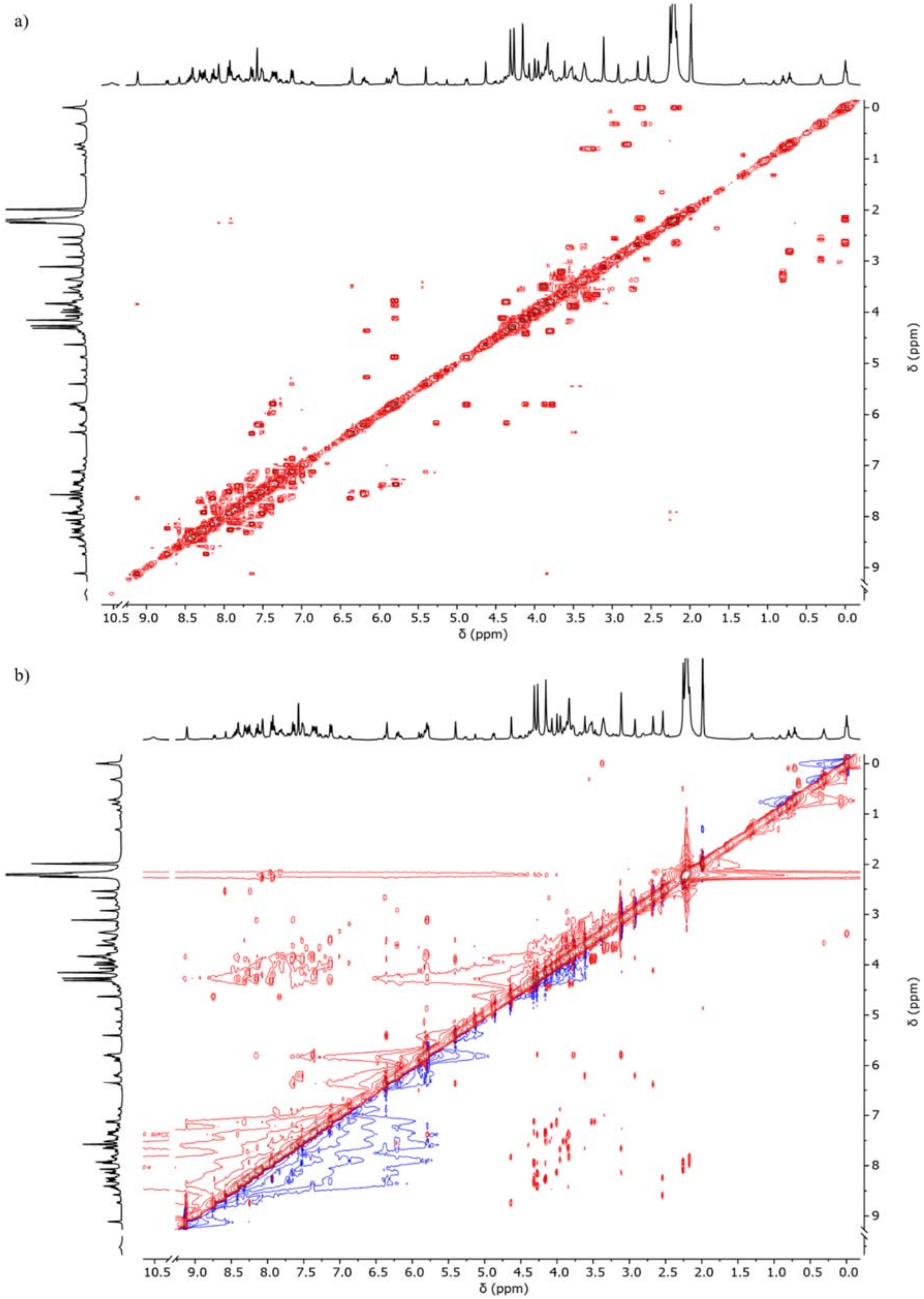

Fig. S33 a) COSY and b) NOESY of the 1:1:1:3 mixture of Zn^{II}:La^{III}:Eu^{III}:**L4** at equilibrium (2:1 CD_3CN/$CDCl_3$, 400 MHz, 298 K).

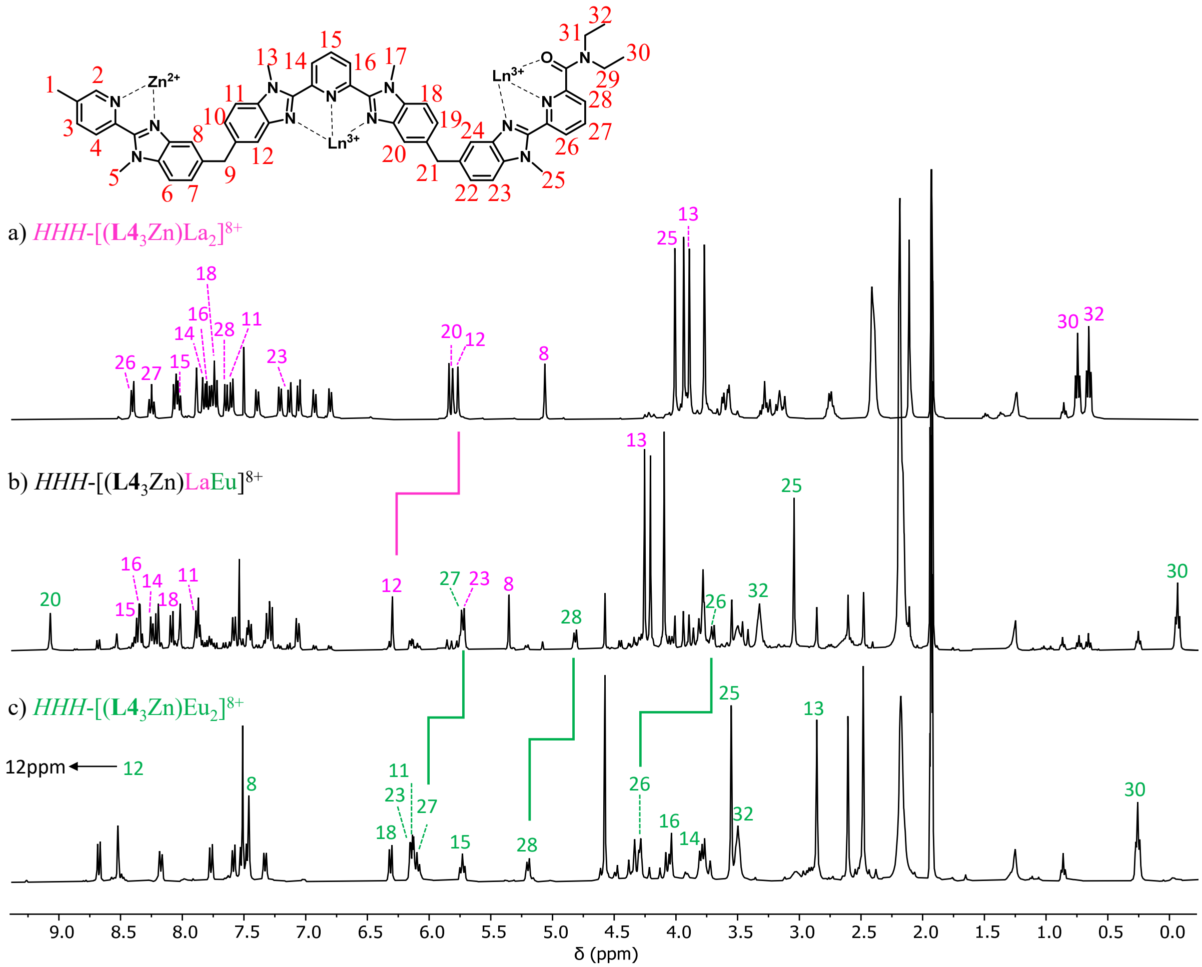

Fig. S34 Comparison of the ^{1}H-NMR spectrum of a) *HHH*-[(**L4**$_3$Zn)La$_2$]$^{8+}$, b) 1:1:1:3 mixture of Zn(CF_3SO_3)$_2$, La(CF_3SO_3)$_3$, Eu(CF_3SO_3)$_3$ and **L4** at equilibium and c) *HHH*-[(**L4**$_3$Zn)Eu$_2$]$^{8+}$ highlighting some protons (12, 26, 27, 28) relevant to the determination of the position of the lanthanide ions in each binding site (400 MHz, 2:1 CD_3CN/$CDCl_3$, 298 K).

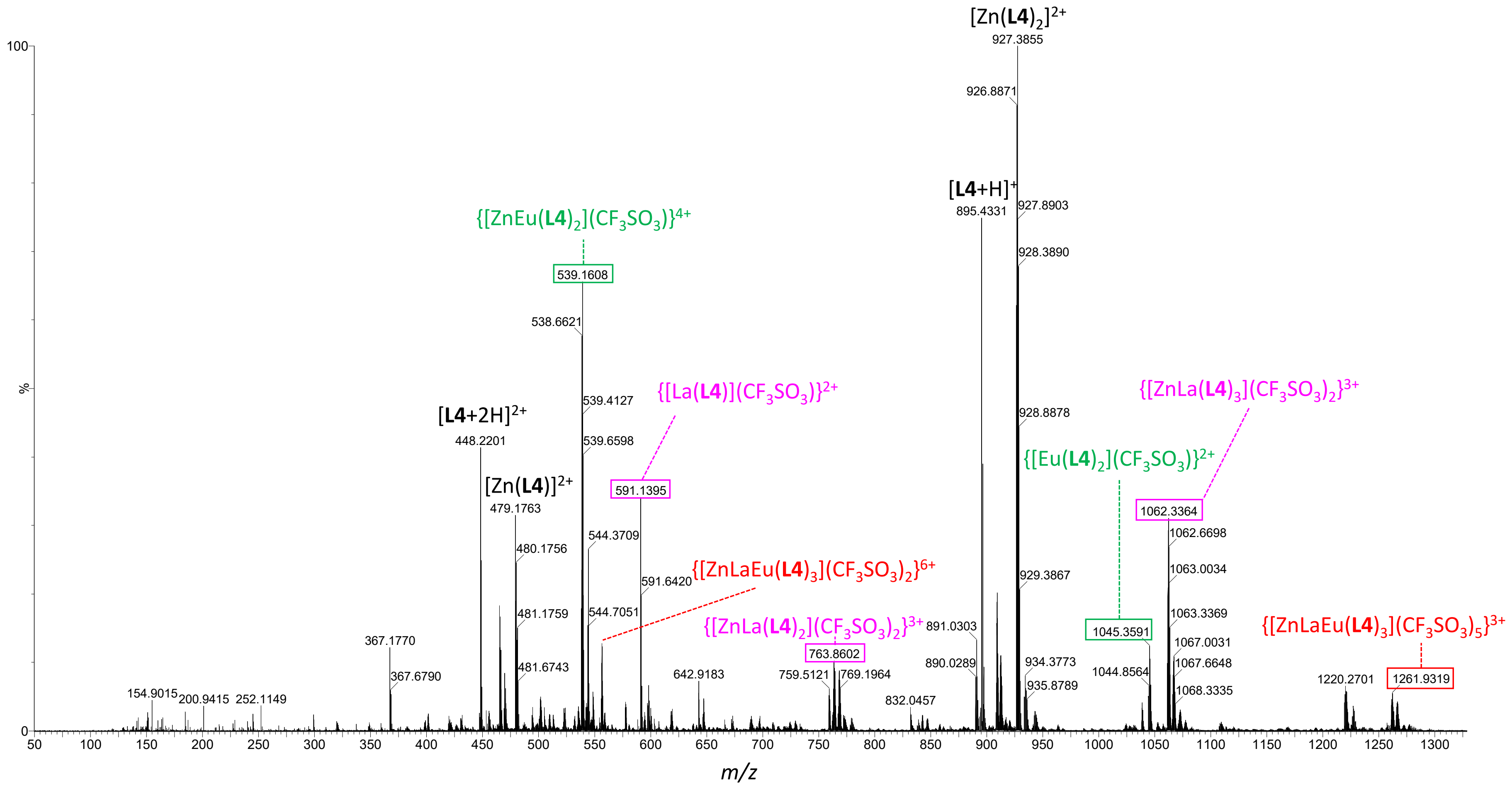

Fig. S35 High-resolution ESI-TOF spectrum of a 1:1:1:3 mixture of $Zn(CF_3SO_3)_2$ (0.49 mM), $La(CF_3SO_3)_3$ (0.51 mM), $Eu(CF_3SO_3)_3$ (0.50 mM) and **L4** (1.6 mM) in a 2:1 mixture of $CD_3CN/CDCl_3$.

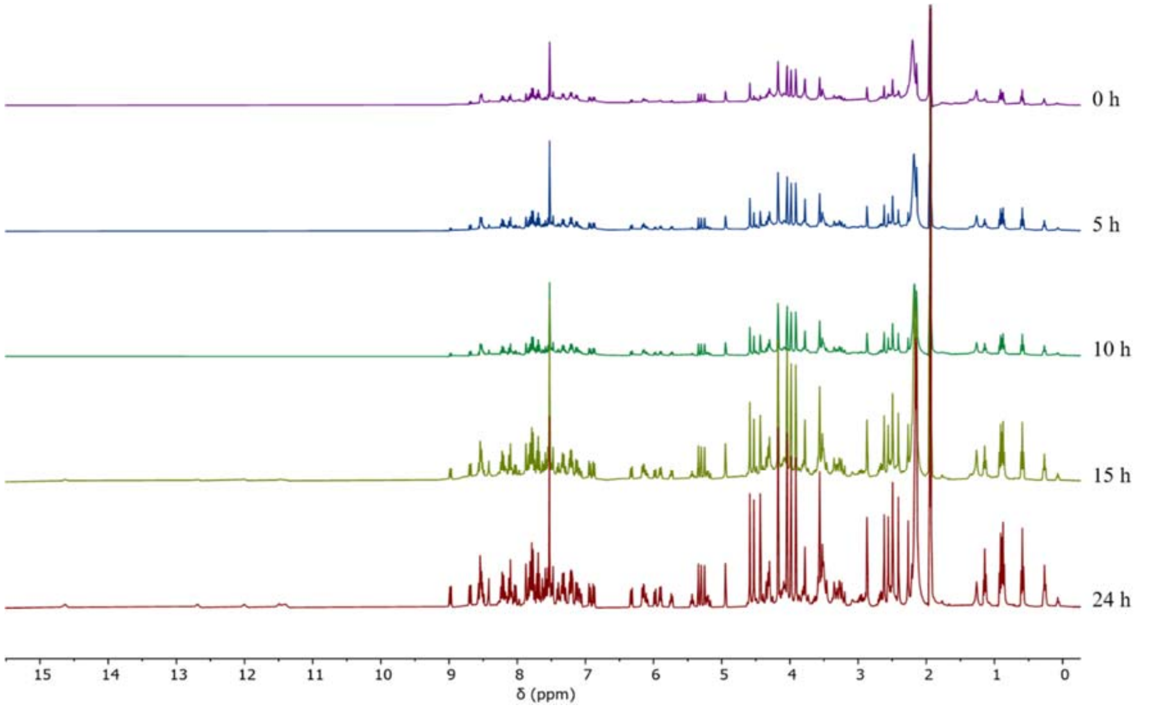

Fig. S36 Evolution of the ^{1}H-NMR spectrum (400 MHz, 2:1 $CD_3CN/CDCl_3$, 298 K) of a 1:1:1:3 mixture of $Zn(CF_3SO_3)_2$, $Eu(CF_3SO_3)_3$, $Lu(CF_3SO_3)_3$ and **L4** over 24 hours.

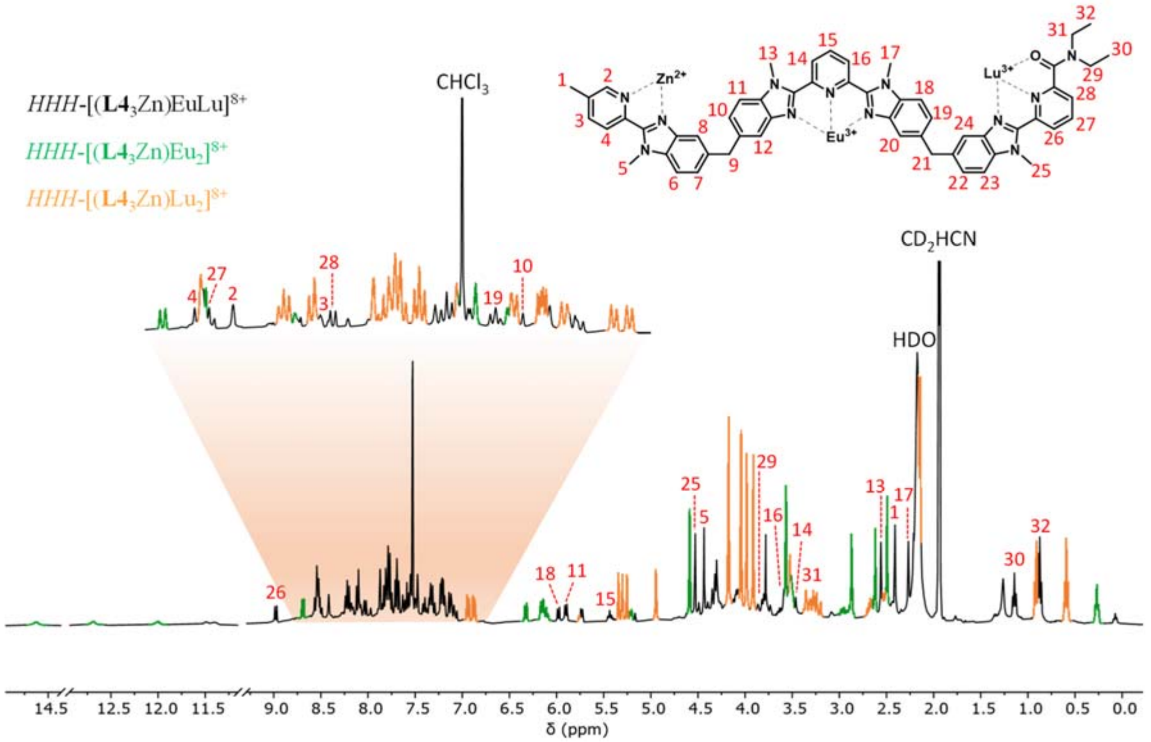

Fig. S37 ^{1}H-NMR spectrum (2:1 $CD_3CN/CDCl_3$, 400 MHz, 298 K) of 1:1:1:3 mixture of $Zn(CF_3SO_3)_2$, $Eu(CF_3SO_3)_3$, $Lu(CF_3SO_3)_3$ and **L4** at equilibrium. The aromatic region was expanded for clarity.

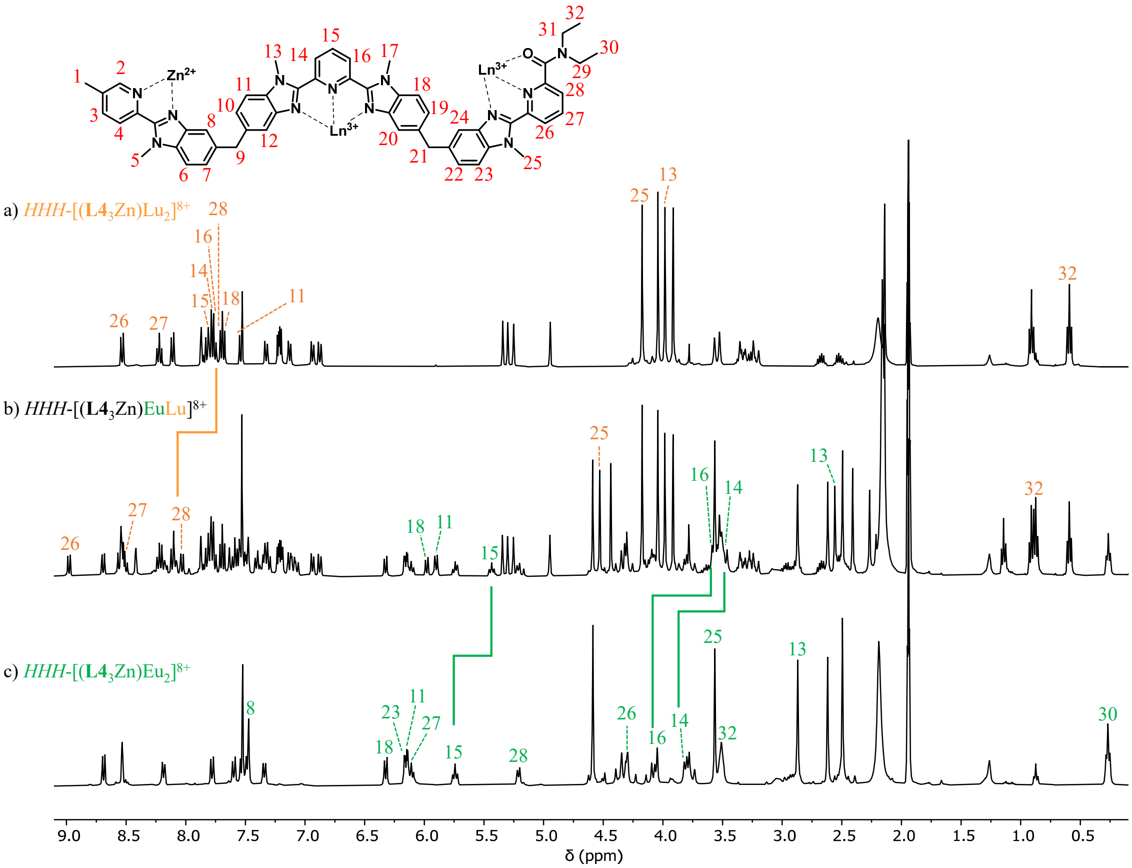

Fig. S38. Comparison of the ^{1}H-NMR spectrum of a) *HHH*-[(**L4**$_3$Zn)Lu$_2$]$^{8+}$, b) 1:1:1:3 mixture of Zn(CF$_3$SO$_3$)$_2$, Lu(CF$_3$SO$_3$)$_3$, Eu(CF$_3$SO$_3$)$_3$ and **L4** at equilibrium and c) *HHH*-[(**L4**$_3$Zn)Eu$_2$]$^{8+}$ highlighting some protons (14, 15, 16, 28) relevant to the determination of the position of the lanthanide ions in each site (400 MHz, 2:1 CD$_3$CN/CDCl$_3$, 298 K).

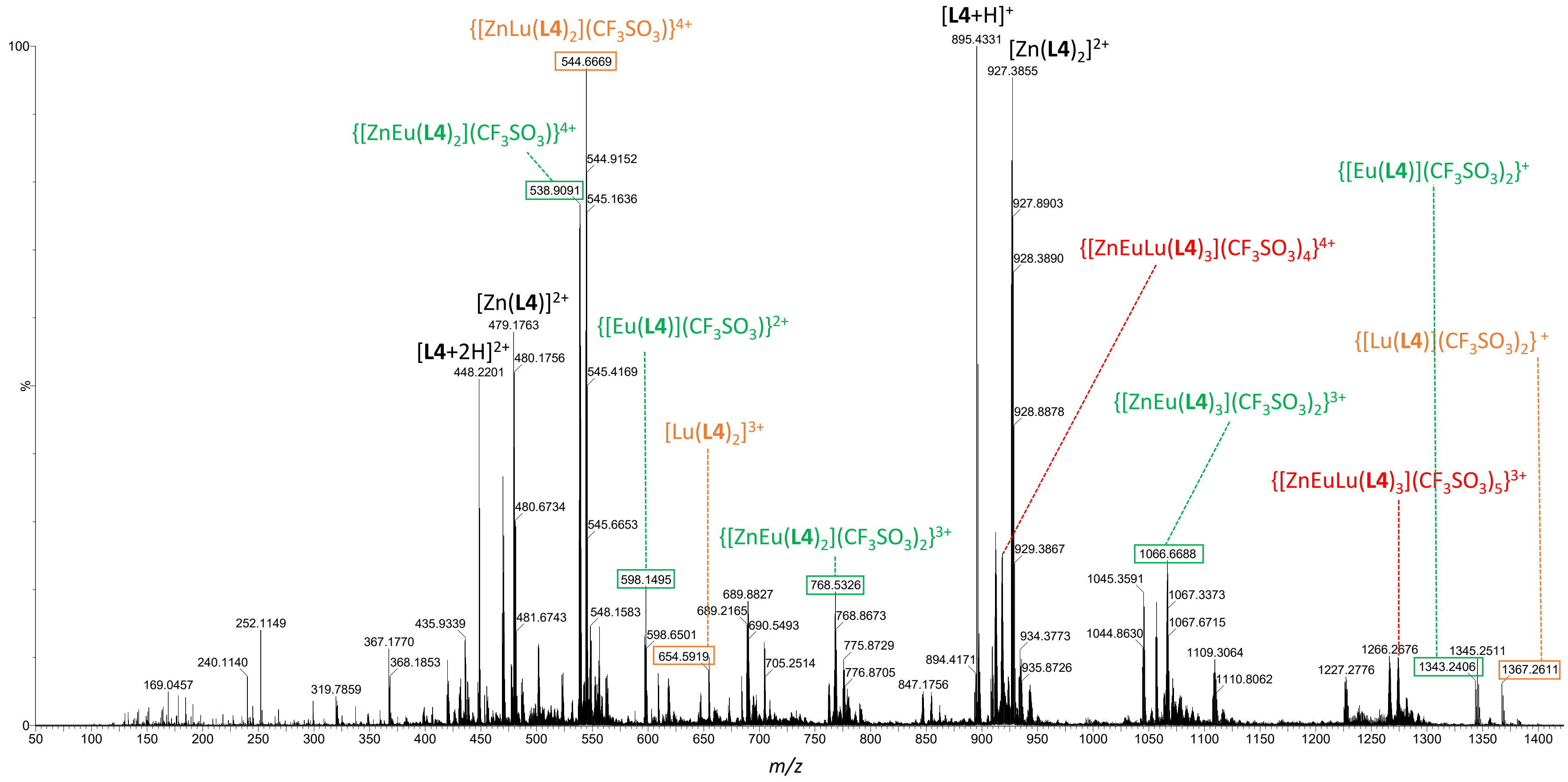

Fig. S39. High-resolution ESI-TOF spectrum of a 1:1:1:3 mixture of $Zn(CF_3SO_3)_2$ (0.51 mM), $Eu(CF_3SO_3)_3$ (0.50 mM), $Lu(CF_3SO_3)_3$ (0.50 mM) and L4 (1.5 mM) in a 2:1 mixture of $CD_3CN/CDCl_3$.

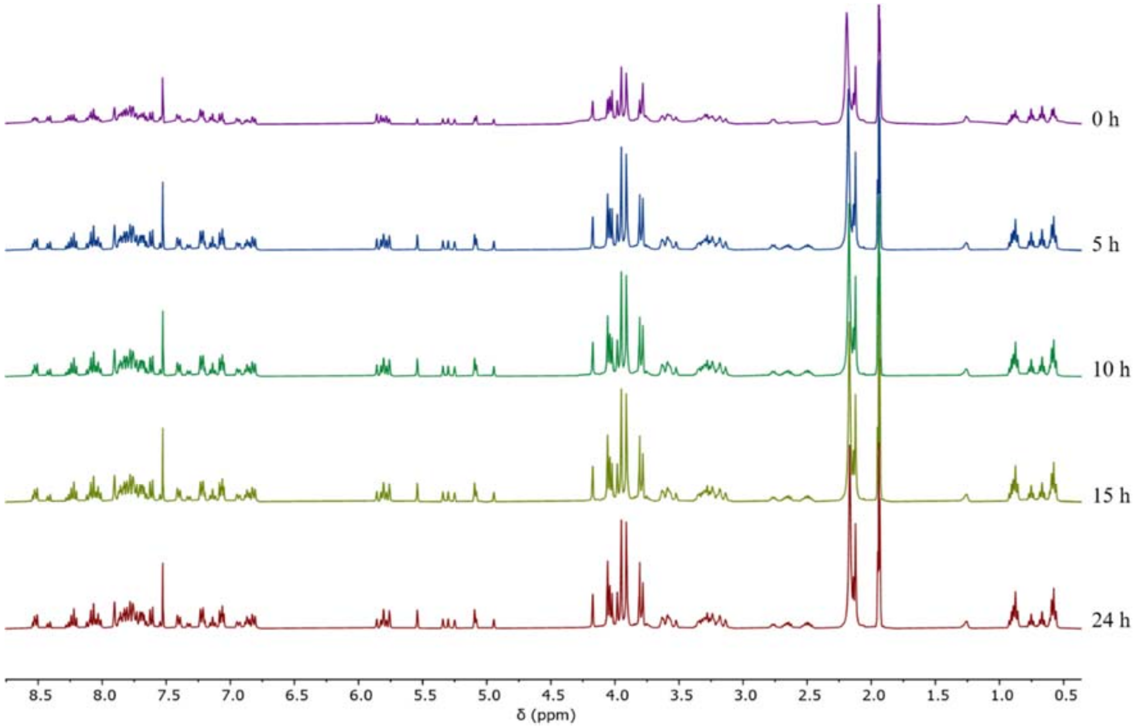

Fig. S40 Evolution of the ^{1}H-NMR spectrum (400 MHz, 2:1 $CD_3CN/CDCl_3$, 298 K) of a 1:1:1:3 mixture of $Zn(CF_3SO_3)_2$, $La(CF_3SO_3)_3$, $Lu(CF_3SO_3)_3$ and **L4** over 24 hours.

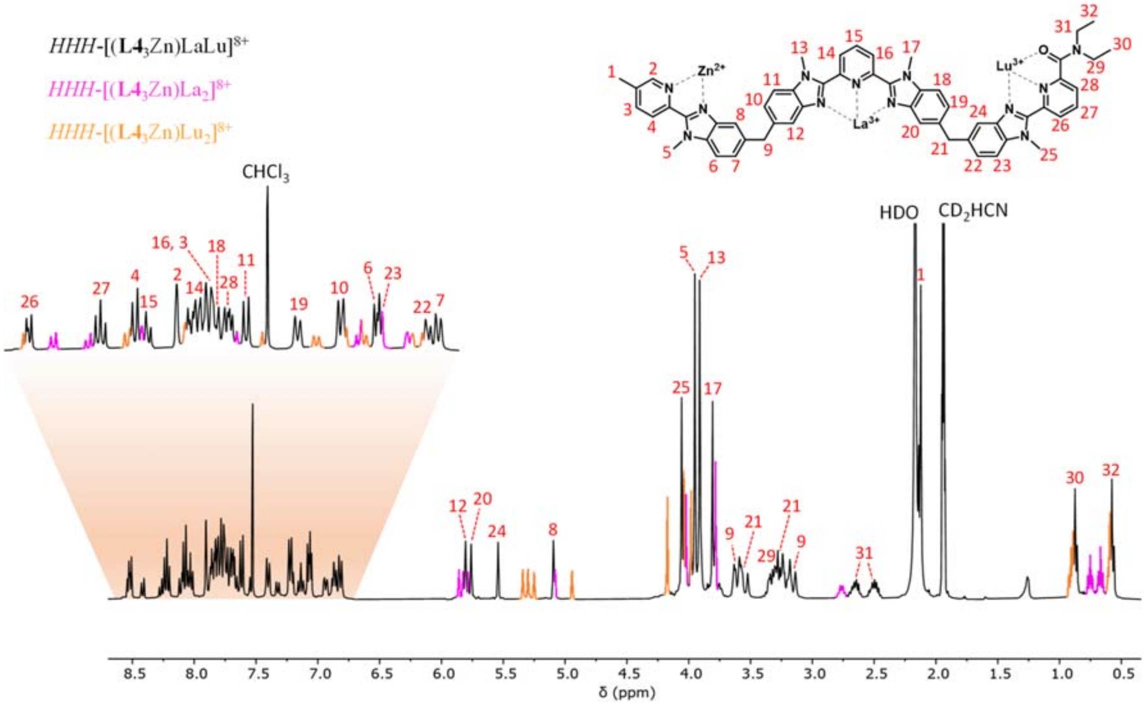

Fig. S41 ^{1}H-NMR spectrum (2:1 $CD_3CN/CDCl_3$, 400 MHz, 298 K) of 1:1:1:3 mixture of $Zn(CF_3SO_3)_2$, $La(CF_3SO_3)_3$, $Lu(CF_3SO_3)_3$ and **L4** at equilibrium. The aromatic region was expanded for clarity.

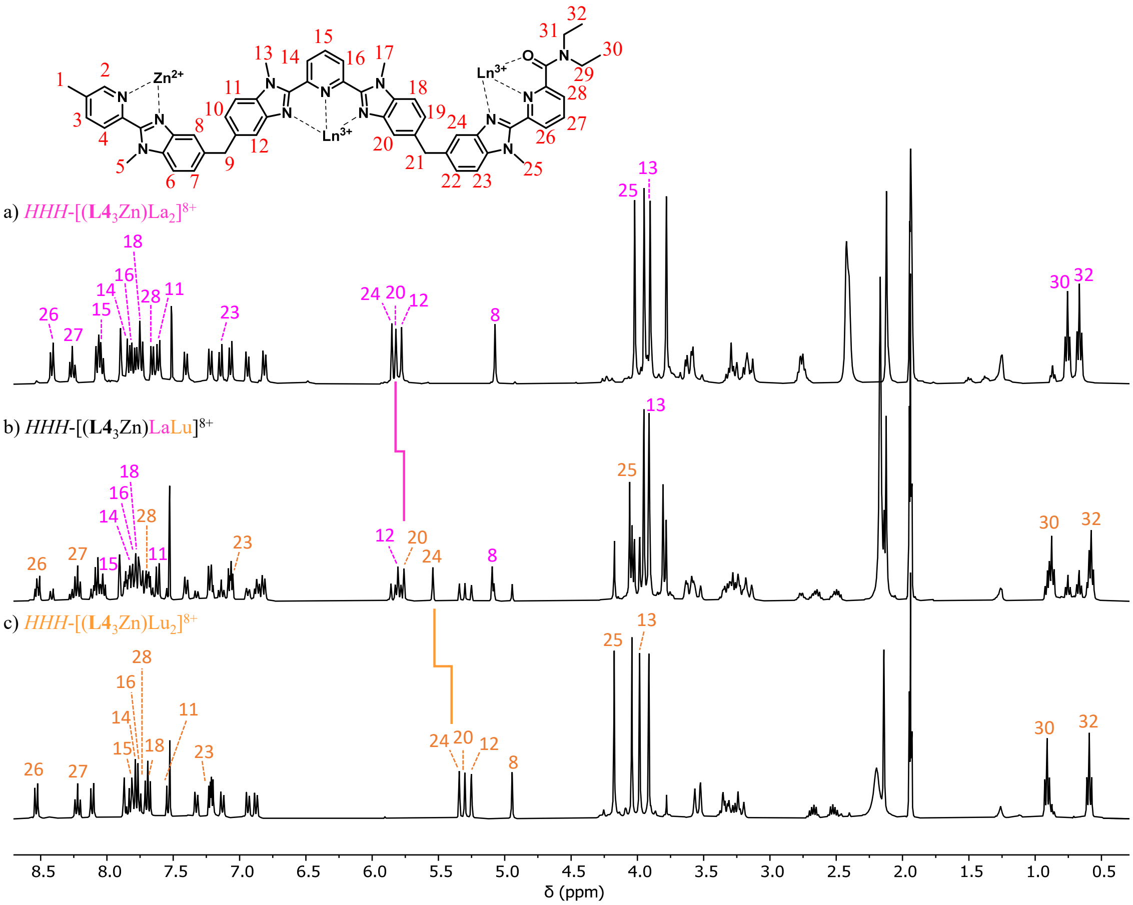

Fig. S42 Comparison of the ^{1}H-NMR spectrum of a) *HHH*-[(**L4**$_3$Zn)La$_2$]$^{8+}$, b) 1:1:1:3 mixture of Zn(CF$_3$SO$_3$)$_2$, La(CF$_3$SO$_3$)$_3$, Lu(CF$_3$SO$_3$)$_3$ and **L4** at equilibrium and c) *HHH*-[(**L4**$_3$Zn)Lu$_2$]$^{8+}$ highlighting some protons (20, 24) relevant to the determination of the position of the lanthanide ions in each site (400 MHz, 2:1 CD$_3$CN/CDCl$_3$, 298 K).

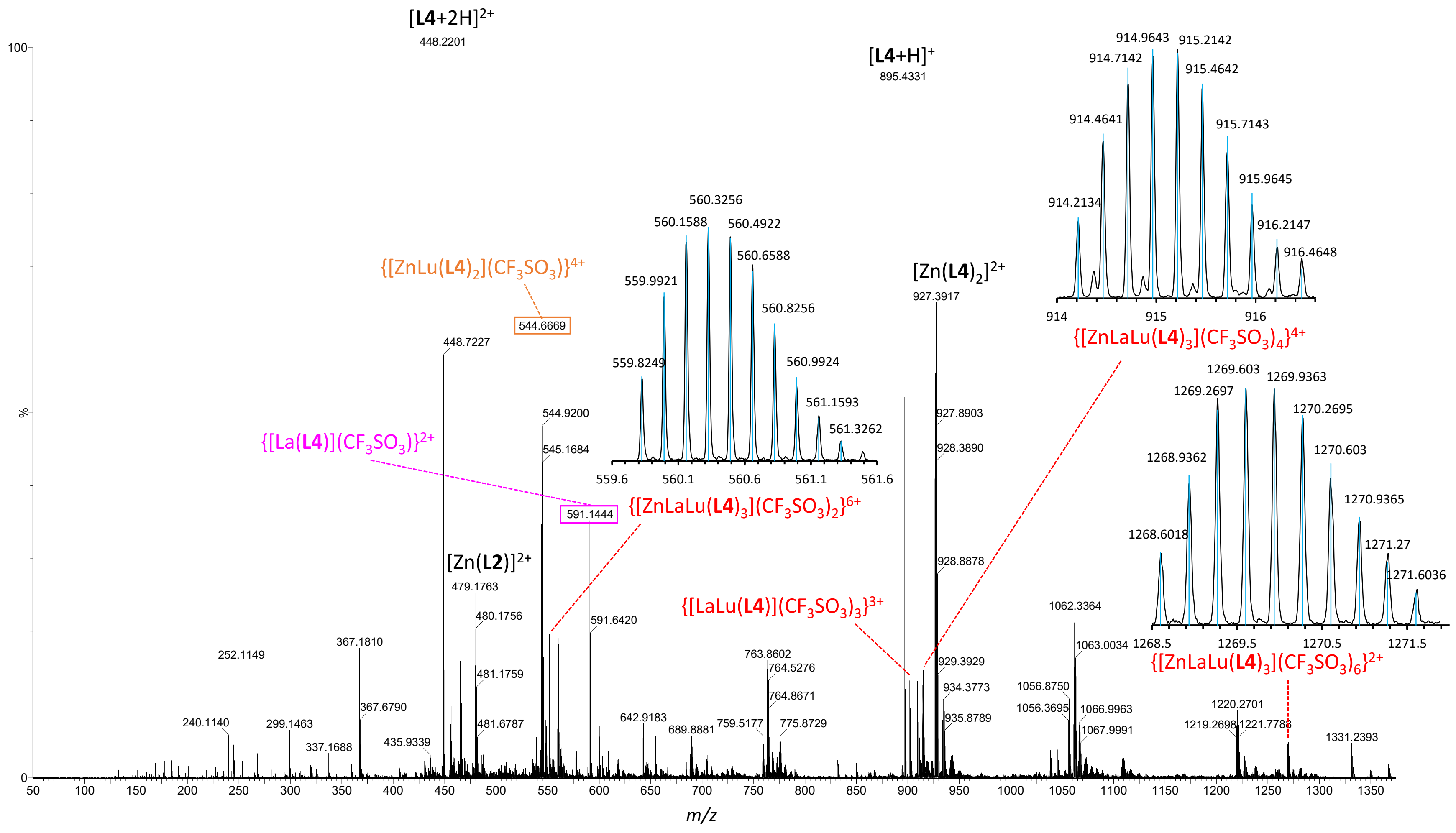

Fig. S43. High-resolution ESI-TOF spectrum of a 1:1:1:3 mixture of $Zn(CF_3SO_3)_2$ (0.51 mM), $La(CF_3SO_3)_3$ (0.49 mM), $Lu(CF_3SO_3)_3$ (0.54 mM) and **L4** (1.6 mM) in a 2:1 mixture of $CD_3CN/CDCl_3$.

Table S28 Speciation (mole fraction) at equilibrium following the reaction of **L5** (3.0 eq) with a mixture of $Ln^{A}(ClO_4)_3$ (1.0 eq) and $Ln^{B}(ClO_4)_3$ (1.0 eq) and thermodynamic descriptors and related free energies associated with the permutation (eqn 1) and exchange (eqn 2) equilibria ($CDCl_3/CD_3CN$ = 1:9, 293 K).[65]

Ln^{A}-Ln^{B}	LaEu	LaTb	LaLu	EuTb	EuLu	TbLu
$\Delta R_{CN=9}^{Ln^A,Ln^B}$ /Å	0.096	0.121	0.184	0.025	0.088	0.063
$x([(\mathbf{L5}_3)Ln^{A}_2]^{6+})$	25(2)	36(2)	35(2)	33(2)	35(2)	35(2)
$x([(\mathbf{L5}_3)Ln^{B}_2]^{6+})$	25(2)	36(2)	35(2)	33(2)	35(2)	35(2)
$x([(\mathbf{L5}_3)Ln^{A}Ln^{B}]^{6+})$	50(2)	28(2)	30(2)	34(2)	30(2)	30(2)
$K_{perm}^{\mathbf{L5}_3,Ln^A,Ln^B}$	1.00	1.00	1.00	1.00	1.00	1.00
$\Delta G_{perm}^{\mathbf{L5}_3,Ln^A,Ln^B}$ /kJ·mol^{-1}	0.00	0.00	0.00	0.00	0.00	0.00
$K_{exch}^{\mathbf{L5}_3,Ln^A,Ln^B}$	4.0(5)	0.60(8)	0.73(9)	1.1(1)	0.73(9)	0.73(9)
$\Delta G_{exch}^{\mathbf{L5}_3,Ln^A,Ln^B}$ /kJ·mol^{-1}	-3.4(3)	1.2(3)	0.8(3)	-0.2(3)	0.8(3)	0.8(3)
$u_{1\text{-}2}^{mix}$	1.0(6)	0.39(2)	0.43(3)	0.52(3)	0.43(3)	0.43(3)
$\Delta E_{1\text{-}2}^{mix}$ /kJ·mol^{-1}	0.0(2)	2.3(2)	2.1(2)	1.6(2)	2.1(2)	2.1(2)

Table S29 Thermodynamic descriptors and related free energies associated with the permutation (eqn 1) and exchange (eqn 2) equilibria following the reaction of **L6** (3.0 eq) with a mixture of $Ln^{A}(CF_3SO_3)_3$ and $Ln^{B}(CF_3SO_3)_3$ $Ln^{A}(CF_3SO_3)_3$ and $Ln^{B}(CF_3SO_3)_3$ (Ln^{A} + Ln^{B} = 2.0 eq) in CD_3CN at 293 K.[83]

Ln^{A}-Ln^{B}	LaLu	LaYb	LaY	LaEu	LaSm	LaNd	NdEu	SmEu	EuLu	EuYb
$\Delta R_{CN=9}^{Ln^A,Ln^B}$ /Å	0.18	0.17	0.14	0.10	0.08	0.05	0.043	0.012	0.088	0.078
$K_{perm}^{\mathbf{L6}_3,Ln^A,Ln^B}$	1.0	1.0	1.0	1.0	1.0	1.0	1.0	1.0	1.0	1.0
$\Delta G_{perm}^{\mathbf{L6}_3,Ln^A,Ln^B}$ /kJ·mol^{-1}	0.0	0.0	0.0	0.0	0.0	0.0	0.0	0.0	0.0	0.0
$K_{exch}^{\mathbf{L6}_3,Ln^A,Ln^B}$	4.0(3)	3.8(3)	4.1(3)	4.3(4)	4.1(3)	4.2(3)	4.1(4)	3.8(3)	4.2(4)	4.2(4)
$\Delta G_{exch}^{\mathbf{L6}_3,Ln^A,Ln^B}$ /kJ·mol^{-1}	-3.4(2)	-3.3(2)	-3.5(2)	-3.5(2)	-3.4(2)	-3.5(2)	-3.5(2)	-3.3(2)	-3.5(2)	-3.5(2)
$u_{1\text{-}2}^{mix}$	1.00(4)	0.98(4)	1.02(4)	1.03(4)	1.01(4)	1.03(4)	1.02(4)	0.98(4)	1.03(4)	1.02(4)
$\Delta E_{1\text{-}2}^{mix}$ /kJ·mol^{-1}	0.0(1)	0.1(1)	-0.0(1)	-0.1(1)	-0.0(1)	-0.1(1)	-0.0(1)	0.1(1)	-0.1(1)	-0.1(1)

Appendix 3: Thermodynamics of heterolanthanide polynuclear helicates in solution.

Since the symmetrical dinuclear [(**L5**$_3$)(Ln^A)$_{(2-n)}$(Ln^B)$_n$]$^{6+}$ (n = 0, 1, 2; Fig. S29b and Table S28)[65] and [(**L6**$_3$)(Ln^A)$_{(2-n)}$(Ln^B)$_n$]$^{6+}$ (n = 0, 1, 2; Fig. S29a and Table S29) helicates[66,83] possess two identical nine-coordinated binding sites, $K_{\text{perm}}^{\mathbf{L5}_3,\text{Ln}^A,\text{Ln}^B} = K_{\text{perm}}^{\mathbf{L6}_3,\text{Ln}^A,\text{Ln}^B} = 1$ for all lanthanide pairs and the exchange energy computed in eqn (8) reduces to $K_{\text{exch}}^{\mathbf{L5}_3,\text{Ln}^A,\text{Ln}^B} = K_{\text{exch}}^{\mathbf{L6}_3,\text{Ln}^A,\text{Ln}^B} = 4\left(u_{1-2}^{\text{mix}}\right)^2$. The [(**L6**$_3$)(Ln^A)$_{(2-n)}(Ln^B)_n$]$^{6+}$ helicates represents the most simple case since one systematically obtains $u_{1-2}^{\text{mix}} = 1$ for any lanthanide pairs (Table S29) and concludes that the two adjacent N_6O_3 binding units are not able to induce deviations from statistical mixtures in solution ($\Delta G_{\text{exch}}^{\text{stat}} = -RT\ln(4)$ = -3.4 kJ/mol; Fig. A3-1a).

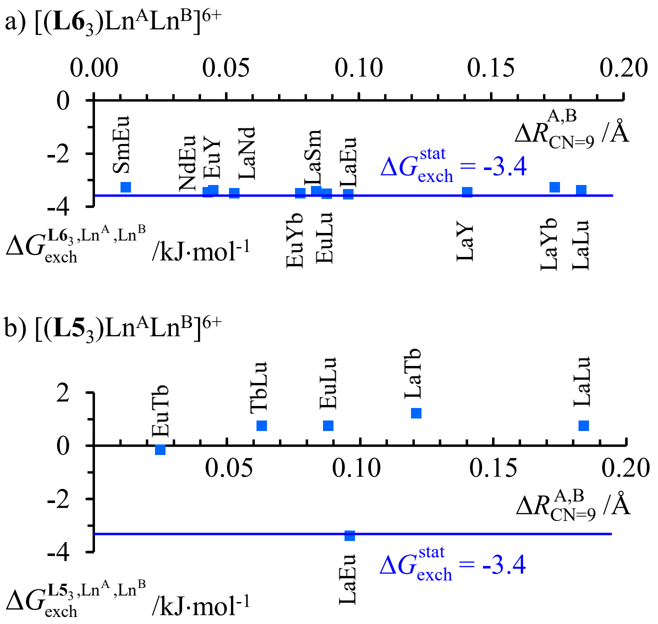

Fig. A3-1 Free energies for permutation ($\Delta G_{\text{perm}}^{\text{Ln}^A,\text{Ln}^B}$ in eqn 1, orange markers) and for exchange ($\Delta G_{\text{exch}}^{\text{Ln}^A,\text{Ln}^B}$ in eqn 2, blue markers) observed in solution at room temperature for a) [(**L6**$_3$)(Ln^A)(Ln^B)]$^{6+}$ (Table S28)[83] and b) [(**L5**$_3$)(Ln^A)(Ln^B)]$^{6+}$ [65] (Table S27) as a function of the difference of the nine-coordinate lanthanide ionic radii ($\Delta R_{\text{CN=9}}^{\text{A,B}}$).[51] The full traces correspond to statistical behaviours.

The situation is more contrasted in [(**L5**$_3$)(Ln^A)$_{(2-n)}$(Ln^B)$_n$]$^{6+}$ because the two connected N_9 sites induce positive mixing energies $\Delta E_{1\text{-}2}^{\text{Ln}^A,\text{Ln}^B} > \frac{1}{2}\left(\Delta E_{1\text{-}2}^{\text{Ln}^A,\text{Ln}^A} + \Delta E_{1\text{-}2}^{\text{Ln}^B,\text{Ln}^B}\right)$ which favor homometallic

matching ($-0.2 \leq \Delta G_{\text{exch}}^{\mathbf{L5}_3,\text{Ln}^\text{A},\text{Ln}^\text{B}} \leq 1.2$ kJ/mol, Fig. A3-1b) beyond statistical distributions as long as at least one lanthanide of the metallic pairs belongs to the second half of the lanthanide series (Table S28). In fact, the binding of a lanthanide Ln^A, smaller than Gd^{3+}, within the constrained N_9 cavity made of three wrapped polyaromatic 2,6-bis(benzimidazole-2-yl)pyridine units produces sufficient distortions for reducing the affinity for the fixation of any Ln^i partner in the adjacent N_9 site in $[(\mathbf{L5}_3)(\text{Ln}^\text{A})(\text{Ln}^i)]^{6+}$.[84] This penalty is paid once for $[(\mathbf{L5}_3)(\text{Ln}^\text{A})(\text{Ln}^\text{A})]^{6+}$ in the mixture of the homolanthanide species (left part of equilibrium 2), but twice for the heterolanthanide $[(\mathbf{L5}_3)(\text{Ln}^\text{A})(\text{Ln}^\text{B})]^{6+}$ and $[(\mathbf{L5}_3)(\text{Ln}^\text{B})(\text{Ln}^\text{A})]^{6+}$ complexes (right part of equilibrium 2). The formation of excesses of homolanthanide complexes is thus observed in $[(\mathbf{L5}_3)(\text{La})_{(2-n)}(\text{Lu})_n]^{6+}$ and $[(\mathbf{L5}_3)(\text{Eu})_{(2-n)}(\text{Lu})_n]^{6+}$, but not for $[(\mathbf{L5}_3)(\text{La})_{(2-n)}(\text{Eu})_n]^{6+}$ (Fig. A3-1b).

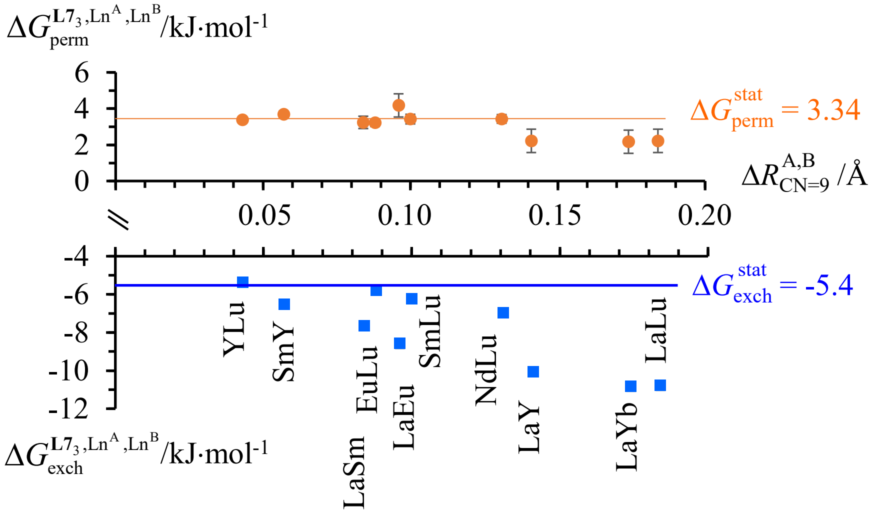

Fig. A3-2 Free energies for permutation ($\Delta G_{\text{perm}}^{\mathbf{L7}_3,\text{Ln}^\text{A},\text{Ln}^\text{B}}$ in eqn A3-1, orange markers) and for exchange ($\Delta G_{\text{exch}}^{\mathbf{L7}_3,\text{Ln}^\text{A},\text{Ln}^\text{B}}$ in eqn A3-2, blue markers) observed for $[(\mathbf{L7}_3)(\text{Ln}^\text{A})_{(3-n)}(\text{Ln}^\text{B})_n]^{9+}$ (n = 0, 1, 2, 3) in acetonitrile at room temperature[11] as a function of the difference of the nine-coordinate lanthanide ionic radii ($\Delta R_{\text{CN=9}}^{\text{A,B}}$).[51] The full traces correspond to statistical behaviors.

Interestingly, a sequence of adjacent/alternating N_9 and N_6O_3 binding sites has a precedent in the extended symmetrical trinuclear $[(\mathbf{L7}_3)(\text{Ln}^\text{A})_{(3-n)}(\text{Ln}^\text{B})_n]^{9+}$ (n = 0, 1, 2, 3) helicates (Fig. S29c),[11] for which the pertinent permutation equilibrium (A3-1) now simply reflects the balance of the

intermetallic interactions $K_{\text{perm}}^{\mathbf{L7}_3,\text{Ln}^\text{A},\text{Ln}^\text{B}} = \left(u_{1-2}^{\text{mix}}\right)^2 / 4$ (the justification is shown as eqn A4-1 in Appendix 4).

$$[(\text{Ln}^\text{A})(\text{Ln}^\text{A})(\text{Ln}^\text{B})] + [(\text{Ln}^\text{B})(\text{Ln}^\text{B})(\text{Ln}^\text{A})] \rightleftharpoons [(\text{Ln}^\text{A})(\text{Ln}^\text{B})(\text{Ln}^\text{A})] + [(\text{Ln}^\text{B})(\text{Ln}^\text{A})(\text{Ln}^\text{B})] \quad K_{\text{perm}}^{\mathbf{L7}_3,\text{Ln}^\text{A},\text{Ln}^\text{B}} \quad \text{(A3-1)}$$

The observed experimental free energy changes $\Delta G_{\text{perm}}^{\mathbf{L7}_3,\text{Ln}^\text{A},\text{Ln}^\text{B}} = -RT\ln\left(K_{\text{perm}}^{\mathbf{L7}_3,\text{Ln}^\text{A},\text{Ln}^\text{B}}\right)$ do not deviate significantly from the statistical value $\Delta G_{\text{perm,stat}}^{\mathbf{L7}_3,\text{Ln}^\text{A},\text{Ln}^\text{B}} = -RT\ln\left(1/4\right)$ = 3.34 kJ/mol (Fig. A3-2, orange markers and trace), which translates into negligible mixing energies $\Delta E_{1\text{-}2}^{\text{mix}}$ = -0.1(3) kJ/mol (Table A4-1 in Appendix 4). One immediately concludes that any unbalanced lanthanide-dependent intermetallic pair interactions $\Delta E_{1\text{-}2}^{\text{mix}} \neq 0$, as found in $[(\mathbf{L4}_3\text{Zn})(\text{Ln}^\text{A})_{(3\text{-}n)}(\text{Ln}^\text{B})_n]^{8+}$, are thus not the simple repercussion of a sequence of two connected adjacent N_9 and N_6O_3 sites in these triple-stranded helical complexes. The supplementary connection of the N_9-N_6O_3 sequence to the charged non-covalent *HHH*-$[\text{Zn}\mathbf{L4}_3]^{2+}$ tripod appears to be crucial for boosting the formation of the heterolanthanide isomers. Returning to the symmetrical trinuclear helicates $[(\mathbf{L7}_3)(\text{Ln}^\text{A})_{(3\text{-}n)}(\text{Ln}^\text{B})_n]^{9+}$, one can then foresee that any size-discriminating effect relies entirely on the variable affinities of the N_9 and N_6O_3 binding sites for each specific entering lanthanide, a trend measured by the exchange free energies -10.8(5) ≤ $\Delta G_{\text{exch}}^{\mathbf{L7}_3,\text{Ln}^\text{A},\text{Ln}^\text{B}} = -RT\ln\left(K_{\text{exch}}^{\mathbf{L7}_3,\text{Ln}^\text{A},\text{Ln}^\text{B}}\right)$ ≤ -5.4(3) kJ/mol defined in eqn (A3-2).

$$[(\text{Ln}^\text{A})_3] + [(\text{Ln}^\text{B})_3] \rightleftharpoons [(\text{Ln}^\text{A})_2(\text{Ln}^\text{B})] + [(\text{Ln}^\text{A})(\text{Ln}^\text{B})_2] \quad K_{\text{exch}}^{\mathbf{L7}_3,\text{Ln}^\text{A},\text{Ln}^\text{B}} \quad \text{(A3-2)}$$

The latter values increasingly deviate from the statistical distribution $\Delta G_{\text{exch,stat}}^{\mathbf{L7}_3,\text{Ln}^\text{A},\text{Ln}^\text{B}} = -RT\ln\left(9\right)$ = -5.4 kJ/mol (the justification is shown as eqn A4-2 in Appendix 4) toward the preferred formation of the heterolanthanide trinuclear helicates when the difference between the lanthanide ionic size increases (Fig. A3-2, blue markers). This trend is further amplified in the tetranuclear $[(\mathbf{L8}_3)\text{La}_{(4\text{-}n)}(\text{Lu})_n]^{12+}$ (n = 0, 1, 2, 3,4 Fig. S29d) helicates, for which an exchange energy of $\Delta G_{\text{exch}}^{\mathbf{L8}_3,\text{La,Lu}}$ = -47.7(5)

kJ/mol has been reported for the La:Lu pair in acetonitrile at room temperature (eqn A3-3, statistical distribution $\Delta G_{\text{exch,stat}}^{\mathbf{L8}_3\text{,La,Lu}} = -RT\ln\left(4^2 \cdot 6^2 \cdot 4^2\right) = -RT\ln\left(9216\right)$ = -22.2 kJ/mol).[12]

$$3[(Ln^A)_4] + 3[(Ln^B)_4] \rightleftharpoons 2[(Ln^A)_3(Ln^B)] + 2[(Ln^A)_2(Ln^B)_2] + 2[(Ln^A)(Ln^B)_3] \quad K_{\text{exch}}^{\mathbf{L8}_3\text{,Ln}^A\text{,Ln}^B} \quad \text{(A3-3)}$$

The sequence of adjacent nine-coordinating binding sites N_6O_3-N_9-N_9-N_6O_3 in the tetranuclear $[(\mathbf{L8}_3)La_{(4-n)}(Lu)_n]^{12+}$ helicate (Fig. S29d) provides an adjacent N_9-N_9 pair, which is lacking in the trinuclear $[(\mathbf{L7}_3)(La)_{(3-n)}(Lu)_n]^{9+}$ (Fig. S29c), but which is reminiscent of that found in the dinuclear $[(\mathbf{L5}_3)La_{(2-n)}Lu_n]^{6+}$ helicate (Fig. S29a). In this context, it is worth noting that the positive intermetallic mixing energy $\Delta E_{1\text{-}2}^{\text{mix}} = 2.1(2)$ kJ/mol observed for the latter dinuclear helicate (Table S28)[65] contrasts with the negative $\Delta E_{1\text{-}2}^{\text{mix}}$ = -2.0 kJ/mol reported for the tetranuclear $[(\mathbf{L8}_3)La_{(4-n)}(Lu)_n]^{12+}$ helicate.[12]

Appendix 4: Thermodynamic modeling and analysis of heterometallic trinuclear [(L7₃)(Ln^A)₍₃₋ₙ₎(Ln^B)ₙ]^9+ helicates.

At millimolar concentrations, the lack of detectable dissociation of $[(\mathbf{L7}_3)(\mathrm{Ln^A})_{(3-n)}(\mathrm{Ln^B})_n]^{9+}$ in solution under stoichiometric conditions allows the consideration of $[\mathbf{L7}_3]$ as a preorganized receptor pertinent to the associated microscopic formation constants collected in Scheme A4-1.[11]

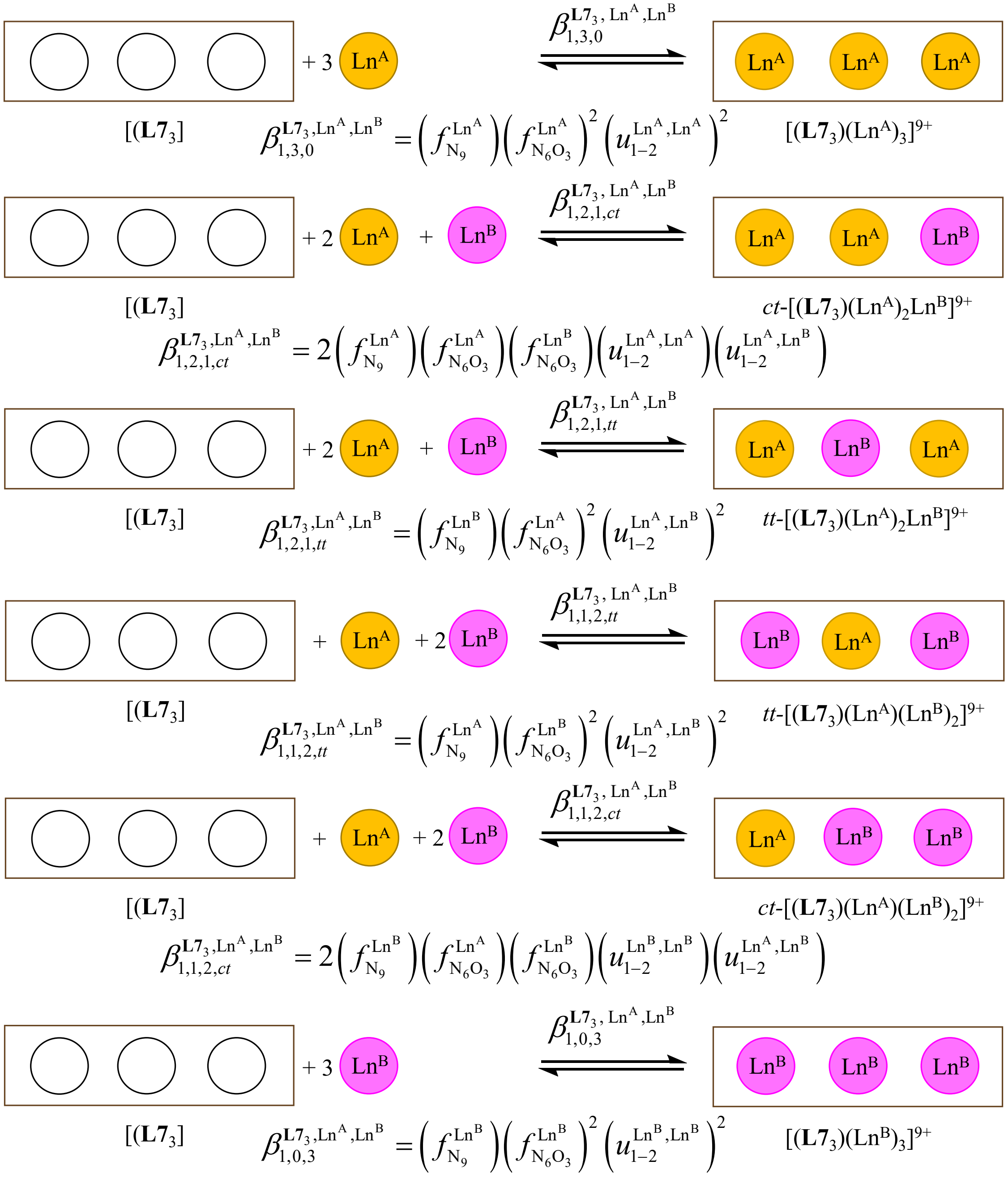

$$\beta_{1,3,0}^{\mathbf{L7}_3,\mathrm{Ln^A},\mathrm{Ln^B}} = \left(f_{\mathrm{N_9}}^{\mathrm{Ln^A}}\right)\left(f_{\mathrm{N_6O_3}}^{\mathrm{Ln^A}}\right)^2\left(u_{1-2}^{\mathrm{Ln^A},\mathrm{Ln^A}}\right)^2$$

$$\beta_{1,2,1,ct}^{\mathbf{L7}_3,\mathrm{Ln^A},\mathrm{Ln^B}} = 2\left(f_{\mathrm{N_9}}^{\mathrm{Ln^A}}\right)\left(f_{\mathrm{N_6O_3}}^{\mathrm{Ln^A}}\right)\left(f_{\mathrm{N_6O_3}}^{\mathrm{Ln^B}}\right)\left(u_{1-2}^{\mathrm{Ln^A},\mathrm{Ln^A}}\right)\left(u_{1-2}^{\mathrm{Ln^A},\mathrm{Ln^B}}\right)$$

$$\beta_{1,2,1,tt}^{\mathbf{L7}_3,\mathrm{Ln^A},\mathrm{Ln^B}} = \left(f_{\mathrm{N_9}}^{\mathrm{Ln^B}}\right)\left(f_{\mathrm{N_6O_3}}^{\mathrm{Ln^A}}\right)^2\left(u_{1-2}^{\mathrm{Ln^A},\mathrm{Ln^B}}\right)^2$$

$$\beta_{1,1,2,tt}^{\mathbf{L7}_3,\mathrm{Ln^A},\mathrm{Ln^B}} = \left(f_{\mathrm{N_9}}^{\mathrm{Ln^A}}\right)\left(f_{\mathrm{N_6O_3}}^{\mathrm{Ln^B}}\right)^2\left(u_{1-2}^{\mathrm{Ln^A},\mathrm{Ln^B}}\right)^2$$

$$\beta_{1,1,2,ct}^{\mathbf{L7}_3,\mathrm{Ln^A},\mathrm{Ln^B}} = 2\left(f_{\mathrm{N_9}}^{\mathrm{Ln^B}}\right)\left(f_{\mathrm{N_6O_3}}^{\mathrm{Ln^A}}\right)\left(f_{\mathrm{N_6O_3}}^{\mathrm{Ln^B}}\right)\left(u_{1-2}^{\mathrm{Ln^B},\mathrm{Ln^B}}\right)\left(u_{1-2}^{\mathrm{Ln^A},\mathrm{Ln^B}}\right)$$

$$\beta_{1,0,3}^{\mathbf{L7}_3,\mathrm{Ln^A},\mathrm{Ln^B}} = \left(f_{\mathrm{N_9}}^{\mathrm{Ln^B}}\right)\left(f_{\mathrm{N_6O_3}}^{\mathrm{Ln^B}}\right)^2\left(u_{1-2}^{\mathrm{Ln^B},\mathrm{Ln^B}}\right)^2$$

Scheme A4-1 Microscopic thermodynamic formation constants for $[(\mathbf{L7}_3)(\mathrm{Ln^A})_{(3-n)}(\mathrm{Ln^B})_n]^{9+}$ (n = 0, 1, 2, 3) and their modeling with the site binding model.[11] See main text for the definitions of $f_i^{\mathrm{Ln}^j}$ and $u_{1-2}^{\mathrm{Ln}^i,\mathrm{Ln}^j}$.

The thermodynamic permutation (eqn A3-1) and exchange (eqn A3-2) processes pertinent to the trinuclear helicates $[(\mathbf{L7}_3)(\mathrm{Ln^A})_{(3\text{-}n)}(\mathrm{Ln^B})_n]^{9+}$ (n = 0, 1, 2, 3) are gathered in Table A4-1 and can be modelled with the site binding model thanks to eqn (A4-1) and eq (A4-2).[11]

$$[(\mathrm{Ln^A})(\mathrm{Ln^A})(\mathrm{Ln^B})] + [(\mathrm{Ln^B})(\mathrm{Ln^B})(\mathrm{Ln^A})] \rightleftharpoons [(\mathrm{Ln^A})(\mathrm{Ln^B})(\mathrm{Ln^A})] + [(\mathrm{Ln^B})(\mathrm{Ln^A})(\mathrm{Ln^B})] \qquad K_{\mathrm{perm}}^{\mathbf{L7}_3,\mathrm{Ln^A},\mathrm{Ln^B}} \qquad \text{(A3-1)}$$

$$K_{\mathrm{perm}}^{\mathbf{L7}_3,\mathrm{Ln^A},\mathrm{Ln^B}} = \frac{\beta_{1,2,1,tt}^{\mathbf{L7}_3,\mathrm{Ln^A},\mathrm{Ln^B}}\,\beta_{1,1,2,tt}^{\mathbf{L7}_3,\mathrm{Ln^A},\mathrm{Ln^B}}}{\beta_{1,2,1,ct}^{\mathbf{L7}_3,\mathrm{Ln^A},\mathrm{Ln^B}}\,\beta_{1,1,2,ct}^{\mathbf{L7}_3,\mathrm{Ln^A},\mathrm{Ln^B}}} = \frac{1}{4}\frac{\left(u_{1-2}^{\mathrm{Ln^A},\mathrm{Ln^B}}\right)^2}{u_{1-2}^{\mathrm{Ln^A},\mathrm{Ln^A}}\cdot u_{1-2}^{\mathrm{Ln^B},\mathrm{Ln^B}}} = \frac{1}{4}\left(u_{1-2}^{\mathrm{mix}}\right)^2 \qquad \text{(A4-1)}$$

$$[(\mathrm{Ln^A})_3] + [(\mathrm{Ln^B})_3] \rightleftharpoons [(\mathrm{Ln^A})_2(\mathrm{Ln^B})] + [(\mathrm{Ln^A})(\mathrm{Ln^B})_2] \qquad K_{\mathrm{exch}}^{\mathbf{L7}_3,\mathrm{Ln^A},\mathrm{Ln^B}} \qquad \text{(A3-2)}$$

$$K_{\mathrm{exch}}^{\mathbf{L7}_3,\mathrm{Ln^A},\mathrm{Ln^B}} = \frac{\left[\left(f_{\mathrm{N_9}}^{\mathrm{Ln^B}}\right)\left(f_{\mathrm{N_6O_3}}^{\mathrm{Ln^A}}\right)u_{1-2}^{\mathrm{Ln^A},\mathrm{Ln^B}} + 2\left(f_{\mathrm{N_9}}^{\mathrm{Ln^A}}\right)\left(f_{\mathrm{N_6O_3}}^{\mathrm{Ln^B}}\right)u_{1-2}^{\mathrm{Ln^A},\mathrm{Ln^A}}\right]}{\left(f_{\mathrm{N_9}}^{\mathrm{Ln^A}}\right)\left(f_{\mathrm{N_6O_3}}^{\mathrm{Ln^A}}\right)\left(f_{\mathrm{N_9}}^{\mathrm{Ln^B}}\right)\left(f_{\mathrm{N_6O_3}}^{\mathrm{Ln^B}}\right)\left(u_{1-2}^{\mathrm{Ln^A},\mathrm{Ln^A}}\right)\left(u_{1-2}^{\mathrm{Ln^B},\mathrm{Ln^B}}\right)} \times\left[\left(f_{\mathrm{N_9}}^{\mathrm{Ln^A}}\right)\left(f_{\mathrm{N_6O_3}}^{\mathrm{Ln^B}}\right)u_{1-2}^{\mathrm{Ln^A},\mathrm{Ln^B}} + 2\left(f_{\mathrm{N_9}}^{\mathrm{Ln^B}}\right)\left(f_{\mathrm{N_6O_3}}^{\mathrm{Ln^A}}\right)u_{1-2}^{\mathrm{Ln^B},\mathrm{Ln^B}}\right]\left(u_{1-2}^{\mathrm{mix}}\right)^2 \qquad \text{(A4-2)}$$

Under statistical conditions, $f_{\mathrm{N_9}}^{\mathrm{Ln^A}} = f_{\mathrm{N_9}}^{\mathrm{Ln^B}} = f_{\mathrm{N_6O_3}}^{\mathrm{Ln^A}} = f_{\mathrm{N_6O_3}}^{\mathrm{Ln^B}}$ and $u_{1-2}^{\mathrm{Ln^A},\mathrm{Ln^A}} = u_{1-2}^{\mathrm{Ln^A},\mathrm{Ln^B}} = u_{1-2}^{\mathrm{Ln^B},\mathrm{Ln^B}}$. This leads to $K_{\mathrm{perm,stat}}^{\mathbf{L7}_3,\mathrm{Ln^A},\mathrm{Ln^B}} = 0.25$ (eq A4-1) and $K_{\mathrm{exch,stat}}^{\mathbf{L7}_3,\mathrm{Ln^A},\mathrm{Ln^B}} = 9$ (eqn A4-2), which translates into $\Delta G_{\mathrm{perm,stat}}^{\mathbf{L7}_3,\mathrm{Ln^A},\mathrm{Ln^B}} =$ 3.34 kJ/mol and $\Delta G_{\mathrm{exch,stat}}^{\mathbf{L7}_3,\mathrm{Ln^A},\mathrm{Ln^B}} =$ -5.4 kJ/mol at 298 K. The associated experimental values determined for $[(\mathbf{L7}_3)(\mathrm{Ln^A})_{(3\text{-}n)}(\mathrm{Ln^B})_n]^{9+}$ (n = 0, 1, 2, 3) in acetonitrile at room temperature[11] are collected in Table A4-1 and illustrated in Fig. A3-2.

Table A4-1 Thermodynamic descriptors and related free energies associated with the permutation (eqn A3-1) and exchange (eqn A3-2) equilibria following the reaction of **L7** (3.0 eq) with a mixture of $Ln^A(CF_3SO_3)_3$ and $Ln^B(CF_3SO_3)_3$ ($Ln^A + Ln^B$ = 3.0 eq) in CD_3CN at 293 K.[11]

Ln^A-Ln^B	LaLu	LaYb	LaY	NdLu	SmLu	LaEu	EuLu	LaSm	SmY	YLu
$\Delta R_{CN=9}^{Ln^A,Ln^B}$ /Å	0.184	0.174	0.141	0.131	0.100	0.096	0.088	0.084	0.057	0.043
$K_{perm}^{\mathbf{L7}_3,Ln^A,Ln^B}$	0.4(2)	0.4(2)	0.4(2)	0.24(5)	0.25(5)	0.18(9)	0.27(4)	0.26(7)	0.22(3)	0.25(3)
$\Delta G_{perm}^{\mathbf{L7}_3,Ln^A,Ln^B}$ /kJ·mol^{-1}	2.2(1.3)	2.2(1.3)	2.2(1.3)	3.4(5)	3.4(5)	4.2(1.3)	3.2(4)	3.2(7)	3.7(4)	3.4(3)
$K_{exch}^{\mathbf{L7}_3,Ln^A,Ln^B}$	83(18)	85(18)	63(11)	18(3)	13(2)	34(7)	11(1)	23(4)	15(2)	9(1)
$\Delta G_{exch}^{\mathbf{L7}_3,Ln^A,Ln^B}$ /kJ·mol^{-1}	-10.8(5)	-10.8(5)	-10.1(4)	-7.0(4)	-6.2(3)	-8.6(5)	-5.8(3)	-7.7(4)	-6.5(3)	-5.4(3)
u_{1-2}^{mix}	1.3(3)	1.3(3)	1.3(3)	1.0(1)	1.0(1)	0.9(2)	1.03(8)	1.0(2)	0.94(7)	1.00(6)
ΔE_{1-2}^{mix} /kJ·mol^{-1}	-0.6(6)	-0.6(6)	-0.6(6)	0.0(2)	0.0(3)	0.4(6)	-0.1(2)	-0.1(3)	0.2(2)	0.0(2)
